# A single-cell atlas of non-haematopoietic cells in human lymph nodes and lymphoma reveals a landscape of stromal remodelling

**DOI:** 10.1038/s41556-022-00866-3

**Published:** 2022-03-24

**Authors:** Yoshiaki Abe, Mamiko Sakata-Yanagimoto, Manabu Fujisawa, Hiroaki Miyoshi, Yasuhito Suehara, Keiichiro Hattori, Manabu Kusakabe, Tatsuhiro Sakamoto, Hidekazu Nishikii, Tran B. Nguyen, Yohei Owada, Tsuyoshi Enomoto, Aya Sawa, Hiroko Bando, Chikashi Yoshida, Rikako Tabata, Toshiki Terao, Masahiro Nakayama, Koichi Ohshima, Kensuke Usuki, Tatsuya Oda, Kosei Matsue, Shigeru Chiba

**Affiliations:** 1 Department of Hematology, Graduate School of Comprehensive Human Sciences, University of Tsukuba, Tsukuba, Japan; 2 Department of Hematology, Faculty of Medicine, University of Tsukuba, Tsukuba, Japan; 3 Department of Hematology, University of Tsukuba Hospital, Tsukuba, Japan; 4 Division of Advanced Hemato-Oncology, Transborder Medical Research Center, University of Tsukuba, Tsukuba, Japan; 5 Department of Pathology, School of Medicine, Kurume University, Kurume, Japan; 6 Department of Gastrointestinal and Hepato-Biliary-Pancreatic Surgery, Faculty of Medicine, University of Tsukuba, Tsukuba, Japan; 7 Department of Breast-Thyroid-Endocrine Surgery, University of Tsukuba Hospital, Tsukuba, Japan; 8 Department of Breast and Endocrine Surgery, Faculty of Medicine, University of Tsukuba, Tsukuba, Japan; 9 Department of Hematology, National Hospital Organization, Mito Medical Center, Higashiibaraki, Japan; 10 Division of Hematology/Oncology, Department of Internal Medicine, Kameda Medical Center, Kamogawa, Japan; 11 Department of Otolaryngology, Head and Neck Surgery, University of Tsukuba, Tsukuba, Japan; 12 Department of Hematology, NTT Medical Center Tokyo, Tokyo, Japan

**Keywords:** Cancer microenvironment, Tumour heterogeneity, Transcriptomics

## Abstract

The activities of non-haematopoietic cells (NHCs), including mesenchymal stromal cells and endothelial cells, in lymphomas are reported to underlie lymphomagenesis. However, our understanding of lymphoma NHCs has been hampered by unexplained NHC heterogeneity, even in normal human lymph nodes (LNs). Here we constructed a single-cell transcriptome atlas of more than 100,000 NHCs collected from 27 human samples, including LNs and various nodal lymphomas, and it revealed 30 distinct subclusters, including some that were previously unrecognized. Notably, this atlas was useful for comparative analyses with lymphoma NHCs, which revealed an unanticipated landscape of subcluster-specific changes in gene expression and interaction with malignant cells in follicular lymphoma NHCs. This facilitates our understanding of stromal remodelling in lymphoma and highlights potential clinical biomarkers. Our study largely updates NHC taxonomy in human LNs and analysis of disease status, and provides a rich resource and deeper insights into LN and lymphoma biology to advance lymphoma management and therapy.

## Abstract (web summary)

Abe et al. profile, characterize and compare non-haematopoietic cells in normal human lymph nodes versus nodal lymphomas from patients, providing insights into stromal modelling in health and disease.

## Main

Lymphomas are haematological malignancies that often develop from LNs^1^. Despite advances in treatments, most lymphoma subtypes remain incurable. Therefore, new therapeutic approaches are needed, including those that target the tumour microenvironment^2,3^. In lymphomas, as in solid cancers^4,5^, the activities of NHCs, such as mesenchymal stromal cells (SCs) and endothelial cells, are thought to facilitate lymphomagenesis and therefore have potential as therapeutic targets^2,3^. Indeed, some lymphoma subtypes are reported to exhibit unique interactions with NHCs^6–9^; however, lymphoma NHC research is far behind that of solid cancers^10^. In particular, follicular lymphoma (FL) cells are considered to actively interact with NHCs to promote emergence and expansion^9,11,12^. SC-derived CXCL12 recruits FL cells in cooperation with CXCL13 (produced by follicular dendritic cells (FDCs)), which contribute to the follicular localization of tumour cells and their proliferation^9,13^. Other FDC-derived molecules, including BAFF (encoded by *TNFSF13B*), interleukin-15 and HGF, may have anti-apoptotic effects on FL cells^14–16^. Unfortunately, a complete understanding of the temporal and spatial associations that underlie these activities is hampered by the heterogeneity of NHCs. In fact, definitive NHC classification has not yet been achieved in humans, even in normal LNs^17,18^. Moreover, the identification of alterations in LN NHC (LNNHC) heterogeneity in the context of lymphomas is barely underway.

Major subsets of NHCs in LNs, as determined by morphology and topological localization, include blood endothelial cells (BECs), which include high endothelial venules (HEVs), lymphatic endothelial cells (LECs) and non-endothelial SCs (NESCs)^18–20^. Examples of NESCs include T-zone reticular cells (TRCs), medullary reticular cells, perivascular cells and follicular SCs (FSCs), such as FDCs and marginal reticular cells (MRCs)^18–20^. Although recent investigations of NHC heterogeneity have used single-cell RNA sequencing (scRNA-seq) technology^21–26^, human LN BECs and NESCs have yet to be comprehensively analysed at single-cell resolution.

To address this issue, scRNA-seq was used in this study to construct an atlas of human NHCs in LNs and lymphoma. We aimed to identify previously unrecognized NHC heterogeneity in human LNs and to distinguish NHCs from lymphomas to define the global influences of lymphoma cells on the NHC niche. This approach can provide deep insights into lymphoma stromal biology and resources applicable to future studies of lymphomas and identify potential stroma-derived biomarkers that may serve as clinical indicators and/or therapeutic targets.

## Results

### Transcriptional features of major NHC components

**Fig. 1:**
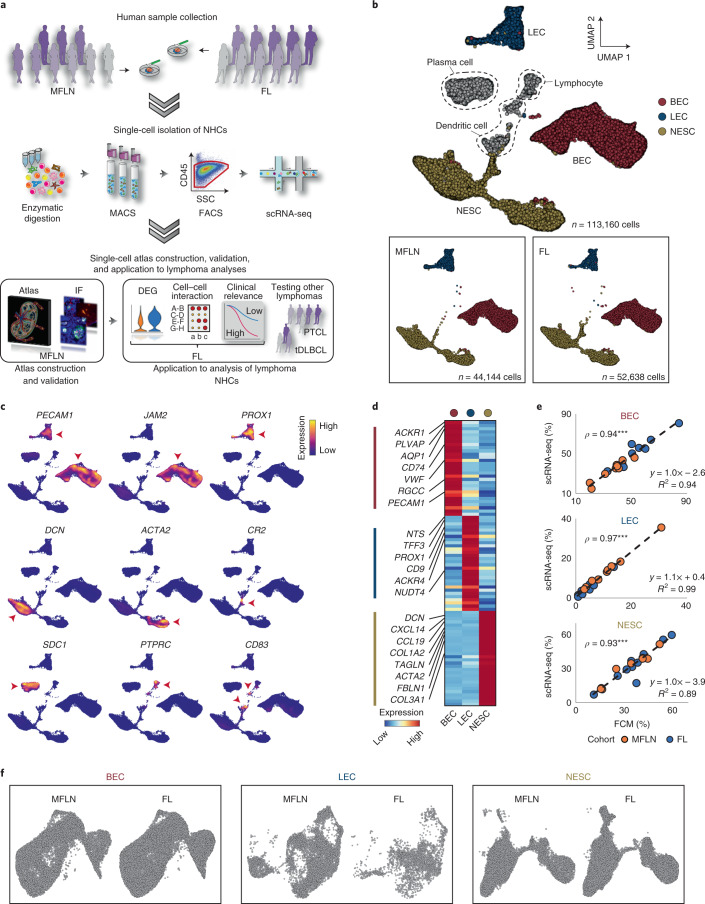
Single-cell survey of NHC components in LNs and FL. **a**, Study overview of the experimental and analytical workflows. FACS, fluorescence-activated cell sorting; MACS, magnetic-activated cell sorting; SSC, side scatter. **b**, UMAP plots of stroma-enriched cells from nine human MFLN samples and ten FL samples, coloured by cell type (top). Major NHC components from MFLN samples and FL samples are shown separately (bottom left and bottom right, respectively). **c**, Expression levels of marker genes used to identify cell types. Red arrowheads show cells expressing the indicated marker genes. **d**, Heatmap showing the expression of top-ranking marker genes for each major NHC component. Key genes are indicated on the left. **e**, Correlation of the proportions of BECs, LECs and NESCs among stroma-enriched cells, as evaluated using flow cytometry (FCM) analysis and scRNA-seq, coloured according to patient cohort. Circles indicate biologically independent samples (*n* = 9 MFLN, *n* = 10 FL). *ρ* denotes Spearman’s rank correlation coefficient. ***P = 4.0 × 10^−6^ (BEC), ***P = 8.0 × 10^−6^ (LEC), ***P = 2.5 × 10^−6^ (NESC) (two-sided Spearman’s rank correlation test). **f**, UMAP plots of LN BECs, LECs and NESCs after re-clustering analysis shown according to patient cohort. Statistical source data are provided. Source data

**Extended Data Fig. 1:**
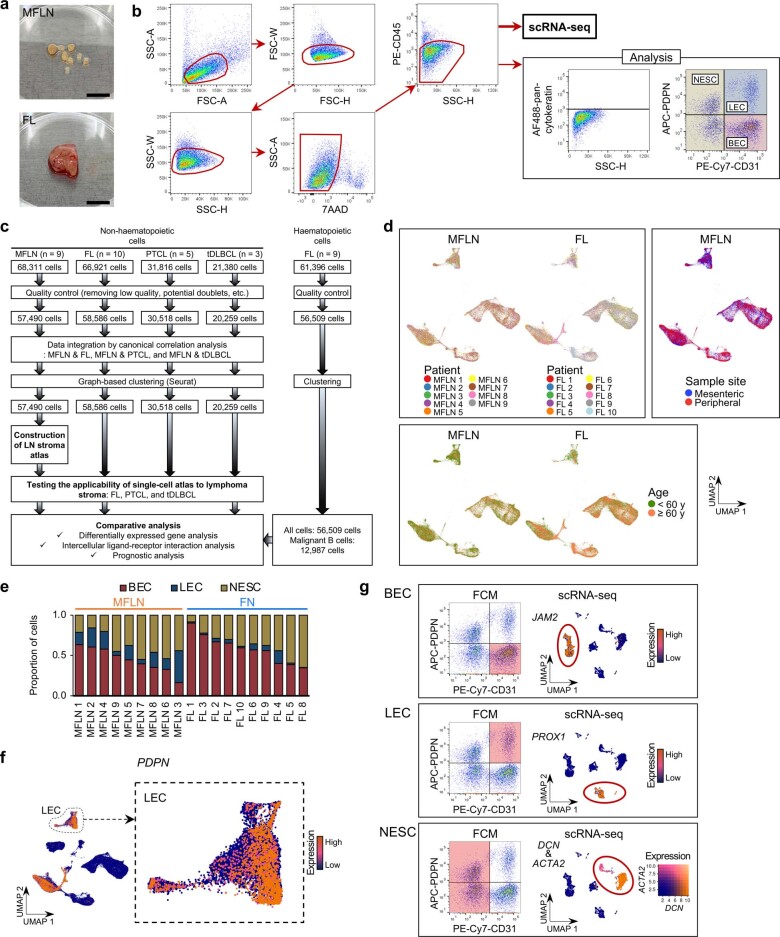
Single-cell analysis of LN and lymphoma NHCs. **a**, Macroscopy of representative human metastasis-free lymph node (MFLN) (top) and follicular lymphoma (FL) (bottom) samples. Scale bars, 1 cm. **b**, Gating strategy for the isolation and analysis of non-haematopoietic cells (NHCs) in human LN and lymphoma in flow cytometry (see ‘Single-cell isolation of LNNHCs’ in the Methods section). BEC, blood endothelial cell; LEC, lymphatic endothelial cell; NESC, non-endothelial stromal cell; scRNA-seq, single-cell RNA sequencing. **c**, Overview of scRNA-seq analysis conducted in the present study. PTCL, peripheral T-cell lymphoma; tDLBCL, diffuse large B-cell lymphoma transformed from FL. **d**, UMAP plots of NHCs, colour coded by patients (top left), sites of sample collection (top right), and patient age (bottom), according to patient cohorts. **e**, Proportion of each major NHC component between MFLN (left) and FL (right) cohorts. **f**, *PDPN* expression in stroma-enriched cells from MFLN samples. High magnification image indicates heterogeneous *PDPN* expression levels among LECs. **g**, Strategy used to detect BECs (top), LECs (middle), and NESCs (bottom) using flow cytometry (FCM, red-coloured) or scRNA-seq (red circles) analysis in a representative case (MFLN 8).

To profile NHCs in human LNs and lymphomas, we performed scRNA-seq and data integration of NHCs extracted from LN samples without tumour cell infiltration (metastasis-free LNs (MFLNs)) from nine patients with a neoplasm and nodal FL samples from ten patients (Fig. 1a, Extended Data Fig. 1a–c and Supplementary Table 1). Gene mutations identified in FL samples are presented in Supplementary Tables 1 and 2. Graph-based clustering of integrated cells led to the identification of three major NHC components (BECs, LECs and NESCs) and three contaminating haematopoietic cell components (lymphocytes, plasma cells and dendritic cells) on the uniform manifold approximation and projection (UMAP) (Fig. 1b). Cell-type annotation was performed by analysing the expression levels of canonical gene markers (Fig. 1c) and differentially expressed genes (DEGs) (Fig. 1d and Supplementary Table 3). Clustered NHCs were uniformly distributed across patients, cohorts, sample collection sites and patient age (Fig. 1b and Extended Data Fig. 1d,e). Notably, expression of the marker *PDPN*, which has been used to isolate LECs in previous scRNA-seq studies^22–24^, was either partially decreased or absent in LECs (Extended Data Fig. 1f). Accordingly, the proportion of LECs among NHCs detected by flow cytometry was slightly smaller than that determined using scRNA-seq, although we observed concordance in the proportion of each NHC component between both methods (Fig. 1e and Extended Data Fig. 1g).

To identify subclusters within each of these three major NHC components, we extracted each NHC component in silico and subjected it to re-clustering. Notably, NHCs of MFLNs and FL were similarly distributed (Fig. 1f), which is in contrast to observations in solid cancers^27,28^. Here we first sought to construct a single-cell atlas of NHCs in MFLNs.

### Ten subclusters of human LN BECs

**Fig. 2:**
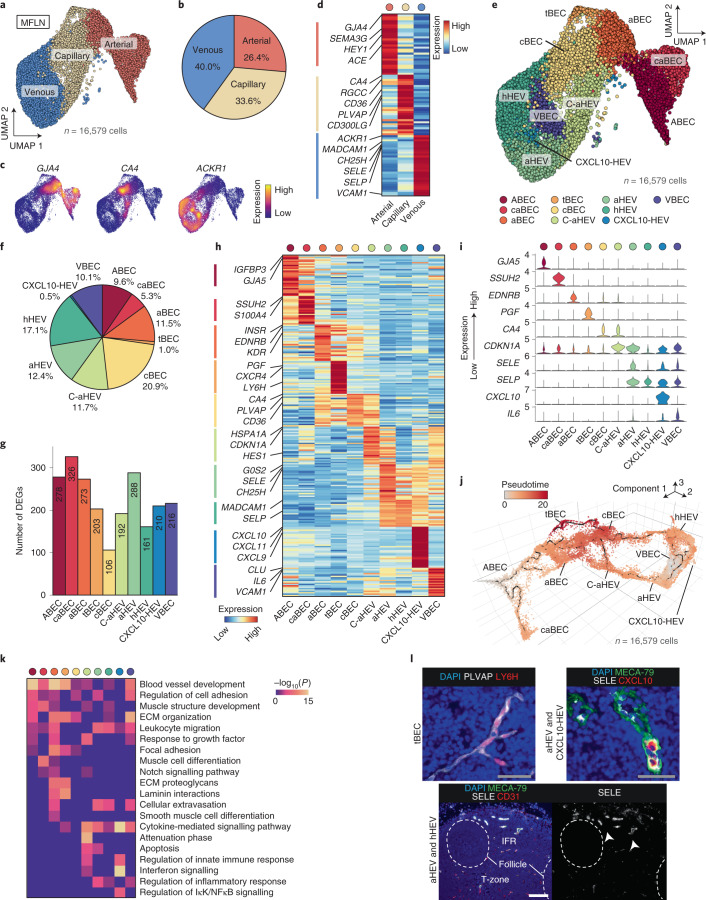
A single-cell atlas of human LN BECs. **a**, UMAP plot of MFLN BECs coloured according to classification of arterial, capillary and venous BECs. **b**, The proportions of arterial, capillary and venous BECs in MFLN samples. **c**, Expression levels of arterial, capillary and venous BEC marker genes. **d**, Heatmap showing the expression of top-ranking marker genes of arterial, capillary and venous BECs. Key genes are indicated on the left. **e**, UMAP plot of ten MFLN BEC subclusters identified by unsupervised clustering. **f**, Prevalence of each BEC subcluster in MFLN samples. **g**, Number of DEGs per BEC subcluster. **h**, Heatmap showing the expression of top-ranking marker genes for each BEC subcluster. Key genes are indicated on the left. **i**, Violin plots representing the expression of top marker genes for each BEC subcluster. **j**, Single-cell BECs ordered according to pseudotime developmental stages. Dark winding lines in the cell object indicate putative developmental trajectories. Cell regions are assigned to BEC subclusters based on subcluster-defining gene expression levels. **k**, GO enrichment analysis of DEGs for each BEC subcluster. **l**, IF staining of PLVAP (white) and LY6H (red) (top left) to identify tBECs; MECA-79 (green), SELE (white) and CXCL10 (red) (top right) to identify aHEVs and CXCL10-HEVs; and MECA-79 (green), SELE (white) and CD31 (red) (bottom) to discriminate aHEVs (arrowheads) from hHEVs. Dashed lines indicate follicles. Scale bars, 50 μm (grey) or 200 μm (white). Representative images from one of three independent experiments are shown.

We identified arterial, capillary and venous BECs (Fig. 2a). Venous BECs were most prevalent in MFLNs, followed by capillary and arterial BECs (Fig. 2b). For this annotation, we used known markers, including *GJA4* for arterial BECs, *CA4* for capillary BECs and *ACKR1* for venous BECs^29–31^ (Fig. 2c,d and Supplementary Table 4).

**Extended Data Fig. 2:**
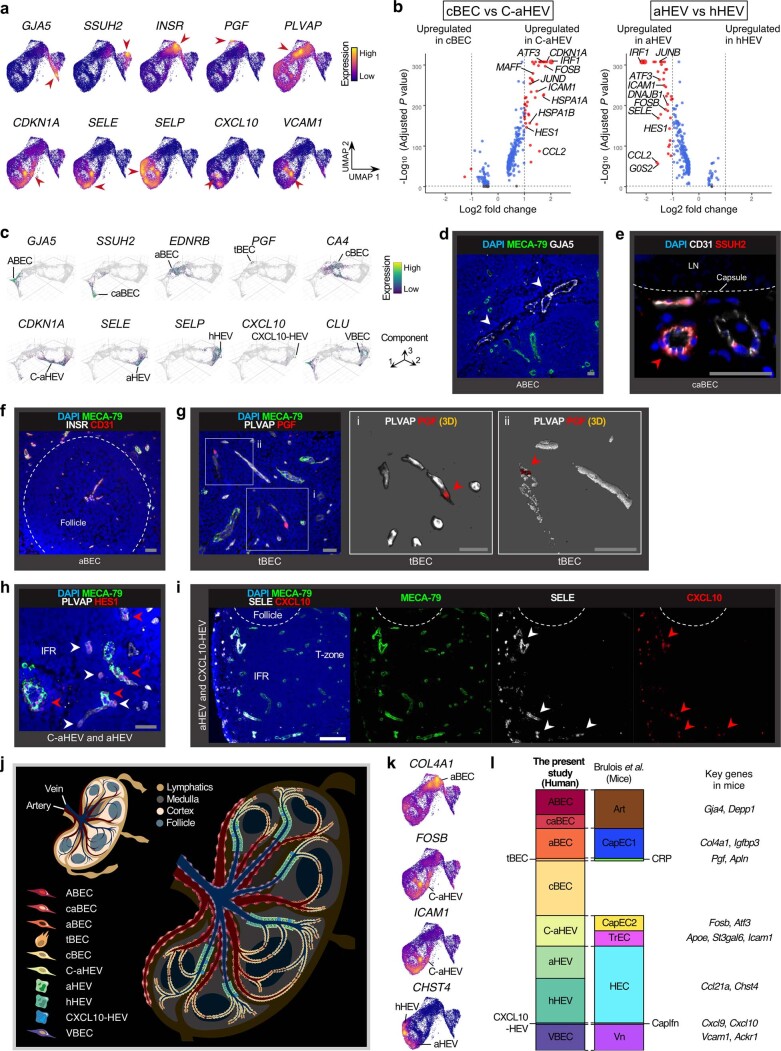
Dissection of human LN BECs at single-cell resolution. **a**, Expression of marker genes for each BEC subcluster. Red arrowheads show cells expressing indicated marker genes. **b**, Volcano plots of up- or down-regulated genes between cBECs and C-aHEVs (left) or between aHEVs and hHEVs (right). Significance was determined as an adjusted *P* value of <0.05 (two-sided Wilcoxon Rank-Sum test with Bonferroni correction) (blue-coloured dots) and log2 fold-change of ≥1 (red-coloured dots). Larger dots indicate log2 fold-change of ≥2. Key genes are labelled. **c**, Expression of marker genes for each BEC subcluster in a single-cell BEC object, generated by *Monocle 3*. **d–i**, IF staining of MECA-79 (green) and GJA5 (white) shows large arterial BECs (ABECs) (**d**, white arrowheads); CD31 (white) and SSUH2 (red) identify arteries surrounding LN capsule (caBECs) (**e**, red arrowhead); MECA-79 (green), INSR (white), and CD31 (red) identify arterioles (aBECs) (**f**); MECA-79 (green), PLVAP (white), and PGF (red). High magnification images (i and ii) are presented in 3D identifying tip cells (tBECs) (**g**, red arrowhead); MECA-79 (green), PLVAP (white), and HES1 (red) show activated HEVs (aHEVs) (red arrowheads) and transitional BECs between capillary BECs and aHEVs (C-aHEVs) (white arrowheads) (**h**); MECA-79 (green), SELE (white), and CXCL10 (red) to identify aHEVs and CXCL10-HEVs. Arrowheads show cells positive for indicated proteins (**i**). IFR, interfollicular region. Scale bars, 50 μm (grey), 200 μm (white). Representative images from one of three independent experiments are shown. **i**, LN schematic depicting topological localization of 10 BEC subclusters. **j**, Expression of marker genes for key mouse LN BEC subclusters (proposed by Brulois et al^25^) in our human data. **k**, Comparison of BEC subclusters identified here with those characterized in mice^25^. Bar heights of the mouse study are adjusted to the cell numbers (belonging to each subcluster) identified in this study. Key markers for mouse BEC subclusters^25^ are listed on right.

Unsupervised clustering of BECs further revealed ten transcriptionally distinct subclusters: large arteries (ABECs); arteries surrounding the LN capsule (caBECs); arterioles (aBECs); tip cells (tBECs); capillary BECs (cBECs); transitional BECs between capillary BECs and activated HEVs (C-aHEVs); activated HEVs (aHEVs); homeostatic HEVs (hHEVs); CXCL10^+^ HEVs (CXCL10-HEVs); and large veins (VBECs) (Fig. 2e,f). Each subcluster exhibited more than 100 DEGs that helped clearly distinguish the groups (Fig. 2g–i and Extended Data Fig. 2a).

ABECs, aBECs, tBECs, cBECs and VBECs had plausible counterparts with similar gene expression profiles in mouse tissues, including LNs^25,32^, or in other human tissues^28^ (Supplementary Note). Although mice exhibit one HEV cluster in LNs^25^, human LN HEVs were composed of three subclusters (aHEVs, hHEVs and CXCL10-HEVs). aHEVs were characterized by a prominent expression of *G0S2* (Fig. 2h, Extended Data Fig. 2b and Supplementary Table 5), which is upregulated following induction of cell-cycle progression^33^, and *SELE* (Fig. 2h,i and Extended Data Fig. 2a,b), which is upregulated by inflammation^34^. By contrast, hHEVs expressed *SELE* at low levels (Fig. 2i and Extended Data Fig. 2a,b). Notably, C-aHEVs and aHEVs both expressed stress-related genes, including those associated with heat shock proteins, NF-κB activation, JNK activation and shear stress^35^ (Extended Data Fig. 2b and Supplementary Table 6), which suggests that these subclusters respond to active cell deformation or damage.

We next performed trajectory analysis on integrated MFLN BEC data using the Monocle 3 pipeline^36^. We were able to identify all BEC subclusters in a cell object generated using Monocle 3 (Extended Data Fig. 2c). Trajectory of the arterial component flowed from ABECs to aBECs and cBECs, finally reaching tBECs (Fig. 2j). Similarly, trajectory of the venous component initially traced HEV subclusters (aHEVs and hHEVs), then proceeded to capillary subclusters (C-aHEVs and cBECs) and finally to tBECs (Fig. 2j). These findings support the idea that tBEC migration in LNs generates new capillary BECs^37^.

Gene ontology (GO) analysis revealed that factors involved in blood vessel development are enriched in ABECs, caBECs, aBECs and tBECs (Fig. 2k and Supplementary Table 7), which is in agreement with their arterial or tip cell characteristic. Leukocyte migration and cellular extravasation signatures were most enriched in aHEVs (Fig. 2k). Molecules associated with apoptosis were enriched in C-aHEVs and aHEVs (Fig. 2k). Moreover, as reported in mice^25^, CXCL10-HEVs expressed molecules associated with interferon and cytokine signalling (Fig. 2k).

Immunofluorescence (IF) analysis of BECs stained with GJA5, SSUH2 or INSR identified them as large arterial BECs in LNs (ABECs), arterial BECs outside LNs (caBECs) and arterioles (aBECs), respectively (Extended Data Fig. 2d–f). We also detected tBECs as cells stained positive for LY6H or PGF in the tips of PLVAP^+^ cBECs (Fig. 2l and Extended Data Fig. 2g). HEVs strongly expressing SELE (aHEVs) were frequently observed in interfollicular regions (IFRs) (Fig. 2l), which indicates that IFRs may serve as niches that play pivotal roles in promoting the influx of immune cells into LNs. Moreover, staining for PLVAP, HES1 and the HEV marker MECA-79 revealed that PLVAP^+^HES1^+^ capillaries (C-aHEVs) and MECA-79^+^HES1^+^ HEVs (aHEVs) (Extended Data Fig. 2b) were localized near each other (Extended Data Fig. 2h). Notably, CXCL10-HEVs were frequently observed in IFRs and were localized exclusively in the vicinity of aHEVs (Fig. 2l and Extended Data Fig. 2i). These findings, together with the GO analysis, suggest that rare CXCL10-HEVs may activate cellular trafficking of adjacent HEVs through cytokine signalling, which results in the heterogeneity of human HEVs.

In summary, our single-cell atlas of LN BECs identified three, three and four transcriptionally distinct subclusters in arterial, capillary and venous BECs, respectively, which demonstrates the unique heterogeneity of these cells in humans (Extended Data Fig. 2j–l).

### Eight subclusters of human LN LECs

A human LEC atlas^22^^[,23^ recently proposed the following six LEC subclusters: subcapsular sinus (SCS) ceiling LECs (cLECs; LEC I); SCS floor LECs (fLECs; LEC II); particular SCS cLECs that cover medullary regions (LEC III); capillary LECs in surrounding tissues (LEC IV); valve LECs (LEC V); and LECs of medullary and cortical sinuses (LEC VI).

**Fig. 3:**
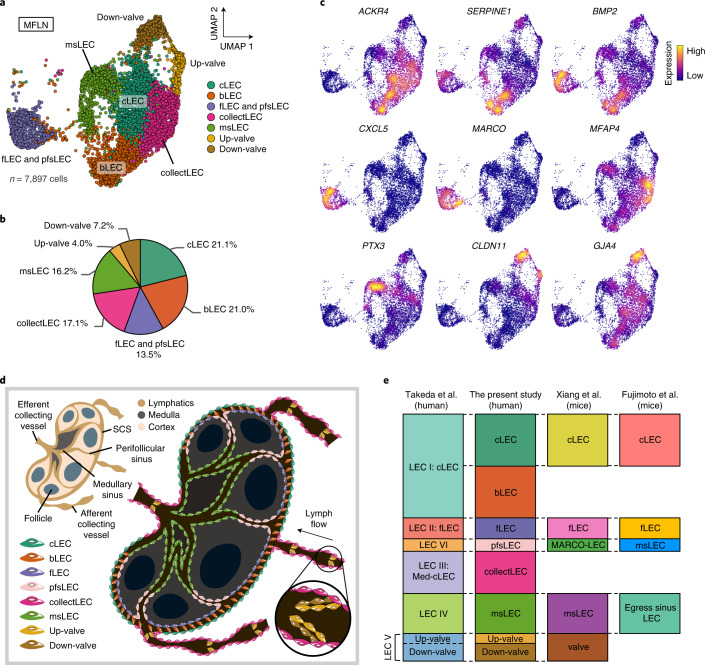
A single-cell atlas of human LN LECs. **a**, UMAP plot of MFLN LEC subclusters identified by unsupervised clustering. **b**, The prevalence of each LEC subcluster in MFLN samples. **c**, Expression levels of marker genes for each LEC subcluster. **d**, Schematic showing the topological localization of eight LEC subclusters in the LN. **e**, Comparison of subclusters identified here with those previously characterized (Takeda et al.^22^, Xiang et al.^23^ and Fujimoto et al.^24^). Bar heights of the previous studies are adjusted to cell numbers (belonging to each subcluster) identified in this study.

**Extended Data Fig. 3:**
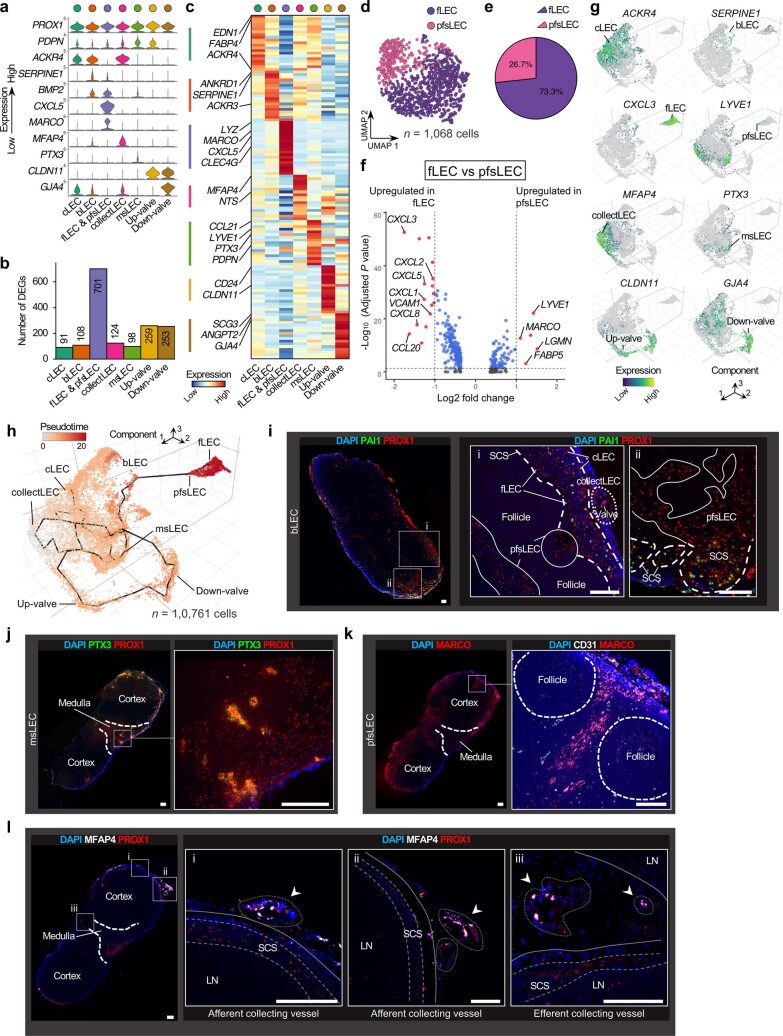
Dissection of human LN LECs at single-cell resolution. **a**, Violin plots showing expression of top marker genes for each LEC subcluster. **b**, Number of DEGs per LEC subcluster. **c**, Heatmap showing expression of top-ranking marker genes for each LEC subcluster. Key genes are indicated on the left. **d**, UMAP plot of fLECs and pfsLECs discriminated by unsupervised sub-clustering of a single ‘fLEC and pfsLEC’ subcluster. **e**, Composition of fLECs and pfsLECs in the ‘fLEC and pfsLEC’ subcluster. **f**, Volcano plot of up- or down-regulated genes in fLECs and pfsLECs. Significance was determined as an adjusted *P* value of <0.05 (two-sided Wilcoxon Rank-Sum test with Bonferroni correction) (blue-coloured dots) and log2 fold-change of ≥1 (red-coloured dots). Key genes are labelled. **g**, Expression of marker genes for each LEC subcluster in a single-cell LEC object, generated by *Monocle 3*. **h**, Single-cell LECs, ordered according to pseudo-time developmental stages. Dark winding lines indicate putative developmental trajectories. Cell regions are assigned to LEC subclusters based on marker gene expression. **i–l**, IF staining of PAI1 (green) and PROX1 (red) to identify bridge LECs (bLECs). Bold dashed lines in magnified images (i and ii) indicate subcapsular sinuses (SCSs). Solid lines indicate perifollicular sinuses. Scale bars, 500 μm (left panel), 200 μm (magnification panels) (**i**); PTX3 (green) and PROX1 (red) to identify medullary sinus LECs (msLECs). Dashed lines indicate boundaries between the LN cortex and medullary regions. High magnification image at right corresponds to boxed area at left. Scale bars, 200 μm (**j**); MARCO (red) for identification of perifollicular sinus LECs (pfsLECs). High magnification image at right shows staining of CD31 (white) and MARCO (red). Dashed lines indicate boundaries between the LN cortex and medullary regions (left) or follicles (right). Scale bars, 200 μm (**k**); MFAP4 (white) and PROX1 (red) to identify collecting vessel LECs (arrowheads). High magnification images show afferent (i and ii) or efferent (iii) collecting vessels. Bold dashed lines in left panel indicate boundaries between the LN cortex and medullary regions. Scale bars, 200 μm (**l**). Representative images from one of three independent experiments are shown.

Accordingly, we performed unsupervised clustering of MFLN LECs, DEGs and trajectory analyses, and IF staining to compare results across studies (Fig. 3a–c, Extended Data Fig. 3a–l and Supplementary Tables 8 and 9). A detailed report of our findings is included in the Supplementary Note. In brief, we initially identified seven LEC subclusters: cLECs; bridge LECs (bLECs); fLECs and perifollicular sinus LECs (pfsLECs); collecting vessel LECs (collectLECs); medullary sinus LECs (msLECs); LECs on the upstream side of valves (Up-valves); and LECs on the downstream side of valves (Down-valves) (Fig. 3a–c and Extended Data Fig. 3a–c). Additional subclustering analysis divided the single fLEC and pfsLEC subcluster into fLECs and pfsLECs^23^ (Extended Data Fig. 3d–f). Furthermore, IF staining revealed PAI1^+^, MFAP4^+^, PTX3^+^ and MARCO^+^ LECs (the latter three noted as LEC III, LEC IV and LEC VI, respectively^22^) as bLECs, collectLECs, msLECs and pfsLECs, respectively (Extended Data Fig. 3i–l). Our analysis therefore identified a total of eight LEC subclusters and unify data from recent reports^22–24^ (Fig. 3d,e).

### Twelve subclusters of human LN NESCs

**Fig. 4:**
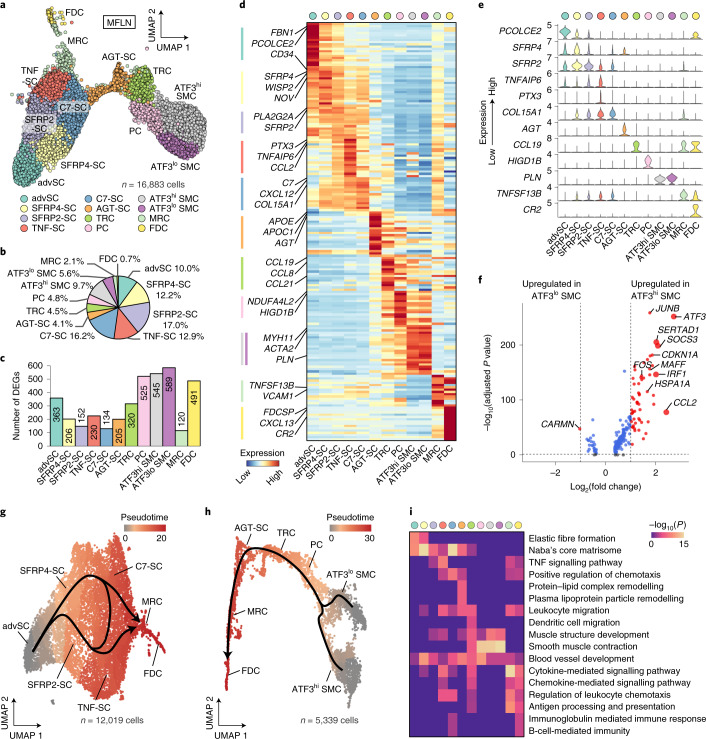
A single-cell atlas of human LN NESCs. **a**, UMAP plot of MFLN NESC subclusters identified by unsupervised clustering. **b**, The prevalence of each NESC subcluster in MFLN samples. **c**, Number of DEGs per NESC subcluster. **d**, Heatmap showing the expression of top-ranking marker genes for each NESC subcluster. Key genes are indicated on the left. **e**, Violin plots representing top marker genes for each NESC subcluster. **f**, Volcano plot of upregulated or downregulated genes between ATF3^hi^ and ATF3^lo^ SMCs. Significance was determined as an adjusted *P* < 0.05 (two-sided Wilcoxon rank-sum test with Bonferroni correction) (blue dots) and log_2_ fold-change of ≥1 (red dots). Larger dots indicate log2 fold-change of ≥2. Key genes are labelled. **g**,**h**, Pseudotime developmental stages of single cells in advSCs, SFRP4-SCs, SFRP2-SCs, TNF-SCs, C7-SCs, MRCs and FDCs (**g**) or in SMC subclusters, PCs, TRCs, AGT-SCs, MRCs and FDCs (**h**). Dark winding lines in the cell objects indicate putative developmental trajectories. Cell regions are assigned to each subcluster based on subcluster-defining gene expressions. **i**, GO enrichment analysis of DEGs for each NESC subcluster.

**Extended Data Fig. 4:**
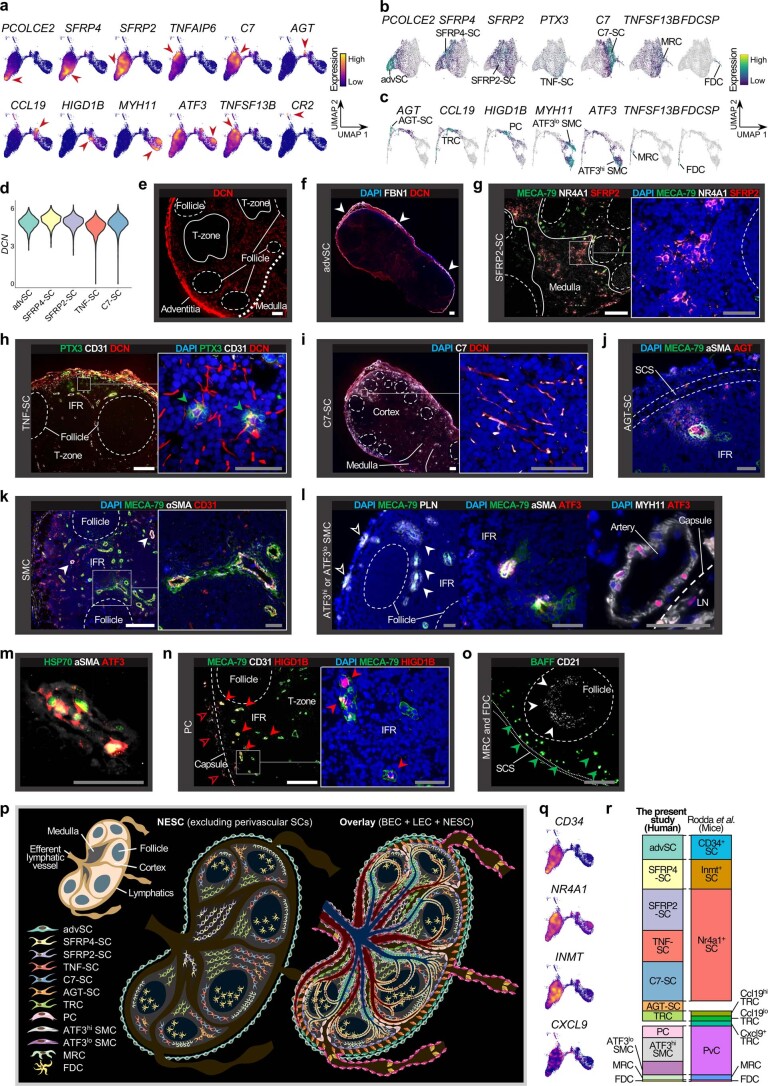
Dissection of human LN NESCs at single-cell resolution. **a**, Expression of marker genes for each NESC subcluster. Red arrowheads show cells expressing indicated marker genes. **b,c**, Marker gene expressions in a NESC object shown in Fig. 4g (**b**) or Fig. 4h (**c**). **d**, *DCN* expression in MFLN advSCs, SFRP4-SCs, SFRP2-SCs, TNF-SCs, and C7-SCs. **e–o**, IF staining of DCN showing DCN-positive fibroblasts (**e**); FBN1 (white) and DCN (red) to identify SCs in the capsule adventitia (advSC) (arrowheads) (**f**); MECA-79 (green), NR4A1 (a marker of LN fibroblastic reticular cells; white)^21^, and SFRP2 (red) to identify SFRP2-SCs (**g**); PTX3 (green), CD31 (white), and DCN (red) to identify TNF-SCs (green arrowheads) (**h**); C7 (white) and DCN (red) to identify C7-SCs (**i**); MECA-79 (green), α-smooth muscle actin (αSMA, white), and AGT (red) to identify AGT-SCs (**j**); MECA-79 (green), αSMA (white), and CD31 (red) to identify SMCs. White arrowheads indicate SMCs around arteries (**k**); MECA-79 (green) and PLN (white) identifying SMCs around HEVs (filled arrowheads) and arteries (empty arrowheads) (left); MECA-79 (green), αSMA (white), and ATF3 (red) identifying ATF3^hi^ SMCs around HEVs (middle); and MYH11 (white) and ATF3 (red) showing ATF3^hi^ and ATF3^lo^ SMCs around arteries (**l**); HSP70 (green), αSMA (white), and ATF3 (red) on SMCs (**m**); MECA-79 (green), CD31 (white), and HIGD1B (red) identifying PCs around arteries (empty arrowheads) and HEVs (filled arrowheads) (**n**); BAFF (green) and CD21 (white) identifying MRCs (green arrowheads) and FDCs (white arrowheads), respectively (**o**). IFR, interfollicular region; LN, lymph node; SCS, subcapsular sinus. Scale bars, 50 μm (grey),200 μm (white). Representative images from one of three independent experiments are shown. **p**, LN schematic depicting NESC subclusters excluding perivascular SCs (left) and an overlay image of all BEC, LEC, and NESC subclusters (right). **q**, Expression of marker genes for key mouse LN NESC subclusters^21^ in our human data. **r**, Comparison of NESC subclusters identified here with those characterized in mice^21^.

NESCs were divided into the following 12 subclusters: SCs at capsule adventitia (advSCs); SFRP4^+^ SCs (SFRP4-SCs); SFRP2^+^ SCs (SFRP2-SCs); SCs enriched for tumour necrosis factor (TNF) signalling (TNF-SCs); C7^+^ SCs (C7-SCs); AGT^+^ SCs (AGT-SCs); TRCs; pericytes (PCs); smooth muscle cells (SMCs) with high or low ATF3 expression (ATF3^hi^ or ATF3^lo^ SMCs); MRCs; and FDCs (Fig. 4a,b and Extended Data Fig. 4a). TRCs, PCs, MRCs and FDCs were annotated on the basis of conventional taxonomy^18^. The results that contributed to the annotation of these known subclusters are included in the Supplementary Note.

DEG analysis revealed that NESC subclusters exhibited more than 100 DEGs each (Fig. 4c). advSCs showed the highest *CD34* expression among NESCs (Fig. 4d) and are considered the human counterpart of mouse CD34^+^ SCs observed at adventitia of the LN capsule^21^. Both SFRP4-SCs and SFRP2-SCs shared *SFRP2* expression and were discriminated by higher *SFRP4* expression in the former (Fig. 4d,e). SFRP4-SCs also showed relatively high *INMT* expression (Supplementary Table 10), which suggests that they are the counterpart of mouse Inmt^+^ SCs observed exclusively at medullary cords^21^. TNF-SCs were specifically characterized by *PTX3* expression, and C7-SCs by abundant *C7* expression (Fig. 4d,e). AGT-SCs expressed *AGT* and high levels of the apolipoprotein genes *APOE* and *APOC1* (Fig. 4d,e). ATF3^hi^ and ATF3^lo^ SMCs both expressed muscle-specific *MYH11* and *PLN* (Fig. 4d,e), but differed in the expression of genes associated with cellular responses to stress or mechanical stimuli (Fig. 4f and Supplementary Table 11). Notably, *TNFSF13B*, which encodes B-cell-activating factor belonging to the TNF family (BAFF) and is thought to define FDCs^20^, was expressed by both MRCs and FDCs but at higher levels by MRCs (Fig. 4d,e).

Trajectory analysis revealed that MRCs were connected to TNF-SCs and C7-SCs (Fig. 4g and Extended Data Fig. 4b), which indicates that the latter two subclusters might differentiate into MRCs. Additional analysis showed a continuous trajectory from SMC subclusters to PCs, TRCs, MRCs and finally to FDCs in human LNs (Fig. 4h and Extended Data Fig. 4c), which is consistent with findings in mice of fibroblastic reticular cells in the splenic white pulp^38^.

GO analysis revealed that advSCs expressed high levels of genes involved in the formation of elastic fibres and the extracellular matrix (ECM)^39^ (Fig. 4i and Supplementary Table 12). In agreement with the preferential localization of mouse Inmt^+^ SCs at the medulla^21^, their human counterparts, SFRP4-SCs, expressed high levels of genes involved in ECM formation (Fig. 4i and Supplementary Table 12). TNF-SCs expressed genes associated with TNF signalling (*IL6* and *CCL2*) (Fig. 4i and Supplementary Tables 10 and 12), which suggests that they function in the chemotaxis of CCR2-expressing T cells, monocytes and dendritic cells to antigen sites^40^. C7-SCs expressed genes related to chemotaxis regulation (Fig. 4i), including *CXCL12* (Fig. 4d), which supports transendothelial T-cell migration across HEVs^41^. Top DEGs for AGT-SCs included *APOE*, *AGT* and *LPL*, which participate in remodelling of protein–lipid complexes and plasma lipoprotein particles (Fig. 4i and Supplementary Tables 10 and 12), which suggests that AGT-SCs may participate in lipid metabolism or transport.

IF staining was performed to identify the localization of each subcluster in LNs (Extended Data Fig. 4d–o). Fibroblasts positive for decorin (encoded by *DCN*), a strong marker of advSCs, SFRP4-SCs, SFRP2-SCs, TNF-SCs and C7-SCs (Extended Data Fig. 4d), were widely distributed in the adventitia, IFRs and medulla (Extended Data Fig. 4e). FBN1^+^ SCs (advSCs) (Fig. 4d) were observed at the capsule adventitia, as observed in mice^21^ (Extended Data Fig. 4f). SFRP2^+^ SCs (SFRP2-SCs and SFRP4-SCs) were preferentially distributed in the medulla (Extended Data Fig. 4g). PTX3^+^ SCs (TNF-SCs) were observed in IFRs (Extended Data Fig. 4h). C7-SCs were most frequent in the outer cortex, excluding follicles (Extended Data Fig. 4i), which is consistent with their proposed role in facilitating immune cell migration. AGT^+^ cells were found on outer regions of the IFRs, frequently situated between SCSs and HEVs (Extended Data Fig. 4j). SMCs were observed as α-smooth muscle actin^+^ (encoded by *ACTA2*), MYH11^+^ or PLN^+^ cells (Fig. 4d) around not only arterial BECs but also some HEVs (Extended Data Fig. 4k,l). ATF3 was positive in some SMCs around HEVs in the IFRs (aHEVs), as well as around arteries (Extended Data Fig. 4l). In line with the DEG analysis between SMC subclusters, ATF3^+^ SMCs were also marked by HSP70 (encoded by *HSPA1A*) expression (Extended Data Fig. 4m), which probably reflects cell damage induced by blood flow^42^ and/or immune cell trafficking.

To summarize, we identified 12 NESC subclusters, thereby showing unanticipated heterogeneity, linked to the distribution of other NHC subsets and LN niches (Extended Data Fig. 4p–r).

**Extended Data Fig. 5:**
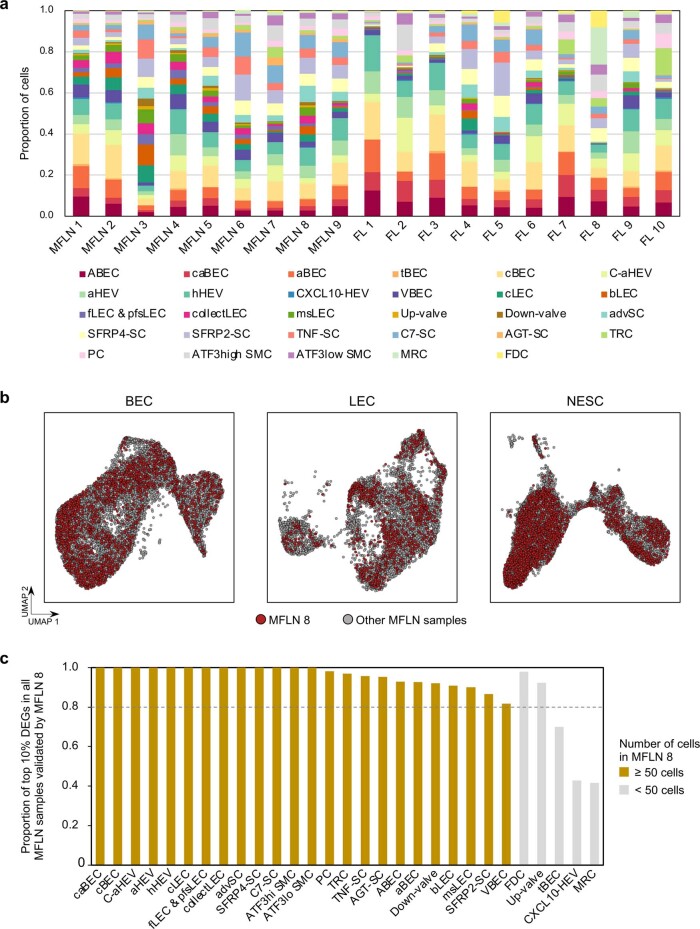
Overview of LNNHC atlas. **a**, Proportion of each NHC subcluster based on patients in the MFLN and FL cohorts. **b**, UMAP plot of major NHC components from MFLN samples, highlighting NHCs from a patient with a benign tumour (MFLN 8) (red dots). **c**, Proportions of top DEGs detected using all MFLN data and validated by DEGs in MFLN 8 according to NHC subclusters. Top DEGs were defined as the top 10% of DEGs of each NHC subcluster, calculated using all MFLN data (listed in Supplementary Table 3,8,10). Bars of subclusters with >50 cells in MFLN 8 were highlighted by ochre colouring. Dashed line indicates 80% validation.

**Extended Data Fig. 6:**
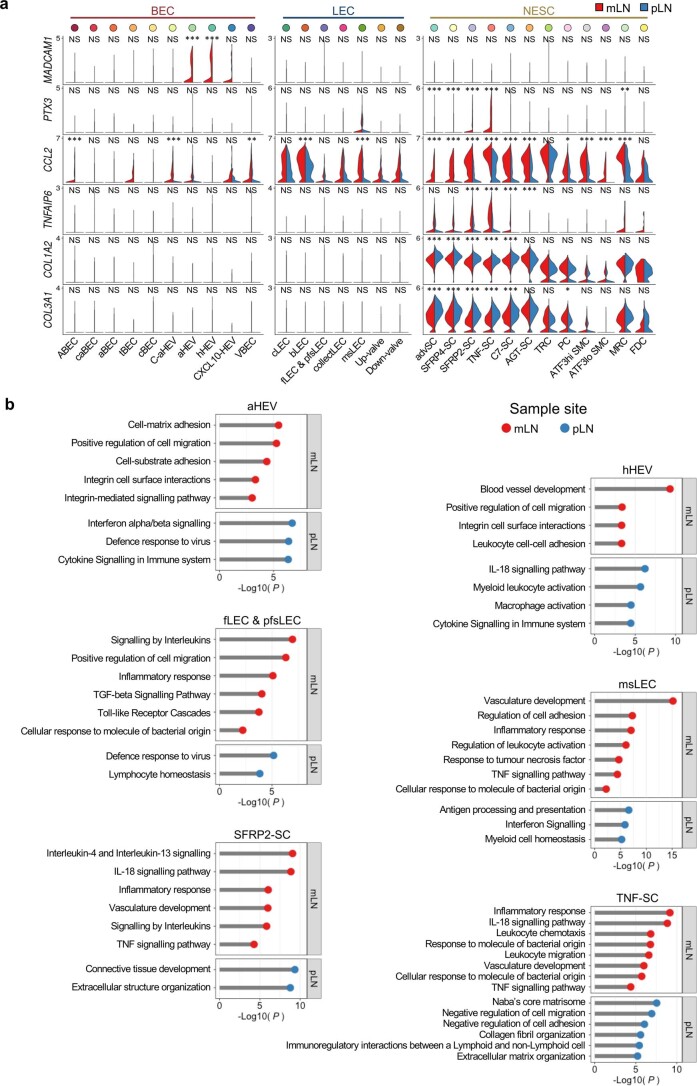
Comparative analyses between mesenteric and peripheral LNs. **a**, Violin plots comparing expressions of key genes between mLN (red) and pLN (blue) samples according to NHC subclusters. *P < 0.05, **P < 0.01, ***P < 0.001 (two-sided Wilcoxon Rank-Sum test with Bonferroni correction). NS, not significant. The exact *P* values are provided in Supplementary Table 13. **b**, Key gene ontologies of DEGs upregulated in mLN (red) or pLN (blue) compared with the other LN type according to representative NHC subclusters.

We accomplished a single-cell atlas of NHC components in human LNs (Extended Data Fig. 4p). Additional basic profiles of the atlas are described in the Supplementary Note, Extended Data Figs. 5a–c and 6a,b and Supplementary Tables 13 and 14.

### Remodelling of NHC proportions in FL

**Fig. 5:**
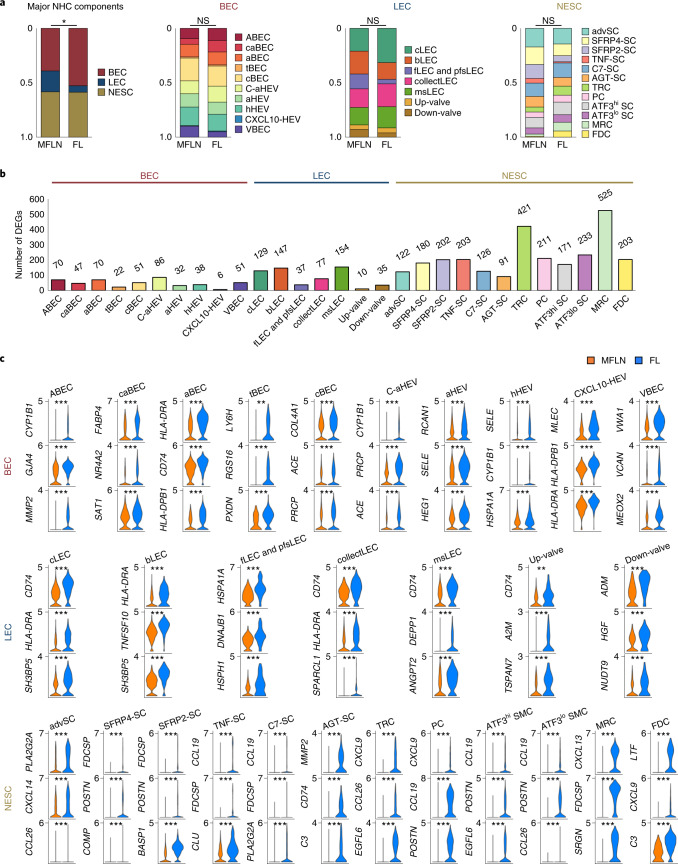
Compositional and transcriptional changes in FL stroma. **a**, Differences between MFLN and FL NHC compositions based on major NHC components, and BEC, LEC and NESC subclusters. *P = 0.010 (two-sided Chi-squared test). NS, not significant. **b**, Number of DEGs upregulated in FL NHC subclusters compared to MFLN counterparts. **c**, Violin plots of the top three DEGs upregulated in FL NHC subclusters compared to MFLN counterparts. **P < 0.01, ***P < 0.001 (two-sided Wilcoxon rank-sum test with Bonferroni correction). Exact *P* values are provided in Supplementary Tables 15–17. Statistical source data are provided. Source data

**Extended Data Fig. 7:**
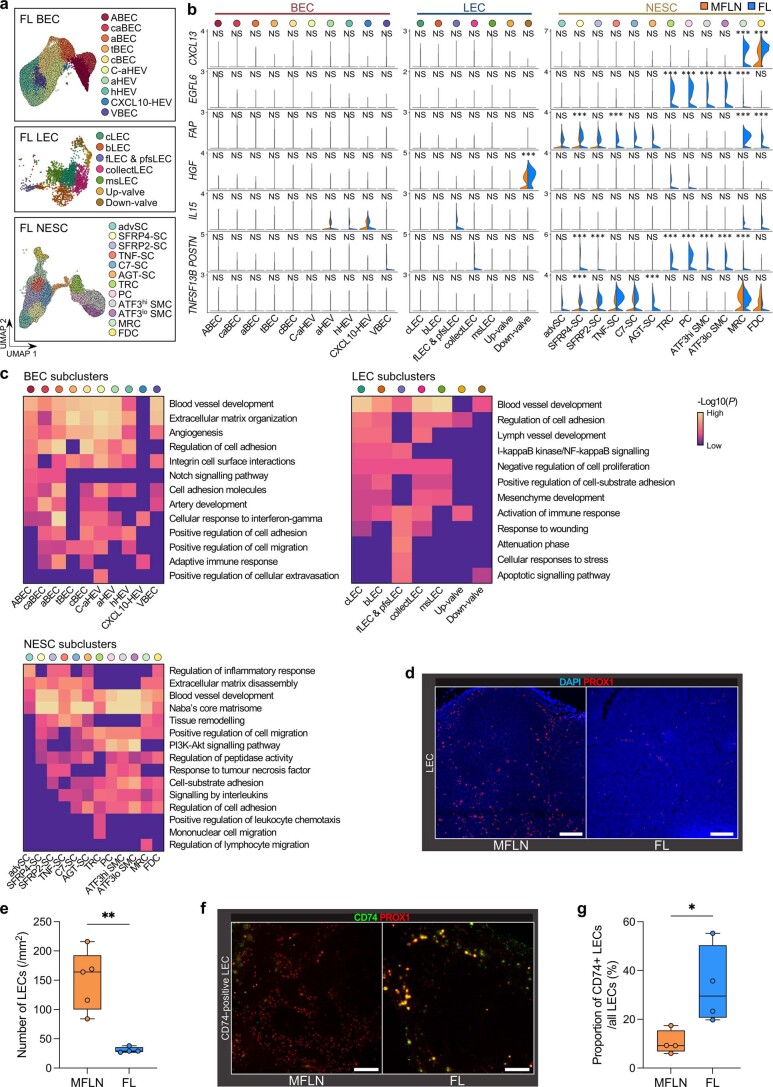
Alterations of gene expression profiles in FL stroma. **a**, UMAP plots of FL BEC (top), LEC (middle), and NESC (bottom) subclusters. **b**, Violin plots comparing expressions of key genes between MFLN (orange) and FL (blue) samples, according to NHC subclusters. ***P < 0.001 (two-sided Wilcoxon Rank-Sum test with Bonferroni correction). NS, not significant. The exact *P* values are provided in Supplementary Table 15–17. **c**, Gene ontology changes in FL NHC subclusters. GO enrichment analysis of DEGs upregulated in FL BEC (top left), LEC (top right), or NESC (bottom left) subclusters relative to MFLN counterpart subclusters. **d**, IF staining of PROX1 (red) showing LEC distribution in representative MFLN (left) and FL (right) samples. Scale bars, 200 μm. **e**, Number of PROX1-positive LECs per mm^2^, detected by IF staining in biologically independent MFLN (*n* = 5) and FL (*n* = 4) samples. The box plots show the interquartile range (box limits), median (centre line), minimum to max values (whiskers), and samples (circles). **P = 0.0025 (two-sided unpaired *t*-test). **f**, IF staining of CD74 (green) and PROX1 (red) showing CD74-positive LECs in representative MFLN (left) and FL (right) samples. Scale bars, 200 μm. **g**, Proportions of CD74-positive LECs among PROX1-positive LECs (%) detected by IF staining in biologically independent MFLN (*n* = 4) and FL (*n* = 4) samples. The box plots show the interquartile range (box limits), median (centre line), minimum to max values (whiskers), and samples (circles). *P = 0.033 (two-sided unpaired *t*-test). The statistical source data are provided. Source data

Using this atlas, we next sought to explore alterations in FL NHCs at subcluster levels by comparing them with MFLN counterparts (Fig. 5a and Extended Data Fig. 7a). Overall, the proportion of BECs were markedly increased in FL relative to MFLNs, whereas the proportion of LECs decreased (Fig. 5a). Moreover, the proportion of arterial subclusters were increased in FL BECs (Fig. 5a). In FL NESCs, the proportion of FDCs was greatly increased (Fig. 5a). Notably, MRCs were also greatly increased in FL, whereas advSCs, SFRP4-SCs, SFRP2-SCs and TNF-SCs were decreased (Fig. 5a).

### Subcluster-specific transcriptional changes in FL stroma

We next performed multistep DEG analyses in NHC subclusters of MFLNs and FL by monitoring differences in gene expression between mesenteric LNs (mLNs) and peripheral LNs (pLNs) (Extended Data Fig. 6a,b, Supplementary Tables 13 and 14 and Supplementary Note). We observed the greatest differences in MRCs, followed by TRCs, SMC subclusters, PCs and FDCs (Fig. 5b and Supplementary Tables 15–17). Figure 5c shows the expression levels of the top three DEGs upregulated in FL NHC subclusters in comparison to their MFLN counterparts. In MRCs, *CXCL13* was most markedly upregulated, and GO terms related to lymphocyte migration were enriched (Fig. 5c, Extended Data Fig. 7b,c and Supplementary Tables 17 and 18), which suggests that MRCs, in addition to FDCs, function in the accumulation of malignant B cells^13^. The expression of *TNFSF13B* was significantly enhanced in FL NESC subclusters, including SFRP4-SCs and AGT-SCs (Extended Data Fig. 7b). *IL15* and *HGF* expression levels also tended to be increased in some FL NESC subclusters, although this finding was not significant (Extended Data Fig. 7b). Notably, in some NESC subclusters, we observed marked upregulation of genes relevant to solid cancers but previously not associated with lymphomagenesis. Among them, *POSTN*, which encodes periostin (a protein secreted by cancer-associated fibroblasts (CAFs) and promotes the formation of cancer stem cell, perivascular and premetastatic niches^43^), was substantially upregulated in TRCs and PCs and in SMC subclusters of FL (Fig. 5c, Extended Data Fig. 7b and Supplementary Table 17). The expression of *EGFL6*, which encodes EGFL6 (a member of the EGF-like superfamily that reportedly promotes tumour cell growth by stimulating angiogenesis^44,45^), was highly upregulated in TRCs, SMC subclusters and MRCs (Fig. 5c, Extended Data Fig. 7b and Supplementary Table 17). CAFs positive for fibroblast activation protein (FAP) are associated with an immunosuppressive environment, which hampers immunotherapy^46–48^. Intriguingly, *FAP* was most upregulated in FSCs (MRCs and FDCs) (Extended Data Fig. 7b and Supplementary Table 17), which indicates that FSCs may create an immunological environment that favours malignant cells in FL.

In FL BECs, *GJA4* was upregulated in arterial subclusters, ABECs and aBECs (Fig. 5c and Supplementary Table 15), a pattern that is reflective of arterial vessel development^29^. Other genes involved in blood vessel development or ECM organization were upregulated in almost all subclusters (Extended Data Fig. 7c and Supplementary Tables 15 and 18). FL HEV subclusters showed high *SELE* expression (Fig. 5c and Supplementary Table 15), which is suggestive of inflammation and HEV activation^49,50^. Indeed, FL HEV subclusters expressed genes that regulate cellular adhesion and migration (Extended Data Fig. 7c and Supplementary Tables 15 and 18). Notably, expression of the tip cell markers *LY6H*, *PXDN*, *PGF* and *LOX* was markedly upregulated in FL tBECs (Fig. 5c and Supplementary Table 15), which suggests that they are involved in the acceleration of angiogenesis. The significant decrease in the proportion of LECs in FL suggests that there is widespread lymphatic damage. IF staining confirmed that the LEC density was lower in FL compared with that in MFLNs (Extended Data Fig. 7d,e). Many FL LEC subclusters also showed upregulation of heat shock genes as well as *CD74*, which reportedly functions in wound healing^51^ (Fig. 5c, Extended Data Fig. 7c and Supplementary Tables 16 and 18). CD74 overexpression was confirmed in FL LECs by IF staining (Extended Data Fig. 7f,g).

### Landscape of intercellular interactions in FL stroma

**Extended Data Fig. 8:**
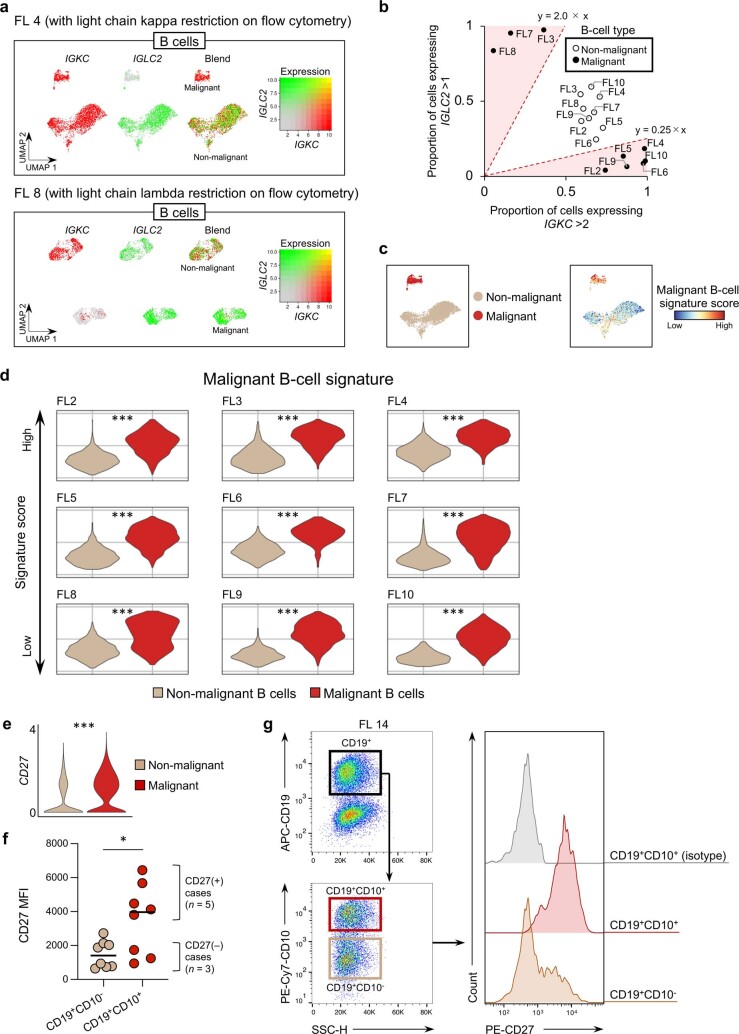
Interactome analysis across FL stroma and malignant B cells. **a**, Strategies used to identify malignant B-cell components in FL samples *in silico*. Shown are representative cases with light chain kappa (FL 4; top) or lambda (FL 8; bottom) restrictions confirmed by flow cytometry analysis (data not shown). After identifying B-cell components by detecting *CD79A* expression, we assessed expression of *IGKC* (for light chain kappa) and *IGLC2* (for light chain lambda). Clusters with cells expressing *IGKC* and those expressing *IGLC2* were considered non-malignant B cells, while clusters with cells expressing only one of these genes were considered malignant B cells. **b**, Scatter plot showing clear discrimination of malignant (filled circles) from non-malignant (empty circles) B-cell clusters in each FL sample, based on the ratio of cells expressing *IGLC2* (expression level >1; y-axis) to those expressing *IGKC* (expression level >2; x-axis,). Red-shaded areas indicate regions in which the ratio was >2.0 or <0.25. **c**, Representative UMAP plots showing B cells from FL 4 according to B-cell types (beige; non-malignant, red; malignant) (left panel) or malignant B-cell signature score (right panel). **d**, Violin plots showing malignant B-cell signature score in extracted non-malignant and malignant B cells, according to different FL samples (FL 2–10). ***P = 1.1 × 10^−204^ (FL 2), ***P = 0 (FL 3), ***P = 3.3 × 10^−176^ (FL 4), ***P = 0 (FL 5), ***P = 4.2 × 10^−122^ (FL 6), ***P = 0 (FL 7), ***P = 4.2 × 10^−161^ (FL 8), ***P = 3.7 × 10^−256^ (FL 9), ***P = 2.5 × 10^−81^ (FL 10) (two-sided Wilcoxon Rank-Sum test with Bonferroni correction). **e**, Violin plots showing the expression of *CD27* in non-malignant and malignant B cells. ***P = 0 (two-sided Wilcoxon Rank-Sum test with Bonferroni correction). **f**, Comparison of CD27 mean fluorescence intensity (MFI) between FL CD19^+^CD10^−^ (non-malignant B-cell fraction) and CD19^+^CD10^+^ (malignant B-cell fraction) cells. Circles represent biologically independent samples (*n* = 8; FL 11–18). *P = 0.039 (two-sided Wilcoxon matched-pairs signed rank test). **g**, Flow cytometry analysis of CD27 expression on CD19^+^CD10^−^ and CD19^+^CD10^+^ cells of a representative FL sample (FL 14). The statistical source data are provided. Source data

To assess the NHC–malignant B-cell crosstalk underlying FL growth, we performed scRNA-seq of cryopreserved CD45^+^ cells from nine FL samples (FL 2–FL 10) and extracted gene expression profiles of malignant B-cell clusters in silico from each (Extended Data Figs. 1c and 8a–d). We then performed intercellular ligand–receptor interaction analyses between FL NHC subclusters and malignant B cells using CellPhoneDB^52^. Thereafter, we extracted significant interactions that were considered upregulated in FL NHC subclusters relative to the corresponding MFLN subclusters.

**Fig. 6:**
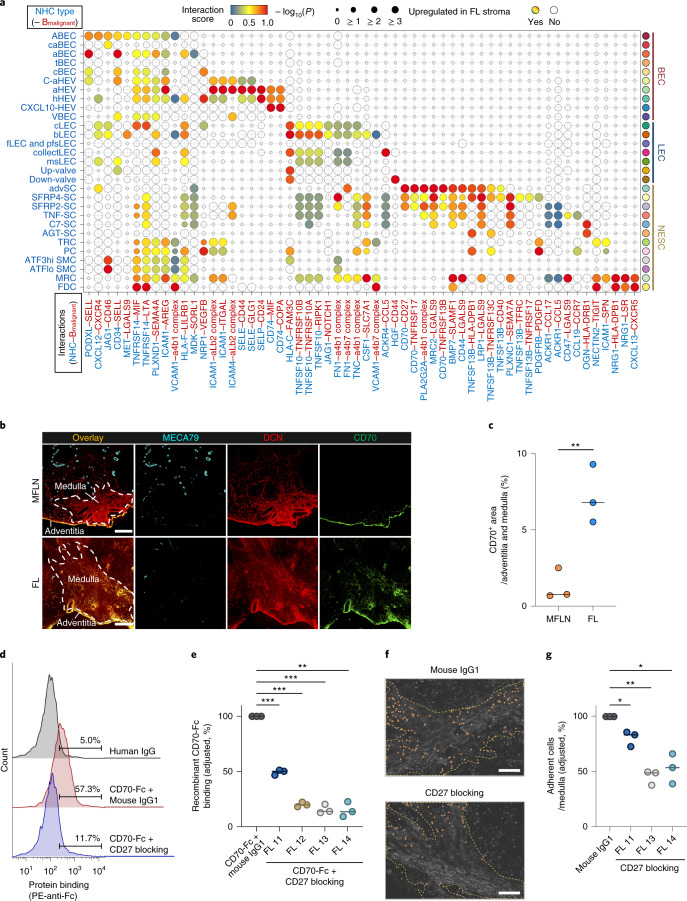
Dissection of stroma–malignant B-cell interactions in FL. **a**, Enhanced interactions across FL NHC subclusters and malignant B cells (B_malignant_). Circle size indicates the negative log_10_ of adjusted *P* values (Methods). Circles are coloured when a stroma-derived factor is upregulated in relevant FL subclusters. **b**, IF staining for MECA-79 (cyan), DCN (red) and CD70 (green) in MFLN and FL samples. Scale bars, 200 μm. Representative images from one of three independent experiments are shown. **c**, Proportions of CD70^+^ area in medullary and adventitia regions of MFLN (*n* = 3) and FL (*n* = 3) samples. Circles represent biologically independent samples. Bars indicate the median. **P = 0.0095 (two-sided unpaired *t*-test). **d**, Binding of FL CD19^+^CD10^+^ cells to CD70-Fc protein with an anti-CD27 blocking antibody or isotype human IgG. The histograms represent three independent experiments (FL 13) with the count in arbitrary units. **e**, Blocking of FL CD19^+^CD10^+^ cell binding to CD70-Fc protein after treating cells with an anti-CD27 blocking antibody (*n* = 3) or isotype mouse IgG1 (*n* = 3) in CD27^+^ FL samples (FL 11–FL 14). Proportions of cells bound to CD70-Fc protein were adjusted by subtracting nonspecific binding observed with human IgG. CD70-Fc protein binding to cells treated with isotype mouse IgG1 was set to 100% in each experiment. Circles represent independent experiments. Bars indicate the median. **P = 0.0022, ***P = 7.3 × 10^−4^ (FL 11), ***P = 2.2 × 10^−4^ (FL 12), ***P = 7.6 × 10^−4^ (FL 13) (two-sided paired *t*-test). **f**, Representative malignant B-enriched cell (FL 14) adhesion to medullary regions of FL in the presence of an isotype mouse IgG1 or anti-CD27 antibody. Orange dots indicate adherent cells. Yellow dashed lines indicate medullary regions. Scale bars, 200 μm. **g**, Blocking of malignant B-enriched cell (FL 11, FL 13 and FL 14) adhesion to FL medullary regions (per mm^2^) after treating cells with an anti-CD27 blocking antibody (*n* = 3) or isotype mouse IgG1 (*n* = 3). Adhesion of cells treated with isotype mouse IgG1 was set to 100% in each experiment. Circles represent independent experiments. Bars indicate the median. *P = 0.041 (FL 11), *P = 0.027 (FL 14), **P = 0.0050 (two-sided paired *t*-test). Statistical source data are provided. Source data

We identified a total of 58 interactions, including some previously uncharacterized in FL (Fig. 6a). In BECs, we noted that overexpression of JAG1, which is reportedly observed in B-cell lymphoma BECs and associated with aggressive lymphoma phenotypes^53^, was limited to only larger arterial BEC subclusters (ABECs and caBECs) (Fig. 6a). Interactions mediated through adhesion molecules, including the SELE–CD44 interaction^54,55^, were activated mainly in HEV subclusters (C-aHEVs, aHEVs and hHEVs) (Fig. 6a), which suggests that these HEV subclusters may contribute to the haematogenous expansion of FL cells^54,56^. Interactions that promote cancer cell death and mediated by TNFSF10 were markedly upregulated in several LEC subclusters^57^ (Fig. 6a), which suggests that LECs may antagonize lymphoma development. In NESCs, interactions associated with TNF signalling, cell adhesion, PDGF signalling and chemokine signalling were differentially activated among subclusters (Fig. 6a). Notably, overexpression of CXCL12, which reportedly supports FL cell migration, adhesion and activation^58^, was observed in advSCs (Fig. 6a). Moreover, interactions via BAFF were upregulated, even in medullary SCs (SFRP4-SCs), which suggests that stromal remodelling in FL supports the extrafollicular expansion of malignant B cells^59^. In advSCs and medullary SC subclusters, interactions mediated by stroma-derived CD70 were enhanced (Fig. 6a). Interactions mediated through PDGFRB, which promotes cell migration and angiogenesis^60^, were enhanced in TRCs and PCs (Fig. 6a), which suggests that during FL expansion, mechanisms other than CCR7–CCL19/CCL21 signalling may drive the homing of malignant B cells to the T-cell zone^61^. Instead, the CCR7–CCL19 interaction was extended to non-TRC SCs (TNF-SCs and PCs) (Fig. 6a). Consistent with the DEG analyses of MFLNs and FL, the CXCL13–CXCR5 axis^9,13^ was activated in MRCs and FDCs (Fig. 6a).

### Enhanced CD70–CD27 interaction across FL stroma

Based on the above interactome analysis results, we next sought to explore an interaction that can potentially be targeted in lymphoma. We carefully surveyed candidate interactions from the perspective of novelty in the field. We noted that the CD70–CD27 interaction in solid and haematological cancers has attracted increasing attention^62–65^, whereas interactions mediated by stroma-derived CD70 have rarely been investigated. Accordingly, we focused on the CD70–CD27 interaction for functional validation to verify the usefulness of our atlas-based analyses and to propose a potential mechanism in the stroma relevant to FL progression. Initially, we confirmed that CD70 is overexpressed in FL medullary and adventitial SCs by IF staining (Fig. 6b,c). We next examined the gene and protein expression levels of the CD70 ligand CD27 in the B cells of FL samples. Single-cell transcriptomic analysis of FL B cells showed that *CD27* was significantly upregulated in malignant B cells compared with non-malignant B cells (Extended Data Fig. 8e). Consistent with these results, flow cytometry analysis of FL haematopoietic cells showed that the CD19^+^CD10^+^ cell population (malignant B-cell enriched fraction) in 5 out of 8 (62.5%) biologically independent samples was positive for CD27, and its expression was also significantly higher in the CD19^+^CD10^+^ population than in the CD19^+^CD10^−^ population (non-malignant B-cell fraction) (Extended Data Fig. 8f,g). Among the five CD27^+^ FL samples, four (80.0%) showed unequivocal binding to recombinant human CD70-Fc protein (Fig. 6d). The binding of malignant B-enriched cells to CD70-Fc protein was significantly inhibited by the treatment of the cells with an anti-CD27 function-blocking antibody in all four cases (Fig. 6d,e). Next, we performed ex vivo cell adhesion assays using FL frozen sections and malignant B-enriched cells. The number of malignant B-enriched cells adhered to the medullary regions was significantly decreased following treatment with the anti-CD27 antibody (Fig. 6f,g).

### Prognostic implications of stroma-derived markers in FL

Next, we tested the applicability of our single-cell analysis of NHCs in the search for clinically relevant factors. To correlate niche-specific or subcluster-specific alterations in NHCs with survival of patients with FL, we utilized a bulk microarray dataset of 180 FL biopsy samples from newly diagnosed patients with available survival information^66^.

**Fig. 7:**
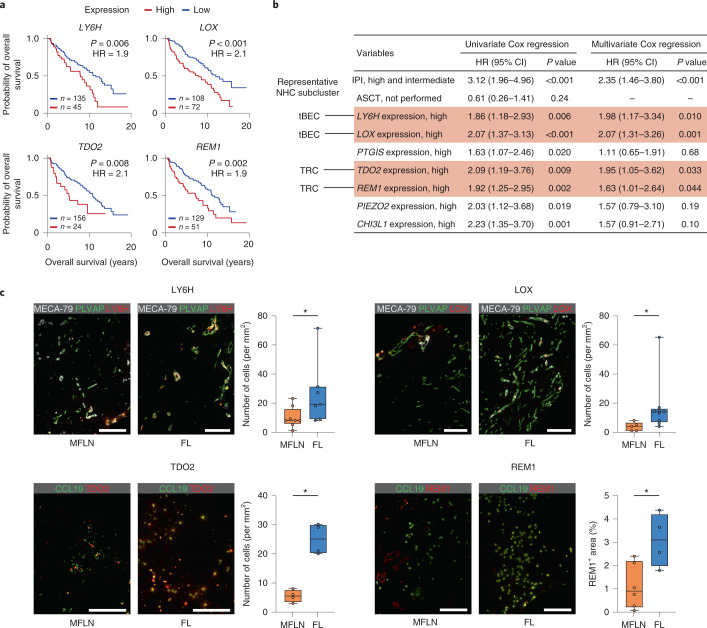
Identification of stroma-derived prognostic markers in FL. **a**, Kaplan–Meier curves showing the overall survival of patients newly diagnosed with FL (*n* = 180) based on the expression level of *LY6H*, *LOX*, *TDO2* and *REM1*. Statistical analysis was performed using the two-sided log-rank test. HR, hazard ratio. **b**, Univariate and multivariate Cox regression analyses predicting overall survival (*n* = 180). Statistical analysis was performed using two-sided Cox proportional-hazards analysis. Significant gene expression in multivariate analysis is indicated by text shaded in red. Representative NHC subcluster denotes subclusters in which indicated gene expression is most greatly upregulated in FL. CI, confidence interval. **c**, Left: images of IF staining for LY6H, LOX, TDO2 and REM1 in representative MFLN and FL samples. Scale bars, 200 μm. Right: the box plots show the interquartile range (box limits), median (centre line), minimum to maximum values (whiskers), and biologically independent samples (circles) for quantification of cell number (for LY6H, LOX and TDO2) or area (for REM1) positive for each protein in MFLN and FL samples (MFLN, *n* = 8, 5, 4 and 6; FL, *n* = 7, 9, 4 and 4 for LY6H, LOX, TDO2 and REM1, respectively). *P = 0.029 (LY6H), *P = 0.010 (LOX), *P = 0.029 (TDO2), *P = 0.038 (REM1) (two-sided Mann–Whitney *U*-test). Statistical source data are provided. Source data

**Extended Data Fig. 9:**
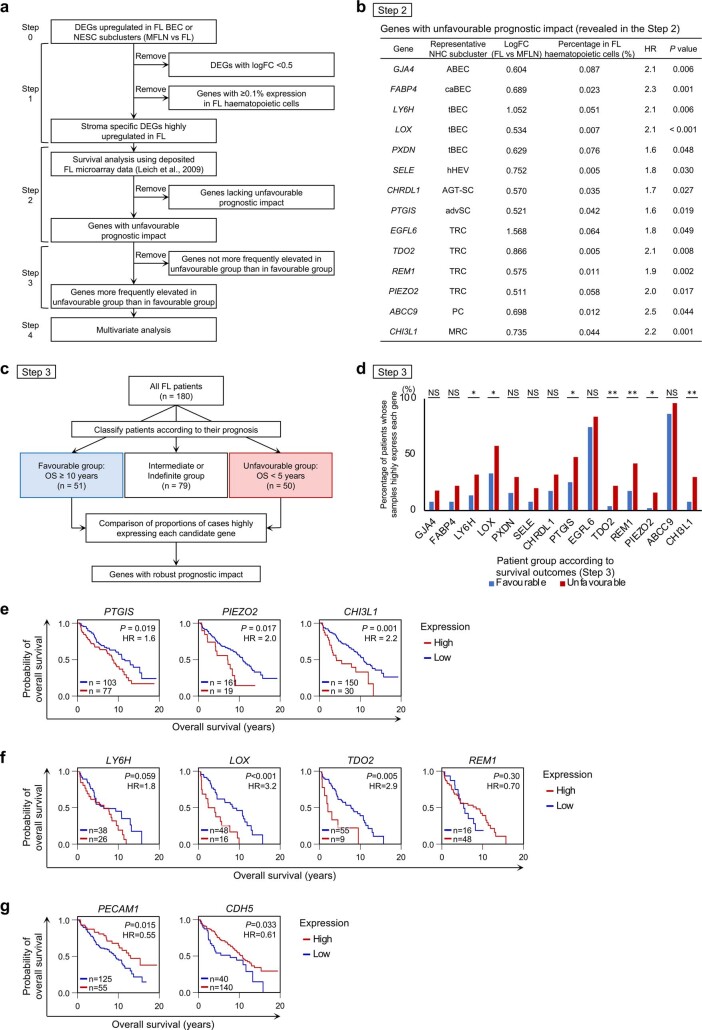
Exploring stroma-derived prognostic markers in FL. **a**, Scheme of stepwise survival analysis using public data from FL patients^66^ to identify stroma-derived prognostic markers. **b**, Genes with unfavourable prognostic impact, as revealed by survival analysis using Kaplan–Meier methods with the two-sided log-rank test (Step 2). Representative NHC subcluster denotes subclusters in which indicated gene expression is most greatly upregulated. FC, fold-change; HR, hazard ratio. **c**, Strategies used to confirm prognostic impact of candidate genes identified in Step 2 (Step 3). OS, overall survival. **d**, Results of analysis performed in Step 3. Shown is the proportion of patients whose samples highly expressed indicated genes in favourable or unfavourable prognostic groups. *P = 0.034 (*LY6H*), *P = 0.017 (*LOX*), *P = 0.024 (*PTGIS*), *P = 0.014 (*PIEZO2*), *P = 0.027 (*CHI3L1*), **P = 0.0077 (*TDO2*), **P = 0.0092 (*REM1*) (two-sided Fisher’s exact test). NS, not significant. **e**, Kaplan–Meier curves showing overall survival of newly diagnosed FL patients (n = 180) based on expression of *PTGIS*, *PIEZO2*, and *CHI3L1*. Statistical analysis was performed using the two-sided log-rank test. **f**, Estimation of overall survival based on expression of *LY6H*, *LOX*, *TDO2*, and *REM1* in the FL patients of the intermediate prognosis group (n = 64, two-sided log-rank test). **g**, Estimation of overall survival based on expression of *PECAM1* and *CDH5* (n = 180, two-sided log-rank test). The statistical source data are provided. Source data

We narrowed down multivariate analysis candidates to seven genes (*LY6H*, *LOX*, *PTGIS*, *TDO2*, *REM1*, *PIEZO2* and *CHI3L1*) expressed at minimal levels in FL haematopoietic cells but at high levels in FL BEC or NESC subclusters compared with MFLN counterparts. We hypothesized that they were probably associated with unfavourable prognosis (Fig. 7a and Extended Data Fig. 9a–e). In the multivariate analysis, increased expression of the tip cell markers *LY6H* and *LOX*, as well as *TDO2* and *REM1*, were associated with an unfavourable prognosis, even after adjustment for the international prognostic index^67^ (Fig. 7b).

For each of the four genes, we performed IF staining in MFLN and FL samples. Cells expressing LY6H, LOX, TDO2 or REM1 were increased in FL compared with those in MFLNs (Fig. 7c).

Findings from the additional prognostic analyses are described in the Supplementary Note, Extended Data Fig. 9f,g and Supplementary Table 19.

### Observation of NHC subclusters across lymphomas

**Extended Data Fig. 10:**
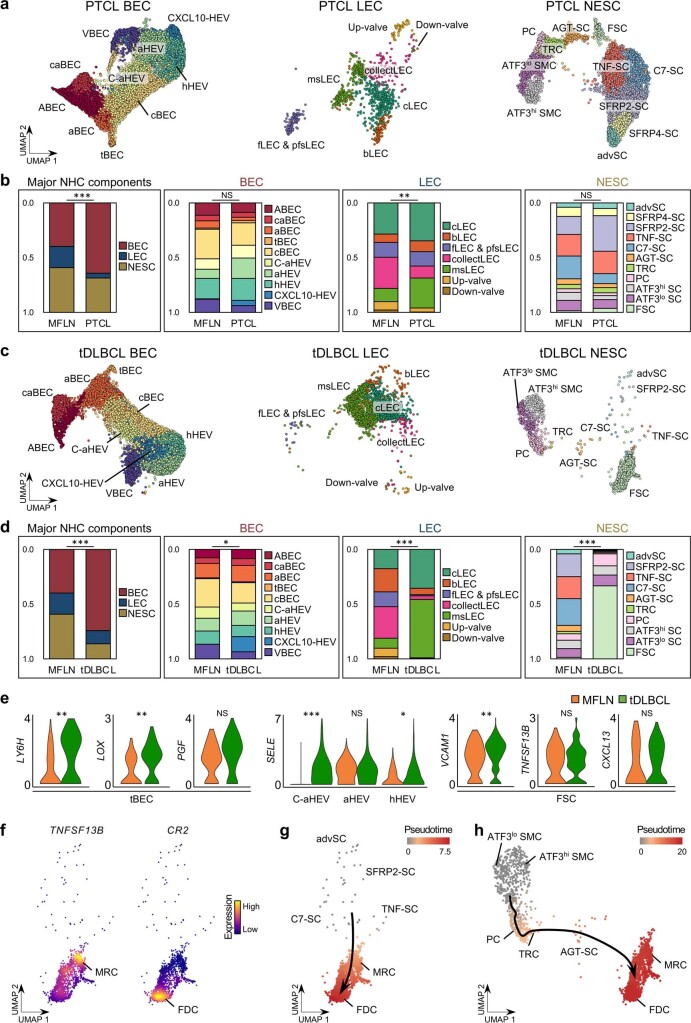
Applicability of the LNNHC atlas to PTCL and tDLBCL stroma. **a**, UMAP plots of PTCL BEC (left), LEC (middle), and NESC (right) subclusters. **b**, Compositional differences between PTCL and MFLN NHCs based on major NHC components, BEC subclusters, LEC subclusters, and NESC subclusters (from left to right). **P = 0.0076, ***P = 2.7 × 10^−4^ (two-sided chi-squared test). NS, not significant. **c**, UMAP plots of tDLBCL BEC (left), LEC (middle), and NESC (right) subclusters. **d**, Compositional differences between tDLBCL and MFLN NHCs based on major NHC components, BEC subclusters, LEC subclusters, and NESC subclusters (from left to right). *P = 0.030, ***P = 3.4 × 10^−6^ (Major NHC components), ***P = 6.3 × 10^−16^ (LEC), ***P = 1.1 × 10^−23^ (NESC) (two-sided chi-squared test). **e**, Violin plots comparing expressions of key genes between MFLN (orange) and tDLBCL (green) samples according to selected NHC subclusters. *P = 0.015, **P = 0.0039 (*LY6H*), **P = 0.0090 (*LOX*), **P = 0.0075 (*VCAM1*), ***P = 8.0 × 10^−111^ (two-sided Wilcoxon Rank-Sum test with Bonferroni correction). NS, not significant. **f**, Expression of *TNFSF13B* (left) and *CR2* (right) in tDLBCL follicular stromal cells identifying MRCs and FDCs, respectively. **g,h**, Pseudo-time developmental stages in tDLBCL advSCs, SFRP2-SCs, TNF-SCs, C7-SCs, MRCs, and FDCs (**g**) or in tDLBCL SMC subclusters, PCs, TRCs, AGT-SCs, MRCs, and FDCs (**h**). Dark winding lines in the cell objects indicate putative developmental trajectories. The statistical source data are provided. Source data

Finally, we examined whether our single-cell atlas was applicable to different lymphoma subtypes. We also investigated a more aggressive FL stromal remodelling phenotype. To this end, we performed scRNA-seq of stroma-enriched cells from five nodal peripheral T-cell lymphoma (PTCL) samples and three diffuse large B-cell lymphoma transformed from FL (tDLBCL) samples (Fig. 1a, Extended Data Fig. 1c and Supplementary Table 20). Detailed findings are described in the Supplementary Note and Extended Data Fig. 10a–h. In brief, NHC subclusters were detectable in PTCL and tDLBCL data and we observed distinct alterations in tDLBCL stroma that probably represented a terminal form of stromal remodelling in FL.

## Discussion

Here we presented a human LNNHC map at single-cell resolution that was useful for exploring changes in lymphoma NHCs.

First, we shed light on differences in mouse and human LNNHC heterogeneity. Overall, our findings suggest that human LNs harbour unique NHC subpopulations that have not been detected in murine LNs, which emphasizes the need for further human studies. As in previous studies that used fresh human LN samples^22,26^, we used MFLNs from patients with tumours to construct the atlas. Notably, a detailed analysis of LNNHCs from an individual with a benign tumour indicated that the clustering was comparable between samples from the individual with a benign tumour and patients with a malignant tumour (Supplementary Note). This observation suggests minimal or negligible influence of malignancy-derived factors on our atlas.

Second, multistep DEG analyses revealed subcluster-specific changes in FL, including those with a previously unknown function in lymphoma. We found that upregulation of some of the known intercellular interactions across FL NHCs and malignant B cells extended to unanticipated NHC subclusters and, conversely, other interactions were enhanced in limited NHC subclusters. These observations largely increase the resolution of our understating of stromal remodelling in lymphoma. Additionally, these findings may be of clinical importance, as these may be considered as potential stroma-derived prognostic factors. Notably, two tip-cell markers were upregulated in FL and could serve as prognostic factors. LOX enzymatic activity is reported to drive tumour angiogenesis by activating PDGFRβ signalling in vascular SMCs, which is consistent with our DEG analysis that FL SMCs expressed *PDGFRB* at high levels^68^. Meanwhile, our observation of *LY6H* expression in tip cells has not previously been described in mouse or human endothelial cells. We also identified *TDO2* as a prognostic predictor of FL. *TDO2* may function to attract regulatory T cells, antagonize CD8^+^ T-cell activity and accelerate myeloid cell tolerogenicity^69^. *REM1* overexpression in TRCs and PCs was also associated with unfavourable FL outcomes. Thus, further analysis of this gene, which has been scarcely explored, and its relevance to the lymphoma stroma is warranted. As the enrichment of the FL TRC signature per se was not prognostic (Supplementary Note), qualitative rather than quantitative alterations in certain NHC subpopulations may affect the chemoresistance and prognosis of FL more precisely. In addition to these prognostic factors, many upregulated genes with or without a known pro-tumorigenic function were included in our dataset, which makes our atlas a powerful discovery tool for additional therapeutic targets.

Third, we found that the CD70–CD27 interaction via stroma-derived CD70 was enhanced in FL. Although the role of CD70 has increasingly been investigated in the context of interplays across various immune cells and cancer cells^64,65^, lymphoma SCs have not been explored as a source of CD70. A recent report suggested that CD70 expressed by CAFs supports tumour progression in solid cancers by facilitating cancer cell migration^70^. Consistent with these findings, we confirmed binding between CD70 and malignant B cells that could be blocked by an antagonist against the CD70 ligand CD27. CD70 was upregulated in extrafollicular FL SCs, which suggests that CD70 may facilitate the infiltration of lymphoma cells into extrafollicular regions during tumour progression. Our analysis therefore proposes stroma-derived CD70 as a potential biomarker and therapeutic target for FL.

Last, we found that NHC heterogeneities in LNs were detectable even in aggressive lymphomas, thereby confirming the usefulness of our NHC atlas to characterize the stroma of various lymphoma subtypes. In particular, alterations in tDLBCL stroma harmonized with those in FL, thereby supporting the findings from the analysis of FL stroma. Furthermore, our findings indicate that extrafollicular SCs, including TRCs and medullary SCs, not only promote extrafollicular infiltration of FL cells but simultaneously differentiate into FSCs and are finally replaced by FSCs in more advanced phenotypes. This reflects a unique stromal transition that corresponds to the FSC-dependent growth of FL^71^.

Limitations of this study include the quantity of samples, which may not be sufficient to identify all NHC subpopulations or to precisely determine the correlation between the NHC heterogeneities in the transcriptome data and patient characteristics, such as genomic alterations. Second, we cannot completely exclude the possibility that our atlas is influenced by unknown factors from a distant malignancy. Third, our study was not designed to analyse other lymphoma subtypes or non-lymphoma diseases. Finally, further functional validation is required to confirm our findings relevant to each NHC subcluster.

In summary, our LNNHC atlas is of value to lymphoma researchers as it largely updates the NHC taxonomy in human LNs in the context of lymphoma. This study provides a platform for future research that aims to deepen our understanding of LN or lymphoma biology and to improve lymphoma management.

## Methods

### Human samples

This study was approved by the Ethics Committee of the University of Tsukuba Hospital and the review boards of associated institutions that provided human samples (Kameda Medical Center, NTT Medical Center Tokyo and Mito Medical Center) and conducted according to all relevant ethical regulations regarding human participants. Written informed consent was obtained from all participants. The participants were not compensated for taking part in the study. For scRNA-seq, MFLN samples were prospectively collected from patients with a neoplasm (*n* = 9) who had undergone surgical LN dissection between January and June 2020. Non-sentinel LNs without enlargement (<1 cm) were used. The collected LNs were verified as malignancy-free via flow cytometry analysis of pan-cytokeratin negativity (Extended Data Fig. 1b). Nodal FL (*n* = 10), PTCL (*n* = 5) and tDLBCL (*n* = 3) samples were also prospectively collected between August 2019 and May 2020. Furthermore, for functional experiments, additional nodal FL samples (*n* = 8) were collected between May 2020 and August 2021. Lymphoma diagnosis of tissue specimens was made pathologically, phenotypically and/or referring to results of cytogenetic examinations, including fluorescence in situ hybridization analysis by expert haematopathologists.

### Single-cell isolation of LNNHCs

After collection, LN or lymphoma samples were immediately minced and digested for 1 h with RPMI 1640 medium (Sigma-Aldrich, R8758) with 5% fetal bovine serum (FBS) containing 0.2 mg ml^–1^ collagenase P (Sigma-Aldrich, 11213857001), 0.8 mg ml^–1^ dispase (Gibco, 17105041) and 0.1 mg ml^–1^ DNase I (Worthington, LS002139), with continuous agitation. Cells were then filtered through a 70-μm mesh, and red blood cells were lysed in 1% ammonium-chloride-potassium buffer. Thereafter, haematopoietic cells and contaminated red blood cells were depleted using human CD45 (130-045-801) and CD235a (130-050-501) microbeads according to the manufacturer’s instructions (Miltenyi Biotec). For MFLN samples, the remaining single-cell suspension was incubated with phycoerythrin (PE)-anti-CD45 (BioLegend; 1:500) in combination with Alexa Fluor 488-pan-cytokeratin (ThermoFisher Scientific; 1:500), allophycocyanin (APC)-anti-podoplanin (BioLegend; 1:500) and PE-cyanin 7 (PE-Cy7)-anti-CD31 (BioLegend; 1:500). For lymphoma samples, PE-anti-CD45 was mixed with fluorescein isothiocyanate (FITC)-anti-CD31 (BioLegend; 1:500), APC-anti-podoplanin (BioLegend; 1:500) and PE-Cy7-anti-CD34 (BioLegend; 1:500). The samples were incubated for 20 min, then 7-AAD viability staining solution (ThermoFisher Scientific, 00-6993-50; 1:1,000) was added and incubated for 10 min in the dark on ice. CD45^–^ live cells were sorted using a FACSAria II or III (BD Bioscience) after removing doublets by gating with a FSC-H versus FCS-W plot and a SSC-H versus SSC-W plot. Flow cytometry data were analysed using FlowJo software (Tree Star, v.10.7.1). CD45^+^ cells were cryopreserved in FBS plus 10% dimethylsulfoxide in liquid nitrogen.

### Library preparation, sequencing and data pre-processing

Sorted CD45^–^ cells were converted to barcoded scRNA-seq libraries using Chromium Single Cell 3′ reagent kits (V3) (10X Genomics) according to the manufacturer’s instructions (CG000183 Rev A), aiming for 5,000–8,000 cells per library. Library quality control and quantification were performed using a KAPA Library Quantification kit for Illumina platforms (Kapa Biosystems, KK4873) and a 2100 Bioanalyzer High Sensitivity DNA kit (Agilent, 5067-4626). Libraries were sequenced on an Illumina HiSeq X Ten system with an average depth of 31,439 reads per cell, then mapped to the human genome (build GRCh38) and demultiplexed using CellRanger pipelines (10X Genomics, v.3.1.0).

### Data processing and cell clustering of individual cases

Pre-processed data from each sample were further processed and analysed individually using the R package Seurat (v.3.2.2) on RStudio (v.3.5.0 or v.4.0.2). After removing ribosomal genes, genes expressed in fewer than 3 cells and cells expressing fewer than 200 genes, we filtered out cells with fewer than 200 unique feature counts (low-quality cells). Cells with unique feature counts greater than three times the median value (possible doublets) and/or cells with more than twice the median number of mitochondrial genes (possible apoptotic or lysed cells) were also removed. We then normalized data using the NormalizeData function and extracted highly variable features using the FindVariableFeatures function. Normalized data underwent a linear transformation (scaling) and principal component analysis (PCA) based on variable features using the RunPCA function. Graph-based clustering was then performed according to gene expression profiles using the FindNeighbors and FindClusters functions with default parameters, and results were visualized using a nonlinear dimensional reduction UMAP technique running RunUMAP and DimPlot functions. Cell clusters were annotated based on the expression of canonical markers, including *PECAM1* and *JAM2* for BECs, *PECAM1* and *PROX1* for LECs, *ACTA2* for SMCs, *CCL19* and *CCL21* for TRCs, *CR2* for FDCs, *DCN* for other NESCs, *PTPRC* for contaminating lymphocytes, *SDC1* for plasma cells, and *CCR7* and *CD83* (in cells weakly *PTPRC*^+^) for dendritic cells^22,72,73^. *MKI67* and *TOP2A* expression levels were used to identify clusters of an aggressively proliferative nature. For the FL sample 3, which came from a patient with intra-submandibular gland FL, any distinct clusters negative for all canonical markers and positive for keratin genes (indicating glandular tissue contamination) were removed. All other cases were confirmed to consist solely of these major clusters. We confirmed a negligible presence of ambient RNA contamination in single-cell NHC data, and found an imperceptible influence of potential RNA contamination on clustering results in all LN and lymphoma samples by using the DecontX (in the celda package, v.1.6.1) and SoupX (v.1.5.2) packages (data not shown)^74,75^.

### Data integration with batch effect collection

We performed canonical correlation analysis^76^ to identify shared sources of variation across multiple datasets using the FindIntegrationAnchors function and integrated them using anchors from the IntegrateData function with canonical correlation dimensions of 20. Integrated data were scaled and underwent PCA as performed in individual datasets.

### Supervised annotation and unsupervised clustering of LNNHCs

We performed graph-based clustering of PCA-reduced integrated data and supervised annotation, as described in ‘Data processing and cell clustering of individual cases’ above. Clusters characterized by extremely low unique feature counts (low-quality cells) were removed.

Next, we extracted the three major NHC components (BECs, LECs and NESCs) in silico and performed scaling, PCA-based dimensional reduction and unsupervised graph-based subclustering of each component. We removed subclusters that were considered possible doublets as characterized by high expressions of marker genes for different NHC components and incongruously high unique feature counts. In BEC subclustering, we also performed supervised annotation for the identification of arterial, capillary and venous BECs using canonical markers for each BEC component^29–32^.

### DEG analysis

DEG analysis was performed using the FindMarkers or FindAllMarkers functions with a minimum of 20% of the gene-expressing cells and a minimum log fold-change of 0.25 in gene expression between each cluster and other clusters. We primarily used the Wilcoxon rank-sum test for DEG detection. To confirm detected DEGs, we also used the model-based analysis of single-cell transcriptomics (MAST) method^77^. DEGs were defined as genes confirmed to show an adjusted *P* value (based on the Bonferroni correction) of <0.05 by using both methods. Results of the Wilcoxon rank-sum test were used to construct DEG lists and volcano plots. Volcano plots were created using the R package EnhancedVolcano (v.1.8.0). DEG analysis to compare corresponding clusters between mLN and pLN samples and between MFLN and FL samples was performed in a similar manner using the cut-off parameters described above.

For DEG analyses between MFLN and FL NHC subclusters, we adopted a multistep approach. Several previous studies had indicated differences in gene and protein expression between mLNs and pLNs^78–81^. Therefore, we initially profiled DEGs between mLNs and pLNs among MFLNs at subcluster levels (Supplementary Table 13 and Supplementary Note). Referring to this profile, we identified DEGs upregulated in FL by removing those detected between mLNs and pLNs. We also performed DEG analysis between MFLN and FL NHC subclusters using only pLN samples (MFLN 7–MFLN 9 and FL 2–FL 10) to support the reliability of the detected DEGs.

GO enrichment analysis of DEGs in particular clusters was performed using Metascape (http://metascape.org)^82^.

### Trajectory analysis

We performed trajectory analysis using the Monocle 3 package (v.0.2.3)^36^ in RStudio on integrated BEC, NESC and LEC data constructed using Seurat. Data pre-processing was performed using the preprocess_cds function, with the number of dimensions set at 100. Dimensionality reduction and clustering were performed using the reduce_dimension and cluster_cells functions, respectively. We then fit a principal graph within each cluster using the learn_graph function and visualized the order of cells in pseudotime by plot_cells or plot_cells_3d functions, as appropriate with the pseudotime colouring option.

### Single-cell analysis of FL haematopoietic cells

We performed single-cell analysis of cryopreserved CD45^+^ cells from nine FL samples (FL 2–FL 10). After thawing, cell suspensions were filtered through a 70-μm mesh and incubated with 7-AAD viability staining solution for 10 min in the dark. The 7-AAD^–^ live cells were sorted using a FACSAria II or III after removing doublets, then were converted to barcoded scRNA-seq libraries, as performed for CD45^–^ cells. Library preparation, sequencing and data processing were performed as for CD45^–^ cells. Data quality control, processing and graph-based clustering were performed in each individual case using the Seurat package, with dimension and resolution parameters of 50 and 0.5, respectively. Thereafter, we identified malignant B-cell populations by detecting restrictions of light chain kappa/lambda genes, as suggested by previous studies^83,84^. In brief, we projected the B-cell marker *CD79A* and the light chain genes *IGKC* (for light chain kappa) and *IGLC2* (for light chain lambda) to cell clusters on the UMAP plot of each sample (Extended Data Fig. 8a). We then calculated the ratio of cells expressing *IGLC2* and *IGKC* with expression levels of >1 and >2, respectively, in each B-cell cluster. We defined B-cell clusters with a ratio of >2.0 or <0.25 as malignant (Extended Data Fig. 8b).

### Malignant B-cell signature analysis in FL B cells

To support the reliability of malignant B-cell detection, we performed signature analysis on data from FL B cells. We developed a gene set that represents a malignant B-cell signature based on the recent single-cell analysis of FL B cells reported by Andor et al.^83^. We carefully selected genes that were described as significantly upregulated in malignant compared to non-malignant B cells in a uniform manner among different FL samples^83^. Selected genes are listed in Supplementary Table 21. A malignant B-cell signature score was calculated in B cells of all nine FL samples using the GSVA package (v.1.38.2)^85^ and depicted using the FeaturePlot and VlnPlot functions of Seurat.

### Intercellular ligand–receptor interaction analysis

We investigated interactions between NHC subclusters and malignant B cells of nine FL samples (FL 2–FL 10) using the CellPhoneDB package (v.2.1.1)^52^ on Python (v.3.6). Gene expression information relevant to each NHC subcluster in integrated FL NHCs was used for NHC data, whereas gene expression information relevant to malignant B-cell clusters in each FL sample was separately used for malignant B-cell data, as gene expression profiles of malignant B cells vary greatly among samples. We then performed pairwise comparisons between NHC subclusters and malignant B-cell clusters. In brief, we derived potential ligand–receptor interactions based on the expression of a receptor gene by one lineage subpopulation and a ligand gene by another. We filtered genes expressed in >20% of cells in any given subpopulation. We then permuted the cluster labels of all input cells 1,000 times and calculated the mean interaction score (the average receptor expression level in a subpopulation multiplied by the average ligand expression level in the interacting subpopulation), which generated a null distribution of the mean interaction score for each ligand–receptor pair in each pairwise comparison across subpopulations. Thereafter, we located observed mean interaction scores that were the same or higher than the actual mean score in the null distribution and calculated the proportion of the observed scores, conferring a *P* value for the likelihood of specificity of a given ligand–receptor complex to a given cluster pair. To consider interactions between FL NHCs and FL malignant B cells, we selected only interactions with a *P* value of <0.05 in more than half of FL cases (>4 cases). Furthermore, to assess subcluster-specific lymphomagenesis mechanisms in FL stroma, we extracted interactions that included a molecule in which gene expression was significantly upregulated in at least one FL NHC subcluster compared with that in the corresponding MFLN subcluster. We integrated interaction scores and *P* values of interactions between pairs consisting of the same NHC subcluster and malignant B-cell clusters from different FL samples, as previously described^84^. In brief, we calculated mean interaction scores for pairs that included the same NHC subcluster and malignant B-cell clusters from different FL samples, then normalized the mean interaction scores per interaction. We also combined *P* values of interactions for pairs that consisted of the same NHC subcluster and malignant B-cell clusters from different FL samples using Fisher’s method. The *P* values were corrected using the Benjamini–Hochberg method. In Fig. 6a, circles are coloured when gene expression for the indicated stroma-derived factor is upregulated in relevant FL subclusters compared to that in the MFLN counterparts (log fold-change >0 and adjusted *P* value <0.05).

### IF staining

Human LN and lymphoma samples were immediately embedded in OCT compound (Sakura Finetek Japan, 45833) and frozen in hexane cooled with dry ice. Samples were sliced to 3-μm thickness with a cryostat at −20 °C. Sections were dried for 1 h at 20 °C, fixed for 10 min in 4% paraformaldehyde, incubated for 10 min with 0.1% Triton X-100 (Sigma-Aldrich, T9284) for permeabilization, and then treated with 10% goat serum (Sigma-Aldrich, G9023) in PBS or serum-free protein blocking buffer (Dako, X0909) (when using non-goat-derived secondary antibodies) for 30 min. Sections were stained overnight at 4 °C with primary antibodies listed in Supplementary Table 22. After several washes with tris-buffered saline with tween 20 (Sigma-Aldrich, P9416), sections were stained for 1 h with combinations of the following secondary antibodies at 20 °C: AF488-goat-anti-rat IgG (ThermoFisher Scientific), AF594-goat-anti-rabbit IgG (ThermoFisher Scientific), AF594-donkey-anti-goat IgG (ThermoFisher Scientific) and AF647-goat-anti-mouse IgG (ThermoFisher Scientific). A TrueVIEW Autofluorescence Quenching kit (Vector, SP-8500) was used to decrease possible tissue autofluorescence per the manufacturer’s instructions. Sections were then mounted in mounting medium with 4,6-diamidino-2-phenylindole (DAPI; Vector, H-1200). Stained samples were imaged using a Leica DMi8 S Platform with the Thunder imaging system (3D Live Cell & 3D Cell Culture & 3D assay). Analysed LNs were verified as malignancy-free by pan-cytokeratin staining. Quantitative analysis of acquired images was performed using ImageJ software (National Institute of Health, v.2.1.0). As LNs and FL carry localized structures, we randomly acquired at least five different regions of interest within each sample and used the median values for statistical analysis.

### Flow cytometry analysis of FL haematopoietic cells

To analyse the expression of CD27 in malignant FL B cells and to perform the binding/adhesion assays described below, we used additionally collected cryopreserved FL samples (FL 11–FL 18). Clinical characteristics of patients in the additional FL cohort are described in Supplementary Table 23. After thawing, cells were filtered through a 70-μm mesh and incubated with PE-anti-CD27 (BioLegend; 1:500), FITC-anti-CD3 (BioLegend; 1:500), APC-anti-CD19 (Miltenyi Biotec; 1:500) and PE-Cy7-anti-CD10 (BioLegend; 1:500) antibodies for 20 min on ice. Cells were then incubated with 7-AAD viability staining solution for 10 min in the dark and analysed using a FACSAria II or III and FlowJo software.

### Recombinant protein binding assay

Recombinant Fc chimera CD70 (SinoBiological, 10780-H01H) or human IgG (R&D systems, 1-001-A) was incubated with a single-cell suspension of FL haematopoietic cells for 10 min at 4 °C in RPMI with 10% FCS. To block CD70–CD27 binding, cells were incubated in the presence of anti-CD27 blocking antibody (R&D systems, MAB382) or isotype mouse IgG1 (R&D systems, MAB002) for 30 min at 4 °C before binding. After binding, the cells were washed, fixed using 4% paraformaldehyde for 10 min at 20 °C, incubated with PE-anti-human IgG Fc (R&D systems; 1:500), FITC-anti-CD3 (1:500), APC-anti-CD19 (1:500) and PE-Cy7-anti-CD10 (1:500) for 20 min at 4 °C, and analysed using flow cytometry (FACSAria II or III) and FlowJo software.

### Ex vivo cell adhesion assay

Frozen FL sections were sliced at 6-μm thickness immediately before the assay. For malignant B-cell isolation, we used FL samples in which >90% B cells were confirmed to be malignant by flow cytometry. B cells were isolated from the FL haematopoietic cell suspension using an EasySep Release Human CD19 Positive Selection kit (StemCell Technologies, ST-17754). Cells were then treated with anti-CD27 blocking antibody or isotype mouse IgG1 for 30 min at 4 °C. Thereafter, 2 × 10^6^ cells were applied on the sections and incubated with 60 r.p.m. rotation for 5 min, followed by incubation without rotation for 15 min. The incubation with and without rotation was repeated two more times. After incubation, the sections were gently washed with PBS, sealed with a cover glass and imaged using a Keyence BZ-X710 microscope (Keyence). Adherent cells were manually counted using ImageJ.

### Prognostic analysis of stroma-derived markers in FL

To analyse the prognostic potential of gene expression patterns of NHCs in patients with FL, we used a bulk microarray dataset of 180 FL biopsy samples from independent, newly diagnosed cases^66^. To narrow candidates to stroma-specific genes, we initially selected DEGs upregulated in FL BEC and NESC subclusters relative to MFLN counterparts. These were narrowed down to those showing a log fold-change of >0.5 and < 0.1% of cells with an expression level higher than 0 in FL haematopoietic cells (Extended Data Fig. 9a). We did not use genes upregulated in FL LEC subclusters, as the proportion of FL LECs was considerably decreased relative to MFLN LECs and the specificity of these genes to FL stroma was considered unlikely in analyses of bulk tissues. Next, we tested the expression of all candidate genes using the Kaplan–Meier method and two-sided log-rank test. Cut-off expression values of each gene for the Kaplan–Meier survival curves was determined using maximally selected rank statistics^86^. As many putative stroma-specific genes were upregulated in FL, it was possible that *P* value collection (for example, the Bonferroni method) greatly reduced the number of candidate genes, considering that the sample size in the dataset was not particularly large. Therefore, we extracted genes with reliable prognostic impacts using another approach (Extended Data Fig. 9a,c). We initially divided patients into three groups according to survival outcomes: a favourable group, which comprised patients alive 10 years after diagnosis; an unfavourable group, which comprised patients who died within 5 years of diagnosis; and an intermediate/indefinite group, which comprised the remaining patients (Extended Data Fig. 9c). We then compared the proportion of patients with higher expression of each candidate gene between favourable and unfavourable groups. Genes were considered prognostic when the proportion was significantly higher in the unfavourable group compared with that in the favourable group (Extended Data Fig. 9d). These prognostic genes were further subjected to multivariate analysis (Extended Data Fig. 9a).

To evaluate the prognostic efficiency of the FL TRC signature, we extracted the DEGs that were upregulated in FL TRCs in comparison to MFLN TRCs (Supplementary Table 17). We considered the DEGs with an expression level higher than 0 in <0.1% FL haematopoietic cells, <10% FL BECs and <10% FL LECs and were detectable in the microarray dataset^66^. A total of 11 extracted genes constituting the FL TRC signature are listed in Supplementary Table 19.

### Whole-exome sequencing

Whole-exome sequencing was performed on genomic DNA extracted from nine FL samples (FL 2–FL 10). Libraries were prepared using SureSelect Human All Exon v.7 kits (Agilent Technologies, 5191-4004) according to the manufacturer’s instructions and sequenced using an Illumina HiSeq X Ten system with a 150-bp paired-end protocol. We used the Genomon2 pipeline (v.2.6.2) for alignment of sequence and mutation calling. Somatic mutations with a Fisher’s exact *P* value of <0.01 and an empirical Bayesian call *P* value of <0.0001 were adopted. Thereafter, mutations of synonymous single nucleotide variants, variants only in unidirectional reads, variants in intergenic, intronic, untranslated regions and noncoding RNA regions, and variants in repetitive genomic regions were excluded. Furthermore, known genetic alterations affecting at least 10% of FL^11^ were screened for additional mutations. Finally, mutations derived from mapping errors were excluded using Integrative Genomics Viewer. Detected somatic mutations are listed in Supplementary Table 2.

### Statistics and reproducibility

Statistical analysis was performed using R on RStudio or GraphPad Prism 9 (GraphPad, v.9.2.0). A two-sided *P* value of <0.05 was considered statistically significant.

### Reporting Summary

Further information on research design is available in the Nature Research Reporting Summary linked to this article.

## Online content

Any methods, additional references, Nature Research reporting summaries, source data, extended data, supplementary information, acknowledgements, peer review information; details of author contributions and competing interests; and statements of data and code availability are available at 10.1038/s41556-022-00866-3.

## Supplementary information

Supplementary Information Supplementary Note.

Reporting Summary

Peer Review Information

Supplementary Tables Supplementary Tables 1–23.

## Data Availability

The scRNA-seq data that support the findings of this study have been deposited at the European Genome-Phenome Archive (https://ega-archive.org) database and can be retrieved using the accession number EGAD00001008311. For survival analysis, a DNA microarray dataset from Leich et al.^66^ was downloaded from the Gene Expression Omnibus (GEO) (accession number: GSE16131). For mapping of scRNA-seq data, GRCh38 (https://www.ncbi.nlm.nih.gov/assembly/GCF_000001405.39) was used. All other data are available from the corresponding authors on reasonable request. Source data are provided with this paper.

## Source data

Source Data Fig. 1 Statistical source data.
Source Data Fig. 5 Statistical source data.
Source Data Fig. 6 Statistical source data.
Source Data Fig. 7 Statistical source data.
Source Data Extended Data Fig. 7 Statistical source data.
Source Data Extended Data Fig. 8 Statistical source data.
Source Data Extended Data Fig. 9 Statistical source data.
Source Data Extended Data Fig. 10 Statistical source data.

## Code availability

The codes for key computational analyses are available on GitHub at https://github.com/yoshiakiabe1018/Stroma01. All of the packages used are available online.
