# Supplementary material for: A single-cell atlas of non-haematopoietic cells in human lymph nodes and lymphoma reveals a landscape of stromal remodelling
Source: Nat Cell Biol. 2022 Mar 24;24(4):565–78. doi: 10.1038/s41556-022-00866-3 (PMC9033586; doi:10.1038/s41556-022-00866-3)
Supplement: Supplementary file 1 — Supplementary Note. [file 41556_2022_866_MOESM1_ESM.pdf]

nature portfolio

https://doi.org/10.1038/s41556-022-00866-3

# Supplementary information

# A single-cell atlas of non-haematopoietic cells in human lymph nodes and lymphoma reveals a landscape of stromal remodelling

In the format provided by the authors and unedited

## Supplementary Notes

# A single-cell atlas of non-haematopoietic cells in human lymph nodes and lymphoma reveals a landscape of stromal remodelling

Yoshiaki Abe[1], Mamiko Sakata-Yanagimoto[2,3,4]*, Manabu Fujisawa[2], Hiroaki Miyoshi[5], Yasuhito Suehara[3], Keiichiro Hattori[3], Manabu Kusakabe[2,3], Tatsuhiro Sakamoto[2,3], Hidekazu Nishikii[2,3], Tran B. Nguyen[2], Yohei Owada[6], Tsuyoshi Enomoto[6], Aya Sawa[7], Hiroko Bando[8], Chikashi Yoshida[9], Rikako Tabata[10], Toshiki Terao[10], Masahiro Nakayama[11], Koichi Ohshima[5], Kensuke Usuki[12], Tatsuya Oda[6], Kosei Matsue[10], and Shigeru Chiba[2,3]*

*[1] Department of Hematology, Graduate School of Comprehensive Human Sciences, University of Tsukuba.*

*[2] Department of Hematology, Faculty of Medicine, University of Tsukuba.*

*[3] Department of Hematology, University of Tsukuba Hospital.*

*[4] Division of Advanced Hemato-Oncology, Transborder Medical Research Center, University of Tsukuba.*

*[5] Department of Pathology, School of Medicine, Kurume University.*

*[6] Department of Gastrointestinal and Hepato-Biliary-Pancreatic Surgery, Faculty of Medicine, University of Tsukuba.*

*[7] Department of Breast-Thyroid-Endocrine Surgery, University of Tsukuba Hospital.*

*[8] Department of Breast and Endocrine Surgery, Faculty of Medicine, University of Tsukuba.*

*[9] Department of Hematology, National Hospital Organization, Mito Medical Center.*

*[10] Division of Hematology/Oncology, Department of Internal Medicine, Kameda Medical Center.*

*[11] Department of Otolaryngology, Head and Neck Surgery, University of Tsukuba.*

*[12] Department of Hematology, NTT Medical Center Tokyo.*

** Correspondence*

**Related to "Human LN BECs: ten subclusters"**

The following is a comment regarding BEC subcluster-specific marker genes.

ABECs are characterized by selective expression of *GJA5* (Fig. 2h,i; Extended Data Fig. 2a), defined as a marker of larger arteries in murine tissues, including LNs[1,2]. By contrast, caBECs specifically express *SSUH2* (Fig. 2h,i; Extended Data Fig. 2a), a marker of arteries of varying size in different mouse tissues[1]. aBECs are characterized by expression of *INSR* (Fig. 2h; Extended Data Fig. 2a) and *EDNRB* (Fig. 2i), both capillary markers in mouse livers, lungs, and colon[1]. tBECs belong to a small but highly distinct subcluster that selectively expresses *PGF* and *LY6H* (Fig. 2h,i; Extended Data Fig. 2a). *PGF* is described as an endothelial tip cell marker in human lung cancer[3], whereas *LY6H* has not been shown to be a specific marker in mice or humans[1]. Other human tip cell markers, including *CXCL4*, *PXDN*, and *LXN*[3], were identified as DEGs in tBECs (Fig. 2h; Supplementary Table 5). cBECs are characterized by abundant *PLVAP* and *CA4* expression (Fig. 2h,i; Extended Data Fig. 2a), in agreement with mouse data describing them both as capillary markers in multiple tissues[1]. C-aHEVs express DEGs of cBECs (including *CA4*) and aHEVs (Fig. 2h,i; Supplementary Table 5), indicative of the transitioning nature of C-aHEVs. Also, *HES1*, a capillary marker in several mouse tissues, was highly expressed in C-aHEVs (Fig. 2h)[1]. C-aHEVs and aHEVs express genes encoding heat shock proteins (*HSPA1A*, *HSP90AA1*, and *HSPA1B*), as well as those involved in NFkB (*NFKBIA* and *NFKBIZ*) and JNK (*JUN*, *JUNB*, *JUND*, *FOS*, and

*FOSB*) activation. They also express genes associated with oscillatory shear stress (*EGR1*, *ICAM1*, *MT2A*, *MT2X*, and *ADM*) at higher levels than do cBECs and hHEVs (Extended Data Fig. 2b; Supplementary Tables 5,6)[4]. aHEVs, hHEVs, and CXCL10-HEVs all express genes specific to HEVs, including *ACKR1*, *C7*, and *CH25H* (Supplementary Table 5)[5]. CXCL10-HEVs are unique in their expression of the chemokines *CXCL9*, *CXCL10*, and *CXCL11* (Fig. 2h,i). Some suggest that a subset of HEVs express *CXCL10*, which facilitates trafficking of activated T cells and monocytes across HEVs[6,7], though others have not detected *CXCL10* mRNA in HEVs[8]. Therefore, the CXCL10 function in HEVs remains unclear[9]. Mouse LNs have a BEC subpopulation with highly similar phenotypes to CXCL10-HEVs that exhibit a prominent interferon signalling signature, although they were classified as capillary BECs[2]. VBECs are characterized by high expression of *CPE* and *VCAM1* (Fig. 2h; Extended Data Fig. 2a; Supplementary Table 5), which are markers of large veins[1,2].

We examined the expression of marker genes for mouse LN BEC subclusters[2] in our human BEC data and summarized an assumed correspondence relationship between mouse and human LN (Extended Data Fig. 2k,l).

**Related to "Human LN LECs: eight subclusters"**

The following discussion addresses results of unsupervised clustering in MFLN LECs, DEG

analysis across subclusters, trajectory analysis, and IF staining.

Putative cLECs (LEC I), particular SCS ceiling LECs that cover medullary regions (III), capillary LECs in surrounding tissues (IV), and valve LECs (V) were observed in our clustering, as suggested by Takeda et al and Xiang et al[10,11], though we annotated LECs III and IV as collectLECs and msLECs, respectively (Fig. 3a). We observed several slight differences between our analysis and previous analyses[10,11]. First, our analysis detected a subcluster in which gene expression patterns matched those seen in bLECs, a cell type proposed by Xiang et al (e.g. intermediate *BMP2* expression between cLECs and fLECs) (Fig. 3c; Extended Data Fig. 3a)[11], although bLECs were not detected by Takeda et al (Fig. 3e)[10]. Second, we initially could not identify fLECs and pfsLECs as distinct subclusters[10,11]. Lastly, two valve LEC subclusters (Up- and Down-valves) were identified as distinct, in contrast to a previous study that identified them after applying sub-clustering analysis to an initially-identified valve cluster (LEC V) (Fig. 3e)[10]. We speculate that these inconsistencies are due to the fact that previous studies collected LEC-enriched cells based on PDPN positivity in flow cytometric analysis, and thus LECs with relatively low PDPN expression may have been overlooked. Indeed, *PDPN* expression was particularly low in bLECs and down-valves (Extended Data Fig. 3a).

DEG analysis has revealed that the “fLEC and pfsLEC” subcluster is unique in harbouring more than twice as many DEGs as other LEC subclusters (Extended Data Fig. 3b). These DEGs

include chemokine genes such as *CXCL1*, *CXCL5*, and *CCL20*, and genes associated with immune cell adhesion, including *ICAM1*, *VCAM1*, and *SELE* (Extended Data Fig. 3a,c; Supplementary Table 8), supporting the immunological function of fLECs in cell trafficking and migration. Genes reportedly specific to pfsLECs (e.g. *MARCO* and *CLEC4G*[11]) were also detected as top DEGs for the "fLEC and pfsLEC" subcluster (Extended Data Fig. 3a,c; Supplementary Table 8)[10,11]. *ACKR4*, *MFAP4*, *CCL21*, *CLDN11*, and *GJA4* were also consistently identified as top DEGs for cLECs, collectLECs, msLEC, valve LECs (both Up- and Down-valves), and down-valves, respectively (Fig. 3c; Extended Data Fig. 3a,c)[10].

To verify that a single "fLEC and pfsLEC" subcluster consists of both fLECs and pfsLECs, we applied unbiased sub-clustering analysis to the "fLEC and pfsLEC" subcluster. As expected, it comprised two transcriptionally distinct populations (Extended Data Fig. 3d). In agreement with the findings of Takeda et al, the proportion of pfsLECs was smaller than that of fLECs (Extended Data Fig. 3e)[10]. Notably, in DEG analysis between fLECs and pfsLECs, genes described as specific to LEC II and VI[10] were separately detected as top DEGs for fLECs and pfsLECs, respectively (Extended Data Fig. 3f; Supplementary Table 9).

Next, we performed trajectory analysis on integrated LEC data. We identified all LEC subclusters in a *Monocle 3*-generated cell object (Extended Data Fig. 3g). As expected, bLECs and collectLECs were adjacent to cLECs (Extended Data Fig. 3h). Remarkably, a trajectory from fLEC

and pfsLEC subclusters connected with remaining LEC subclusters in the middle region of bLECs and msLECs, and a direct trajectory connection was observed between pfsLECs and msLECs (Extended Data Fig. 3h), supporting positional relationships inferred across these subclusters[11]. Both Up- and Down-valves were connected to collectLECs (Extended Data Fig. 3h), supporting the annotation of collectLECs (Fig. 3d).

IF staining showed that PAI1- (encoded by *SERPINE1*) positive bLECs were exclusively embedded in the intermediate area of the SCS (Extended Data Fig. 3i). PTX3-positive LECs (msLECs) were preferentially located at the medulla (Extended Data Fig. 3j), although a small proportion of cLECs also showed PTX3 positivity (Fig. 3c; Extended Data Fig. 3j). MARCO-positive LECs (pfsLECs) were preferentially situated at perifollicular sinuses (Extended Data Fig. 3k), as shown in mice[11]. MFAP4-positive LECs (collectLECs) were observed only in afferent and efferent collecting vessels (Extended Data Fig. 3l).

**Related to "Human LN NESCs: twelve subclusters"**

The following discussion addresses results regarding the annotation of TRCs, PCs, MRCs, and FDCs.

DEG analysis showed that TRCs were marked by high *CCL19* and *CCL21* expression (Fig. 4d,e; Supplementary Table 10)[12], and PCs by specific expression of *NDUFA4L2* and *HIGD1B* (Fig.

4d,e; Supplementary Table 10)[13]. Approximately half of MRC DEGs were seen in FDC DEGs (Supplementary Table 10), consistent with the idea that MRCs differentiate into FDCs[14]. FDC markers *FDCSP*, *CR2*, and *FCER2* were correctly detected as FDC-specific DEGs (Fig. 4d,e; Supplementary Table 10).

GO analysis revealed that TRCs were enriched with GOs related to leukocyte and dendritic cell migration (Fig. 4i; Supplementary Table 12), in accordance with their reported function in recruiting naive T cells and dendritic cells to the T-cell zone to facilitate interaction[12]. Genes associated with smooth muscle structure or contraction were enriched in PCs and in SMC subclusters, whereas PCs abundantly expressed genes associated with blood vessel development (Fig. 4i; Supplementary Table 12). MRCs and FDCs were highly enriched with GOs relevant to cytokine- and chemokine-mediated signalling and antigen processing and presentation (Fig. 4i; Supplementary Table 12), consistent with their role in positioning and presenting antigens to B cells[12]. Finally, FDCs expressed genes involved in immunoglobulin-mediated immune response and B-cell-mediated immunity (Fig. 4i; Supplementary Table 12)[12].

IF staining showed HIGD1B-positive cells (PCs) were observed around BECs, including arteries and HEVs (Extended Data Fig. 4n), and were more prevalent around HEVs in IFRs (aHEVs) than around hHEVs (Extended Data Fig. 4n), indicating that they may support lymphocyte recruitment and maintain endothelial cell integrity[12]. MRCs and FDCs were identified as BAFF- and

CR2 (also known as CD21)-positive, respectively (Extended Data Fig. 4o).

We examined the expression of key genes for mouse LN NESC subclusters[15] in our human NESC data and assumed a correspondence relationship between mouse and human LNs. Although some degree of correlation was detected between mouse and human LN NESCs, we observed more complicated heterogeneity in human LN NESCs than in mouse LNs and found that mouse NESC markers were not necessarily specific to human NESC counterparts (Extended Data Fig. 4q,r).

**Additional basic information of the single-cell LNNHC atlas**

All subclusters identified in the single-cell LNNHC atlas included cells from all MFLN samples with some variations in cell proportion (Extended Data Fig. 5a). Notably, NHCs from a patient with a benign tumour (MFLN 8) were distributed uniformly among NHCs from all MFLN samples (Extended Data Fig. 5b). Furthermore, in NHC subclusters harbouring at least 50 cells from the sample MFLN 8, 80–100% of the top DEGs for all MFLN sample subclusters matched MFLN 8 DEGs (Extended Data Fig. 5c).

We also profiled DEGs between mLN and pLN NHC subclusters using MFLN data (Supplementary Table 13). In this dataset, we observed markedly higher expression of *MADCAM1* in mLN HEV subclusters (aHEVs and hHEVs) compared with pLN counterparts (Extended Data Fig. 6a; Supplementary Table 13), in agreement with MADCAM1-dependent manners of

lymphocyte homing in mLN HEVs[7]. Consistently, GO analysis of DEGs between mLN and pLN subclusters showed that mLN HEV subclusters were enriched with *MADCAM1*-containing GO terms associated with cell adhesion and interactions with integrin (Extended Data Fig. 6b; Supplementary Table 14). We also found that multiple mLN NHC subclusters (particularly, NESC subclusters) highly expressed genes associated with inflammatory response including *PTX3*, *CCL2*, and *TNFAIP6* relative to pLN counterparts (Extended Data Fig. 6a,b; Supplementary Table 13). Consistent with the constant exposure of mLNs to enterobacteria-derived factors, GO terms associated with inflammatory response, TNF-signalling, and response to molecules of bacterial origin were enriched in mLN LEC and NESC subclusters (Extended Data Fig. 6b; Supplementary Table 14). Additionally, pLN LEC and NESC subclusters abundantly expressed genes associated with the maintenance of LN homeostasis and structures (Extended Data Fig. 6a,b; Supplementary Tables 13,14).

**Additional prognostic analyses**

We tested the prognostic value of the markers proposed in our analyses (*LY6H*, *LOX*, *TDO2*, and *REM1*) in the intermediate prognosis group of FL. Notably, we found that elevated *LOX* and *TDO2* expressions were prognostic even in the intermediate prognosis group, while the prognostic

efficiency of *LY6H* expression was slightly not significant and that of *REM1* expression was not prognostic (Extended Data Fig. 9f).

Since these prognostic markers were derived primarily from TRCs or tBECs, we developed a FL TRC signature (Supplementary Table 19) and evaluated its prognostic proficiency. However, we could not extract sufficient number of genes for developing a FL tBEC signature. As a result, we observed no significant difference in the overall survival of patients with high and low FL TRC signature (data not shown).

The upregulation of tip cell markers (*LY6H* and *LOX*) was prognostic in FL (Fig. 7a,b), suggesting that enhanced tumour angiogenesis might affect FL prognosis, hence we also examined the prognostic impact of conventional pan-BEC markers, *PECAM1* and *CDH5*[16]. However, we did not detect any adverse prognostic impact of these markers in the microarray dataset[17] (Extended Data Fig. 9g).

**Related to "Observation of NHC subclusters across lymphomas"**

The following addresses findings observed in scRNA-seq analysis of PTCL and tDLBCL stroma.

Unsupervised clustering analysis of integrated MFLN and lymphoma NHC data detected almost all NHC subclusters in both PTCL and tDLBCL (Extended Data Fig. 10a–d). Furthermore, we found that BECs and LECs in these lymphomas consistently increased and decreased,

respectively, as observed in FL (Extended Data Fig. 10b,d). tDLBCL BECs contained abundant arterial subclusters and CXCL10-HEVs (Extended Data Fig. 10d), suggesting accelerated angiogenesis and elevated HEV activities. Furthermore, we observed considerable alterations in the tDLBCL NESC proportions compared with the MFLN counterparts: the proportion of advSCs, medullary SCs, TNF-SCs, C7-SCs, AGT-SCs, and TRCs decreased considerably, whereas that of FSCs (MRCs and FDCs) increased drastically in tDLBCL, and the perivascular cell subcluster compositions remained relatively comparable (Extended Data Fig. 10d). Representative DEGs upregulated in FL subclusters, including *LY6H* and *LOX* at tBECs, *SELE* at HEV subclusters, and *VCAM1* at FSCs, were also observed in tDLBCL stroma, although some key DEGs were not validated, probably because of the small sample size of tDLBCL cohort (Extended Data Fig. 10e). In line with the findings of analyses on MFLN and FL NESCs (Fig. 4g,h, 5a), trajectories in tDLBCL NESCs indicated that non-FSC subclusters had differentiated into FSCs (Extended Data Fig. 10f–h), likely representing a terminal form of stromal remodelling in FL.

**Supplementary references**


1. Kalucka, J., *et al.* Single-Cell Transcriptome Atlas of Murine Endothelial Cells. *Cell* **180**, 764-779.e720 (2020).
2. Brulois, K., *et al.* A molecular map of murine lymph node blood vascular endothelium at single cell resolution. *Nature communications* **11**, 3798 (2020).
3. Goveia, J., *et al.* An Integrated Gene Expression Landscape Profiling Approach to Identify Lung Tumor Endothelial Cell Heterogeneity and Angiogenic Candidates. *Cancer cell* **37**, 21-36.e13 (2020).
4. Bondareva, O., *et al.* Identification of atheroprone shear stress responsive regulatory elements in endothelial cells. *Cardiovascular research* **115**, 1487-1499 (2019).
5. Lee, M., *et al.* Transcriptional programs of lymphoid tissue capillary and high endothelium reveal control mechanisms for lymphocyte homing. *Nature immunology* **15**, 982-995 (2014).
6. Nagakubo, D., *et al.* A high endothelial venule secretory protein, mac25/angiomodulin, interacts with multiple high endothelial venule-associated molecules including chemokines. *Journal of immunology (Baltimore, Md. : 1950)* **171**, 553-561 (2003).
7. von Andrian, U.H. & Mempel, T.R. Homing and cellular traffic in lymph nodes. *Nature reviews. Immunology* **3**, 867-878 (2003).
8. Janatpour, M.J., Hudak, S., Sathe, M., Sedgwick, J.D. & McEvoy, L.M. Tumor necrosis

factor-dependent segmental control of MIG expression by high endothelial venules in inflamed lymph nodes regulates monocyte recruitment. *The Journal of experimental medicine* **194**, 1375-1384 (2001).

9. Miyasaka, M. & Tanaka, T. Lymphocyte trafficking across high endothelial venules: dogmas and enigmas. *Nature reviews. Immunology* **4**, 360-370 (2004).

10. Takeda, A.*, et al.* Single-Cell Survey of Human Lymphatics Unveils Marked Endothelial Cell Heterogeneity and Mechanisms of Homing for Neutrophils. *Immunity* **51**, 561-572.e565 (2019).

11. Xiang, M.*, et al.* A Single-Cell Transcriptional Roadmap of the Mouse and Human Lymph Node Lymphatic Vasculature. *Frontiers in cardiovascular medicine* **7**, 52 (2020).

12. Krishnamurty, A.T. & Turley, S.J. Lymph node stromal cells: cartographers of the immune system. *Nature immunology* **21**, 369-380 (2020).

13. Vanlandewijck, M.*, et al.* A molecular atlas of cell types and zonation in the brain vasculature. *Nature* **554**, 475-480 (2018).

14. Fletcher, A.L., Acton, S.E. & Knoblich, K. Lymph node fibroblastic reticular cells in health and disease. *Nature reviews. Immunology* **15**, 350-361 (2015).

15. Rodda, L.B.*, et al.* Single-Cell RNA Sequencing of Lymph Node Stromal Cells Reveals Niche-Associated Heterogeneity. *Immunity* **48**, 1014-1028.e1016 (2018).

16. Taskinen, M., *et al.* Prognostic impact of CD31-positive microvessel density in follicular lymphoma patients treated with immunochemotherapy. *European journal of cancer (Oxford, England : 1990)* **46**, 2506-2512 (2010).

17. Leich, E., *et al.* Follicular lymphomas with and without translocation t(14;18) differ in gene expression profiles and genetic alterations. *Blood* **114**, 826-834 (2009).
