# Supplementary material for: A single-cell atlas of non-haematopoietic cells in human lymph nodes and lymphoma reveals a landscape of stromal remodelling
Source: Nat Cell Biol. 2022 Mar 24;24(4):565–78. doi: 10.1038/s41556-022-00866-3 (PMC9033586; doi:10.1038/s41556-022-00866-3)
Supplement: Supplementary file 2 — Reporting Summary [file 41556_2022_866_MOESM2_ESM.pdf]

Corresponding author(s): Mamiko Sakata-Yanagimoto

Last updated by author(s): Jan 20, 2022

Reporting Summary

Nature Research wishes to improve the reproducibility of the work that we publish. This form provides structure for consistency and transparency in reporting. For further information on Nature Research policies, see our Editorial Policies and the Editorial Policy Checklist.

Statistics

For all statistical analyses, confirm that the following items are present in the figure legend, table legend, main text, or Methods section.

n/a	Confirmed	
☐	☒	The exact sample size (*n*) for each experimental group/condition, given as a discrete number and unit of measurement
☐	☒	A statement on whether measurements were taken from distinct samples or whether the same sample was measured repeatedly
☐	☒	The statistical test(s) used AND whether they are one- or two-sided<br>*Only common tests should be described solely by name; describe more complex techniques in the Methods section.*
☐	☒	A description of all covariates tested
☐	☒	A description of any assumptions or corrections, such as tests of normality and adjustment for multiple comparisons
☐	☒	A full description of the statistical parameters including central tendency (e.g. means) or other basic estimates (e.g. regression coefficient) AND variation (e.g. standard deviation) or associated estimates of uncertainty (e.g. confidence intervals)
☐	☒	For null hypothesis testing, the test statistic (e.g. *F*, *t*, *r*) with confidence intervals, effect sizes, degrees of freedom and *P* value noted<br>*Give P values as exact values whenever suitable.*
☒	☐	For Bayesian analysis, information on the choice of priors and Markov chain Monte Carlo settings
☐	☒	For hierarchical and complex designs, identification of the appropriate level for tests and full reporting of outcomes
☐	☒	Estimates of effect sizes (e.g. Cohen's *d*, Pearson's *r*), indicating how they were calculated

Our web collection on statistics for biologists contains articles on many of the points above.

Software and code

Policy information about availability of computer code

Data collection	FACSAria II and III (BD Biosciences) were used for acquisition of flow cytometry data.<br>Chromium Single Cell 3’ Reagent kits (V3) (10X Genomics) were used for single-cell RNA library preparation.<br>Libraries were sequenced on an Illumina HiSeq X Ten system, mapped to the human genome (build GRCh38), and demultiplexed using CellRanger pipelines (10x Genomics, version 3.1.0).
Data analysis	Flowcytometric data were analyzed using FlowJo software (Tree Star Inc. v10.7.1).<br>Single-cell data was analyzed using R package Seurat (R. Satija Lab. v3.2.2) on RStudio (v3.5.0 or v4.0.2).<br>Monocle 3 package (Trapnell Lab. v0.2.3) was used for trajectory analysis on single-cell data.<br>CellPhoneDB package (Teichlab. v2.1.1) was used on Python (v3.6) for intercellular ligand–receptor interaction analysis.<br>GO enrichment analysis was performed using metascape (http://metascape.org).<br>GSVA package (https://github.com/rcastelo/GSVA. v1.38.2) was used for gene signature analyses.<br>DecontX (in celda package. Campbell Lab. v1.6.1) and SoupX (v1.5.2) were used for the detection of ambient RNA contamination.<br>For analysis of whole-exome sequencing, the Genomon2 pipeline (v2.6.2) was used for sequence alignment and mutation calling.<br>Statistical analyses were performed using R on Rstudio or GraphPad Prism 9 (GrphPad, v9.2.0)<br>Please find detailed descriptions for each analysis in the Methods section.<br><br>The codes for key computational analyses are available on GitHub at http://github.com/yoshiakiabe1018/Stroma01.

For manuscripts utilizing custom algorithms or software that are central to the research but not yet described in published literature, software must be made available to editors and reviewers. We strongly encourage code deposition in a community repository (e.g. GitHub). See the Nature Research guidelines for submitting code & software for further information.

Data

Policy information about availability of data

All manuscripts must include a data availability statement. This statement should provide the following information, where applicable:

- Accession codes, unique identifiers, or web links for publicly available datasets
- A list of figures that have associated raw data
- A description of any restrictions on data availability

scRNA-seq data that support the findings of this study have been deposited at the European Genome-Phenome Archive (https://ega-archive.org) database and can be retrieved using the accession number EGAD00001008311. For survival analysis, a DNA microarray dataset from Leich et al66 was downloaded from the Gene Expression Omnibus (GEO) (accession number: GSE16131). For mapping of scRNA-seq data, GRCh38 (https://www.ncbi.nlm.nih.gov/assembly/GCF_000001405.39) was used. All other data are available from the corresponding authors on reasonable request. Source data are provided with this paper.

Field-specific reporting

Please select the one below that is the best fit for your research. If you are not sure, read the appropriate sections before making your selection.

☒ Life sciences ☐ Behavioural & social sciences ☐ Ecological, evolutionary & environmental sciences

For a reference copy of the document with all sections, see nature.com/documents/nr-reporting-summary-flat.pdf

Life sciences study design

All studies must disclose on these points even when the disclosure is negative.

Sample size	No statistical method was used to determine sample size a priori. The number of human lymph node and lymphoma samples was highly restricted due to the limited availability of these samples in clinical settings.
Data exclusions	Pre-processed single-cell data from each sample were further processed and analysed individually using R package Seurat on RStudio. After removing ribosomal genes, genes expressed in fewer than 3 cells, and cells expressing fewer than 200 genes, we filtered out cells with less than 200 unique feature counts (low quality cells). Cells with unique feature counts greater than three times the median value (possible doublets) and/or cells with more than twice the median number of mitochondrial genes (possible apoptotic or lysed cells) were also removed. After the data integration and clustering analysis, we removed data of NHC subclusters considered possible doublets as characterized by high expressions of marker genes for different NHC components and incongruously high unique feature counts.
Replication	All experiments were independently replicated at least once to verify reproducibility.
Randomization	Not relevant - no treatment group.
Blinding	Not relevant - no treatment group.

Reporting for specific materials, systems and methods

We require information from authors about some types of materials, experimental systems and methods used in many studies. Here, indicate whether each material, system or method listed is relevant to your study. If you are not sure if a list item applies to your research, read the appropriate section before selecting a response.

Materials & experimental systems

n/a	Involved in the study
☐	☒ Antibodies
☒	☐ Eukaryotic cell lines
☒	☐ Palaeontology and archaeology
☒	☐ Animals and other organisms
☐	☒ Human research participants
☒	☐ Clinical data
☒	☐ Dual use research of concern

Methods

n/a	Involved in the study
☒	☐ ChIP-seq
☐	☒ Flow cytometry
☒	☐ MRI-based neuroimaging

Antibodies

Antibodies used	Flow cytometric analysis: PE-anti-CD45 (BioLegend. Cat# 304058), AF488-anti-pan-cytokeratin (ThermoFisher Scientific. Cat# 53-9003-82), FITC-anti-CD31 (Biolegend, 303103), APC-anti-podoplanin (Biolegend. Cat# 337021), PE-Cy7-anti-CD31 (Biolegend. Cat# 303117), PE-Cy7-anti-CD34 (Biolegend, 343515). PE-anti-CD27 (Biolegend, 302842), FITC-anti-CD3 (Biolegend, 300406), APC-anti-CD19 (Miltenyi Biotec, 130-113-165), PE-Cy7-anti-CD10 (Biolegend. Cat# 312214). The dilution for each antibody is described in the Methods section of the manuscript.

Recombinant protein binding assay: PE-anti-human IgG Fc (R&D systems, FAB110P). The dilution is described in the Methods section of the manuscript.

Functional blocking: anti-CD27 blocking antibody (R&D systems, MAB382), isotype mouse IgG1 (R&D systems, MAB002).

Antibodies used for immunofluorescence staining were listed in Supplementary Table 9.

Validation

All the antibodies used in this study have been tested by the manufacturer and have been cited by other authors and references are available on the manufacturer's websites. We provide catalog numbers for all the antibodies in the Methods section of the manuscript or in Supplementary Table 9 as readers can retrieve the information of the antibodies. We have further evaluated the specificity of the antibodies in our samples by analyzing the distribution of the antibody signals and the absence of the antibody signals in the regions where the target protein was not supposed to be expressed.

Human research participants

Policy information about studies involving human research participants

Population characteristics

Supplementary Table 1 summarises the characteristics of patients in metastasis-free lymph node and follicular lymphoma cohorts. Metastasis-free lymph node cohort consists of neoplasm-bearing patients (n = 9) who had undergone surgical LN dissection. The median age of the patients in this cohort is 66 years old. Follicular lymphoma cohort consists of 10 patients with the median age of 59. Among the follicular lymphoma patients, six patients were newly diagnosed cases. Most of the follicular lymphoma patients (n = 9) were with pathological grade of 1-2.
Supplementary Table 7 summarises the characteristics of patients in peripheral T-cell lymphoma cohort and diffuse large B-cell lymphoma transformed from follicular lymphoma cohort. Peripheral T-cell lymphoma cohort consists of five newly diagnosed patients with various subtypes of lymphoma with the median age of 78. Diffuse large B-cell lymphoma transformed from follicular lymphoma cohort consists of three patients.
Additional follicular lymphoma samples for functional experiments were collected from eight patients. Characteristics of follicular lymphoma patients in the additional cohort is summarised in Supplementary Table 10.

Recruitment

Samples were prospectively collected from patients who agreed to participate in the study. There were no other criteria for patient selection. There is no self-selection bias or other biases in recruitment.

Ethics oversight

This study was approved by the Ethics Committee of the University of Tsukuba Hospital and the review boards of associated institutions that provided human samples (Kameda Medical Center, NTT Medical Center Tokyo, and Mito Medical Center) and conducted according to all relevant ethical regulations regarding human patients. Written informed consent was obtained from all participating patients. The participants were not compensated for their participation.

Note that full information on the approval of the study protocol must also be provided in the manuscript.

Flow Cytometry

Plots

Confirm that:

☒ The axis labels state the marker and fluorochrome used (e.g. CD4-FITC).

☒ The axis scales are clearly visible. Include numbers along axes only for bottom left plot of group (a 'group' is an analysis of identical markers).

☒ All plots are contour plots with outliers or pseudocolor plots.

☒ A numerical value for number of cells or percentage (with statistics) is provided.

Methodology

Sample preparation

Single-cell isolation of LN non-haematopoietic cells: After collection, LN or lymphoma samples were immediately minced and digested for 1 h with RPMI 1640 medium (Sigma-Aldrich, R8758) with 5% fetal bovine serum containing 0.2 mg/ml collagenase P (Sigma-Aldrich, 11213857001), 0.8 mg/ml dispase (Gibco, 17105041), and 0.1 mg/ml DNase 1 (Worthington, LS002139), with continuous agitation. Cells were then filtered through a 70 µm mesh and red blood cells were lysed in 1% ammonium-chloride-potassium buffer. Thereafter, haematopoietic cells and contaminated red blood cells were depleted using human CD45 (130-045-801) and CD235a (130-050-501) microbeads according to the manufacturer's instructions (Miltenyi Biotec). For MFLN samples, the remaining single-cell suspension was incubated with phycoerythrin (PE)-anti-CD45 (Biolegend) in combination with Alexa Fluor 488-pan-cytokeratin (ThermoFisher Scientific), allophycocyanin (APC)-anti-podoplanin (Biolegend), and PE-cyanin 7 (PE-Cy7)-anti-CD31 (Biolegend). For lymphoma samples, PE-anti-CD45 was mixed with fluorescein isothiocyanate-anti-CD31 (Biolegend), APC-anti-podoplanin, and PE-Cy7-anti-CD34 (Biolegend). The samples were incubated for 20 min, then 7-AAD Viability Staining Solution (ThermoFisher Scientific, 00-6993-50) was added and incubated for 10 min in the dark on ice. CD45-negative live cells were sorted using FACSAria II or III (BD Bioscience) after removing doublets by gating with a FSC-H versus FCS-W plot and a SSC-H versus SSC-W plot.

Single-cell isolation of FL haematopoietic cells: After thawing, cell suspensions were filtered through a 70 µm mesh and incubated with 7-AAD Viability Staining Solution for 10 min in the dark.

Flow cytometric analysis of FL haematopoietic cells: After thawing, cells were filtered through a 70 µm mesh, and incubated with PE-anti-CD27 (Biolegend), FITC-anti-CD3 (Biolegend), APC-anti-CD19 (Miltenyi Biotec), and PE-Cy7-anti-CD10 (Biolegend)

antibodies for 20 min on ice. Cells were then incubated with 7-AAD Viability Staining Solution for 10 min in the dark and analysed using FACSAria II or III.

Recombinant protein binding assay: Recombinant Fc chimera CD70 (SinoBiological, 10780-H01H) or human IgG (R&D systems, 1-001-A) was incubated with a single-cell suspension of FL haematopoietic cells for 10 min at 4 ºC in RPMI with 10% FCS. To block CD70–CD27 binding, malignant B cells were incubated in the presence of anti-CD27 blocking antibody (R&D systems, MAB382) or isotype mouse IgG1 (R&D systems, MAB002) for 30 min at 4 ºC before binding. After binding, the cells were washed, fixed by 4% PFA for 10 min at 20 ºC and incubated with PE-anti-human IgG Fc (R&D systems), FITC-anti-CD3, APC-anti-CD19, and PE-Cy7-anti-CD10 for 20 min at 4 ºC.

Instrument

FACSAria II and III (BD Biosciences)

Software

Data collection: FACSAria II and III (BD Biosciences)
Data analysis: FlowJo (Tree Star Inc. v10.7.1)

Cell population abundance

The purity of sorted fractions was not determined for each sorting because FACS was used justed to enrich cell fractions of interest, followed by scRNA-seq which finally identifies the cell-type of sorted cells.

Gating strategy

All captured cells were first gated for singlet cells with a FSC-H versus FCS-W plot and a SSC-H versus SSC-W plot. Thereafter, dead cells were removed as 7-AAD-positive cells.
Non-haematopoietic cells: From singlets, CD45-negative (SSC-H vs PE-CD45).
Blood endothelial cells: From non-haematopoietic cells, CD31-positive and PDPN-negative (PE-Cy7-CD31 vs APC-PDPN).
Lymphatic endothelial cells: From non-haematopoietic cells, CD31-positive and PDPN-positive (PE-Cy7-CD31 vs APC-PDPN).
Non-endothelial stromal cells: From non-haematopoietic cells, CD31-negative (PE-Cy7-CD31 vs APC-PDPN).
FL malignant B cells: From singlets, CD19-positive (SSC-H vs APC-CD19), followed by CD10-positive (SSC-H vs PE-Cy7-CD10).
FL non-malignant B cells: From singlets, CD19-positive (SSC-H vs APC-CD19), followed by CD10-negative (SSC-H vs PE-Cy7-CD10).
CD70-Fc-bound FL malignant B cells: From FL malignant B cells, human IgG Fc-positive (SSC-H vs PE-anti-human IgG Fc).

☒ Tick this box to confirm that a figure exemplifying the gating strategy is provided in the Supplementary Information.
