# Supplementary material for: A single-cell atlas of non-haematopoietic cells in human lymph nodes and lymphoma reveals a landscape of stromal remodelling
Source: Nat Cell Biol. 2022 Mar 24;24(4):565–78. doi: 10.1038/s41556-022-00866-3 (PMC9033586; doi:10.1038/s41556-022-00866-3)
Supplement: Supplementary file 3 — Peer Review Information [file 41556_2022_866_MOESM3_ESM.pdf]

natureresearch

Peer Review Information

Journal: Nature Cell Biology
Manuscript Title: A single-cell atlas of non-haematopoietic cells in human lymph nodes and lymphoma reveals a landscape of stromal remodelling
Corresponding author name(s): Mamiko Sakata-Yanagimoto, Shigeru Chiba

Reviewer Comments & Decisions:

Decision Letter, initial version:

Subject: Decision on Nature Cell Biology submission NCB-A45004
Message:

*Please delete the link to your author homepage if you wish to forward this email to co-authors.

Dear Dr Sakata-Yanagimoto,

Please first accept our apology for the delay getting back to you with a decision due to difficulties in retrieving reviewer comments.

Your manuscript, "A comprehensive single-cell atlas of nonhematopoietic cells in human lymph node and lymphoma reveals landscape of stromal remodeling", has now been seen by 3 referees, who are experts in stroma and LNs (referee 1); lymphatic vasculature and LNs (referee 2); and single cell analysis in immunology (referee 3). As you will see from their comments (attached below) they find this work of potential interest, but have raised substantial concerns, which in our view would need to be addressed with considerable revisions before we can consider publication in Nature Cell Biology.

Nature Cell Biology editors discuss the referee reports in detail within the editorial team, including the chief editor, to identify key referee points that should be addressed with priority, and requests that are overruled as being beyond the scope of the current study. To guide the scope of the revisions, I have listed these points below. We are committed to providing a fair and constructive peer-review process, so please feel free to contact me if you would like to discuss any of the referee comments further.

In particular, it would be essential to:

A) Address the concern related to 'homeostatic' LNs as questioned by reviewers:

Reviewer 1
"A major issue for this paper is the framing of the comparator lymph nodes as ‘healthy’. The lymph nodes referred to throughout the paper as ‘homeostatic lymph nodes’ or HLN are in fact not from healthy subjects. On the contrary, they are lymph nodes from patients with a variety of solid cancers and rather disconcertingly this was not presented upfront. Thus, these comparator lymph nodes should not be considered ‘homeostatic’ unless the authors can provide evidence that the LNs from solid-tumor bearing patients are in fact equivalent to LNs from healthy subjects or non-reactive. The paper would benefit immensely from having comparison data derived from truly ‘homeostatic’ LNs. Without these data the manuscript title, text and conclusions will need to be modified to reflect the nature of the LNs being used as comparators with the lymphoma samples."

Reviewer 2
"...The authors have tried to exclude the anatomic variation by additional comparisons of lymphoma LN to only those 3 “homeostatic” LN which were of non-mesenteric origins. These data seem to be largely hidden in Suppl. Table 3, and reveal a high proportion of non-validated hits. This issue should be elaborated more. The authors largely miss here a unique cell biological opportunity to unravel the differences in stromal cell compartments of peripheral and mesenteric LN in humans under “homeostatic” conditions (only DEGs listed in Suppl. Table 2). For instance, the stromal cells of these two LN systems are known to represent different leukocyte trafficking routes, they are differentially exposed to intestinal lipids and microbial products etc."

B) Provide further validation data as requested by all reviewers:

Reviewer 1
"On page 5 and in Figure 1f and Suppl. Figure 1f the authors reference flow cytometry analysis to validate the proportions of LECs and BECs. Can the authors comment on the flow cytometry data for NESC (PDPN+ and PDPN-)? Are the proportions of NESC concordant with the scRNAseq data?"

Reviewer 2
"The proposed widespread lymphatic EC damage in follicular lymphoma (p. 13, l10; p15, l6) is interesting. Very minimally, it should be verified by immunostainings. In addition, Fig. 5d indicates induction of molecules involved in antigen presentation (HLA, CD74) in these lymphatic EC, which could be functionally validated (see 3.)"

"To validate the robustness of the in silico interactome analyses (Fig. 6), at least a representative novel interaction should be verified experimentally. For instance, ex vivo Stamper-Woodruff-type binding

assays using frozen sections and lymphoma cells in conjunction with blocking antibodies could give support to relevance of the predicted interactions."

Reviewer 3
"The reading is somewhat dry and the paper appears to be a laundry list of putative cell types- as the authors themselves point out, they have not done any work to functionally validate their claims. This seems to be an especially strong defect in the cell-communication analysis using cellphoneDB, which is entirely informatic and speculative."

C) Improved characterization of gene expression as noted by all reviewers:

Reviewer 1
"On line 12 the authors refer to BAFF, IL-15 and HGF as FDC-derived molecules. Can the authors analyze the expression of these genes across the NHC subtypes in their scRNAseq data and comment on the pattern? This will help the readers understand which single NHC type in human LN and FL express the genes encoding these molecules."

"Can the authors explain why they chose the genes shown in Figure 5d? Figure 5d would be strengthened and more informative by showing the expression of all genes across each of the cell subtypes. All key genes referenced on page 14 should be shown in a main figure (Cxcl13, Postn, Fap, Egfl6 across cell subtypes."

" On page 14, the authors discuss gene expression related to lymphocyte migration and draw conclusions about the cell types providing the relevant cues. The data in Suppl. Figure 5 is potentially interesting. What are the gene differences between the GO categories: 'positive regulation of leukocyte chemotaxis', 'mononuclear cell migration', and 'regulation of lymphocyte migration' to help explain the differences between TRC and MRC?"

"On page 17 and in other places in the manuscript the authors refer to FDCs as key sources of Cxcl13 and yet the data in this manuscript do not support these conclusions. In fact, their data showed that Cxcl13 expression is extremely low in FDCs whereas MRCs express relatively high levels."

Reviewer 2
"The identified specific markers should be exploited for prospective purification (FACS sort) of a given LN cell population for cell biological experiments. E.g. the transdifferentiating cells, tip-like (and Ly6H+) blood EC, ATF3 hi and low smooth muscle cells could be purified to validate their unique characteristics (now only speculated in the text) using selected proof-of-concept wet-lab experiments."

"The prognostic implications of the identified stromal markers should be analyzed further. The selected markers should also be used as continuous variables and their value in stratifying the clinically challenging intermediate prognosis group should be tested. In addition, it has already been reported that angiogenesis is a poor prognostic marker in follicular lymphoma (e.g. PMID: 20713461, PMID: 20630741). Do the proposed novel markers bring any additional value in comparison to pan-endothelial markers (CD31, VE-cadherin, Plvap etc) in the current data set? Fig. 7c should include more patients and quantification."

Reviewer 3
"The analysis of the pathological samples seems incomplete. What are the gene expression signatures that are clearly neoplastic, especially in the B cells? Can the authors identify genomic alterations related to cancer in their transcriptome data from those samples?"

D) All other referee concerns pertaining to strengthening existing data, providing controls, methodological details, clarifications and textual changes, as applicable should also be addressed.

E) Finally please pay close attention to our guidelines on statistical and methodological reporting (listed below) as failure to do so may delay the reconsideration of the revised manuscript. In particular please provide:

- a Supplementary Figure including unprocessed images of all gels/blots in the form of a multi-page pdf file. Please ensure that blots/gels are labeled and the sections presented in the figures are clearly indicated.

- a Supplementary Table including all numerical source data in Excel format, with data for different figures provided as different sheets within a single Excel file. The file should include source data giving rise to graphical representations and statistical descriptions in the paper and for all instances where the figures present representative experiments of multiple independent repeats, the source data of all repeats should be provided.

We would be happy to consider a revised manuscript that would satisfactorily address these points, unless a similar paper is published elsewhere, or is accepted for publication in Nature Cell Biology in the meantime.

When revising the manuscript please:

- ensure that it conforms to our format instructions and publication policies (see below and www.nature.com/nature/authors/).

- provide a point-by-point rebuttal to the full referee reports verbatim, as provided at the end of this letter.

- provide the completed Editorial Policy Checklist (found here https://www.nature.com/authors/policies/Policy.pdf), and Reporting Summary (found here https://www.nature.com/authors/policies/ReportingSummary.pdf). This is essential for reconsideration of the manuscript and these documents will be available to editors and referees in the event of peer review. For more information see http://www.nature.com/authors/policies/availability.html or contact me.

Nature Cell Biology is committed to improving transparency in authorship. As part of our efforts in this direction, we are now requesting that all authors identified as 'corresponding author' on published papers create and link their Open Researcher and Contributor Identifier (ORCID) with their account on the Manuscript Tracking System (MTS), prior to acceptance. ORCID helps the scientific community achieve unambiguous attribution of all scholarly contributions. You can create and link your ORCID from the home page of the MTS by clicking on 'Modify my Springer Nature account'. For more information please visit please visit www.springernature.com/orcid.

Please submit the revised manuscript files and the point-by-point rebuttal to the referee comments using this link:

[REDACTED]

*This url links to your confidential home page and associated information about manuscripts you may have submitted or be reviewing for us. If you wish to forward this email to co-authors, please delete the link to your homepage.

We would like to receive a revised submission within six months. We would be happy to consider a revision even after this timeframe, however if the resubmission deadline is missed and the paper is eventually published, the submission date will be the date when the revised manuscript was received.

We hope that you will find our referees' comments, and editorial guidance helpful. Please do not hesitate to contact me if there is anything you would like to discuss.

Best wishes,

Zhe Wang

Zhe Wang, PhD

Senior Editor
Nature Cell Biology

Tel: +44 (0) 207 843 4924
email: zhe.wang@nature.com

Reviewers' Comments:

Reviewer #1:
Remarks to the Author:
The manuscript titled " A comprehensive single-cell atlas of nonhematopoietic cells in human lymph node and lymphoma reveals landscape of stromal remodeling", provides a deep comparative examination of non-hematopoietic cells in lymph nodes from 10 FL patients and 9 non-FL patients using single cell RNA sequencing, flow cytometry and immunohistochemistry. While the scRNAseq data generated and presented in this manuscript are of high quality and will be a useful resource for the research community several major concerns will need to be adequately addressed.

Points to address:

1) A major issue for this paper is the framing of the comparator lymph nodes as ‘healthy’. The lymph nodes referred to throughout the paper as ‘homeostatic lymph nodes’ or HLN are in fact not from healthy subjects. On the contrary, they are lymph nodes from patients with a variety of solid cancers and rather disconcertingly this was not presented upfront. Thus, these comparator lymph nodes should not be considered ‘homeostatic’ unless the authors can provide evidence that the LNs from solid-tumor bearing patients are in fact equivalent to LNs from healthy subjects or non-reactive. The paper would benefit immensely from having comparison data derived from truly ‘homeostatic’ LNs. Without these data the manuscript title, text and conclusions will need to be modified to reflect the nature of the LNs being used as comparators with the lymphoma samples.
2) In the abstract the authors refer to 27 human samples however only 19 samples (NHL and FL) were deeply analyzed. The DLBCL and PTCL samples may have been analyzed but were not used as comparators with the FL or characterized as deeply.
3) On line 12 the authors refer to BAFF, IL-15 and HGF as FDC-derived molecules. Can the authors analyze the expression of these genes across the NHC subtypes in their scRNAseq data and comment on the pattern? This will help the readers understand which single NHC type in human LN and FL express the genes encoding these molecules.

4) On page 5 and in Figure 1f and Suppl. Figure 1f the authors reference flow cytometry analysis to validate the proportions of LECs and BECs. Can the authors comment on the flow cytometry data for NESC (PDPN+ and PDPN-)? Are the proportions of NESC concordant with the scRNAseq data?
5) The data in Figure 5 (and in many figures) lack statistical testing to clarify whether differences in proportions are significant. Where proportions increase or decrease the authors should also comment on the concomitant changes.
6) The font in many figures is too small. For example, the data and labels in Figure 5d are much too small. One should not need to zoom in to see every subfigure. This figure could be improved by generating heatmaps that show each patient sample.
7) Can the authors explain why they chose the genes shown in Figure 5d? Figure 5d would be strengthened and more informative by showing the expression of all genes across each of the cell subtypes. All key genes referenced on page 14 should be shown in a main figure (Cxcl13, Postn, Fap, Egfl6 across cell subtypes.
8) Can the authors provide data on the total cellularity, mass and size or volume of the lymph nodes used in the study and provide information on the numbers of each NHC subtype?
9) On page 14, the authors discuss gene expression related to lymphocyte migration and draw conclusions about the cell types providing the relevant cues. The data in Suppl. Figure 5 is potentially interesting. What are the gene differences between the GO categories: 'positive regulation of leukocyte chemotaxis', 'mononuclear cell migration', and 'regulation of lymphocyte migration' to help explain the differences between TRC and MRC?
10) On lines 6-7, page 15 the authors refer to widespread lymphatic damage to account for the significant decrease in LECs in FL. This statement needs to be substantiated by imaging data.
11) The authors may want to further clarify the significance of the analysis in Supplementary Figure 8.
12) On page 17 and in other places in the manuscript the authors refer to FDCs as key sources of Cxcl13 and yet the data in this manuscript do not support these conclusions. In fact, their data showed that Cxcl13 expression is extremely low in FDCs whereas MRCs express relatively high levels.
13) The data in Figure 7c is potentially interesting. The authors need to provide quantitation for each sample and across patients including statistical analysis. LOX and LY6H expression overlaps with PLVAP and MECA79 in some cells but numerous single positive (red) cells are evident in the FL samples but not the NHL. What are these cells?
14) In Figure 7c, the TDO2 and REM1 staining is clearly not overlapping with CD31 but no marker for TRC was used to confirm the identity of these cells. This should be addressed with staining for a TRC marker.
15) The survival analysis could be strengthened by examining gene signatures for each cell subset. For example, would a signature for tip cells be associated with reduced survival similar to LY6H and LOX? Likewise for TRC?
16) Are LY6H, LOX, REM1 and TDO2 poor prognostic factors in DLBCL and PTCL?
17) Statistical tests and appropriate values should be included in all relevant figures and clarified in the text or legends.

18) The number of single cells analyzed in each UMAP should be provided in the figures or in the figure legends.

Reviewer #2:
Remarks to the Author:
Manuscript NCB-A45004 provides a detailed single-cell RNAseq based atlas of non-leukocytic cell types in human lymph nodes (LN). The samples include metastasis-free LN (called homeostatic LN) from different cancer patients (mainly colorectal cancer) and affected LN from patients with follicular and other lymphomas. In “homeostatic” LN the authors identified 10 blood endothelial cell (EC), 8 lymphatic EC and 12 non-EC stromal cell clusters. They report the frequencies of different cell types, the differentially expressed genes, gene ontology analyses and potential developmental associations using standard bioinformatic pipelines. They also analyze selected DEGs using immunohistochemistry. Finally, the authors compare the stromal cell types of “homeostatic” LN to those seen in follicular lymphoma (10 patients; and superficially to cells isolated from 5 T-cell lymphoma and 3 diffuse large B cell lymphoma patients) to find lymphoma-selective alterations, potential receptor-ligand interactions between stromal cells and malignant cells and potential new prognostic factors.

The analyses of blood EC and non-EC compartments in “homeostatic” human LN as well as analyses of all stromal cell types in lymphoma patients are novel. Similar analyses of all stromal cell types in homeostatic and inflamed LN have been previously performed in mice, and for lymphatic EC also in humans. Potentially the current resource atlas will be significant and useful for lymph node and lymphoma researchers.

Quality and presentation of the data, including statistical analyses are, in general, solid. Conclusions are mainly supported by the data. The discussion partly recapitulates the result section rather than expands it. The potential functional relevance of the observed hits/interactions is solely based on literature-based speculations (with appropriate wording). The manuscript is well-written, and the majority of relevant previous literature is appropriately cited.

My main concerns relate to the limitations in the study set-up, validation and mechanistic insight.

1. The homeostatic LNs were mainly from mesenteric LN draining the gut, while the lymphoma samples were from peripheral (mainly cervical) LN draining the oral cavity/skin. Thus, it is very challenging to separate the lymphoma-specific alterations in the stroma from anatomic origin-specific alterations (Fig. 5, Suppl. Table 2). The authors have tried to exclude the anatomic variation by additional comparisons of lymphoma LN to only those 3 “homeostatic” LN which were of non-mesenteric origins. These data seem to be largely hidden in Suppl. Table 3, and reveal a high proportion of non-validated hits. This issue

should be elaborated more. The authors largely miss here a unique cell biological opportunity to unravel the differences in stromal cell compartments of peripheral and mesenteric LN in humans under "homeostatic" conditions (only DEGs listed in Suppl. Table 2). For instance, the stromal cells of these two LN systems are known to represent different leukocyte trafficking routes, they are differentially exposed to intestinal lipids and microbial products etc.
2. Identification of "transdifferentiating" BEC, LEC and non-EC stromal cell types (p.6, l8; p9, l13; p.10, l5; Fig. 2a, Fig.3a, Fig. 4a, Suppl. Fig. 4a) would be conceptually important. What is the evidence that they are not duplets formed after the final filtering steps during the sample preparation? Super-resolution microscopy showing the in situ existence of the claimed hybrid cell types using the best cell-type specific markers should be performed.
3. The identified specific markers should be exploited for prospective purification (FACS sort) of a given LN cell population for cell biological experiments. E.g. the transdifferentiating cells, tip-like (and Ly6H+) blood EC, ATF3 hi and low smooth muscle cells could be purified to validate their unique characteristics (now only speculated in the text) using selected proof-of-concept wet-lab experiments.
4. The proposed widespread lymphatic EC damage in follicular lymphoma (p. 13, l10; p15, l6) is interesting. Very minimally, it should be verified by immunostainings. In addition, Fig. 5d indicates induction of molecules involved in antigen presentation (HLA, CD74) in these lymphatic EC, which could be functionally validated (see 3.)
5. To validate the robustness of the in silico interactome analyses (Fig. 6), at least a representative novel interaction should be verified experimentally. For instance, ex vivo Stamper-Woodruff-type binding assays using frozen sections and lymphoma cells in conjunction with blocking antibodies could give support to relevance of the predicted interactions.
6. The prognostic implications of the identified stromal markers should be analyzed further. The selected markers should also be used as continuous variables and their value in stratifying the clinically challenging intermediate prognosis group should be tested. In addition, it has already been reported that angiogenesis is a poor prognostic marker in follicular lymphoma (e.g. PMID: 20713461, PMID: 20630741). Do the proposed novel markers bring any additional value in comparison to pan-endothelial markers (CD31, VE-cadherin, Plvap etc) in the current data set? Fig. 7c should include more patients and quantification.
7. A user-friendly web-interface for analyzing each stromal cell type in homeostatic and lymphoma LN (including a possibility to focus on peripheral and mesenteric LN separately) should be included for full exploitation of this resource data.

Minor concerns:

1. The "homeostatic" LN were obtained from cancer patients. Although they did not contain malignant cells detectable by FACS, they likely were draining the tumor area, and thus have been exposed for years to the tumor microenvironment. They thus may be activated rather than homeostatic LN (possibly explaining e.g. the activated blood EC phenotypes). Were the whole LN digested for sc-RNAseq

experiments, and what were the absolute numbers of blood, lymphatic and non-EC per LN (or per gram of LN)?
2. p.3, l3: plasma cells and dendritic cells are also leukocytes.
3. Fig. 3e is highly useful, but should include also data from PMID: 32251437 and PMID: 33333021. Similar comparative analyses between mouse and human blood EC and non-EC stromal cell types would be helpful.
4. Bridge-LECs are shown to constitute about 20% of LN total lymphatic ECs (Fig 3b). Immunostainings are needed to substantiate that these EC lining the minute cord-like transsinusoidal bridges are indeed as prevalent as floor and medullary LECs.
5. What is the numbering used for the citations in Suppl. text?

Reviewer #3:
Remarks to the Author:
The authors have developed a single cell atlas of non-hematopoietic lymph node cells in health and disease. They have generally used standard techniques in the field and have done a fairly careful analysis of the data. Specific points I would like to see addressed are:

1. The authors state in several places that their atlas is "comprehensive". This seems to me to be extremely difficult to prove. How do they know that they have not missed subtle or rare cell types? How do they know that they have not missed cell types which are not distinguishable based solely on transcriptome? They essentially walk back the claim of being comprehensive when discussing limitations towards the end of the paper and therefore they should not make it in the first place.

2. The authors state that they have developed and approach to handle "clinical samples often difficult to assay". They do not appear to me to have advanced the state of the art in this manner - there are many examples of this in the literature by now.

3. The reading is somewhat dry and the paper appears to be a laundry list of putative cell types- as the authors themselves point out, they have not done any work to functionally validate their claims. This seems to be an especially strong defect in the cell-communication analysis using cellphoneDB, which is entirely informatic and speculative.

4. It is by now well known that when using the 10x system there is RNA from lysed cells which cross-contaminates the droplets. There have been a few published examples of how to correct for this. The authors should either perform such a correction or show that it is not necessary.

5. The analysis of the pathological samples seems incomplete. What are the gene expression signatures that are clearly neoplastic, especially in the B cells? Can the authors identify genomic alterations related to cancer in their transcriptome data from those samples?

GUIDELINES FOR MANUSCRIPT SUBMISSION TO NATURE CELL BIOLOGY

READABILITY OF MANUSCRIPTS – Nature Cell Biology is read by cell biologists from diverse backgrounds, many of whom are not native English speakers. Authors should aim to communicate their findings clearly, explaining technical jargon that might be unfamiliar to non-specialists, and avoiding non-standard abbreviations. Titles and abstracts should concisely communicate the main findings of the study, and the background, rationale, results and conclusions should be clearly explained in the manuscript in a manner accessible to a broad cell biology audience. Nature Cell Biology uses British spelling.

MANUSCRIPT FORMAT – please follow the guidelines listed in our Guide to Authors regarding manuscript formats at Nature Cell Biology.

TITLE – should be no more than 100 characters including spaces, without punctuation and avoiding technical terms, abbreviations, and active verbs..

AUTHOR NAMES – should be given in full.

AUTHOR AFFILIATIONS – should be denoted with numerical superscripts (not symbols) preceding the names. Full addresses should be included, with US states in full and providing zip/post codes. The corresponding author is denoted by: "Correspondence should be addressed to [initials]."

ABSTRACT AND MAIN TEXT – please follow the guidelines that are specific to the format of your manuscript, as listed in our Guide to Authors (http://www.nature.com/ncb/pdf/ncb_gta.pdf) Briefly, Nature Cell Biology Articles, Resources and Technical Reports have 3500 words, including a 150 word abstract, and the main text is subdivided in Introduction, Results, and Discussion sections. Nature Cell

Biology Letters have up to 2500 words, including a 180 word introductory paragraph (abstract), and the text is not subdivided in sections.

ACKNOWLEDGEMENTS – should be kept brief. Professional titles and affiliations are unnecessary. Grant numbers can be listed.

AUTHOR CONTRIBUTIONS – must be included after the Acknowledgements, detailing the contributions of each author to the paper (e.g. experimental work, project planning, data analysis etc.). Each author should be listed by his/her initials.

FINANCIAL AND NON-FINANCIAL COMPETING INTERESTS – the authors must include one of three declarations: (1) that they have no financial and non-financial competing interests; (2) that they have financial and non-financial competing interests; or (3) that they decline to respond, after the Author Contributions section. This statement will be published with the article, and in cases where financial and non-financial competing interests are declared, these will be itemized in a web supplement to the article. For further details please see https://www.nature.com/licenceforms/nrg/competing-interests.pdf.

REFERENCES – are limited to a total of 70 for Articles, Resources, Technical Reports; and 40 for Letters. This includes references in the main text and Methods combined. References must be numbered sequentially as they appear in the main text, tables and figure legends and Methods and must follow the precise style of Nature Cell Biology references. References only cited in the Methods should be numbered consecutively following the last reference cited in the main text. References only associated with Supplementary Information (e.g. in supplementary legends) do not count toward the total reference limit and do not need to be cited in numerical continuity with references in the main text. Only published papers can be cited, and each publication cited should be included in the numbered reference list, which should include the manuscript titles. Footnotes are not permitted.

METHODS – Nature Cell Biology publishes methods online. The methods section should be provided as a separate Word document, which will be copyedited and appended to the manuscript PDF, and incorporated within the HTML format of the paper.

Methods should be written concisely, but should contain all elements necessary to allow interpretation and replication of the results. As a guideline, Methods sections typically do not exceed 3,000 words. The Methods should be divided into subsections listing reagents and techniques. When citing previous methods, accurate references should be provided and any alterations should be noted. Information must be provided about: antibody dilutions, company names, catalogue numbers and clone numbers for monoclonal antibodies; sequences of RNAi and cDNA probes/primers or company names and catalogue numbers if reagents are commercial; cell line names, sources and information on cell line identity and

authentication. Animal studies and experiments involving human subjects must be reported in detail, identifying the committees approving the protocols. For studies involving human subjects/samples, a statement must be included confirming that informed consent was obtained. Statistical analyses and information on the reproducibility of experimental results should be provided in a section titled "Statistics and Reproducibility".

All Nature Cell Biology manuscripts submitted on or after March 21 2016 must include a Data availability statement at the end of the Methods section. For Springer Nature policies on data availability see http://www.nature.com/authors/policies/availability.html; for more information on this particular policy see http://www.nature.com/authors/policies/data/data-availability-statements-data-citations.pdf. The Data availability statement should include:

• Accession codes for primary datasets (generated during the study under consideration and designated as "primary accessions") and secondary datasets (published datasets reanalysed during the study under consideration, designated as "referenced accessions"). For primary accessions data should be made public to coincide with publication of the manuscript. A list of data types for which submission to community-endorsed public repositories is mandated (including sequence, structure, microarray, deep sequencing data) can be found here http://www.nature.com/authors/policies/availability.html#data.

• Unique identifiers (accession codes, DOIs or other unique persistent identifier) and hyperlinks for datasets deposited in an approved repository, but for which data deposition is not mandated (see here for details http://www.nature.com/sdata/data-policies/repositories).

• At a minimum, please include a statement confirming that all relevant data are available from the authors, and/or are included with the manuscript (e.g. as source data or supplementary information), listing which data are included (e.g. by figure panels and data types) and mentioning any restrictions on availability.

• If a dataset has a Digital Object Identifier (DOI) as its unique identifier, we strongly encourage including this in the Reference list and citing the dataset in the Methods.

We recommend that you upload the step-by-step protocols used in this manuscript to the Protocol Exchange. More details can found at www.nature.com/protocolexchange/about.

DISPLAY ITEMS – main display items are limited to 6-8 main figures and/or main tables for Articles, Resources, Technical Reports; and 5 main figures and/or main tables for Letters. For Supplementary Information see below.

FIGURES – Colour figure publication costs $600 for the first, and $300 for each subsequent colour figure. All panels of a multi-panel figure must be logically connected and arranged as they would appear in the final version. Unnecessary figures and figure panels should be avoided (e.g. data presented in small tables could be stated briefly in the text instead).

All imaging data should be accompanied by scale bars, which should be defined in the legend. Cropped images of gels/blots are acceptable, but need to be accompanied by size markers, and to retain visible background signal within the linear range (i.e. should not be saturated). The boundaries of panels with low background have to be demarked with black lines. Splicing of panels should only be considered if unavoidable, and must be clearly marked on the figure, and noted in the legend with a statement on whether the samples were obtained and processed simultaneously. Quantitative comparisons between samples on different gels/blots are discouraged; if this is unavoidable, it should only be performed for samples derived from the same experiment with gels/blots were processed in parallel, which needs to be stated in the legend.

Figures should be provided at approximately the size that they are to be printed at (single column is 86 mm, double column is 170 mm) and should not exceed an A4 page (8.5 x 11"). Reduction to the scale that will be used on the page is not necessary, but multi-panel figures should be sized so that the whole figure can be reduced by the same amount at the smallest size at which essential details in each panel are visible. In the interest of our colour-blind readers we ask that you avoid using red and green for contrast in figures. Replacing red with magenta and green with turquoise are two possible colour-safe alternatives. Lines with widths of less than 1 point should be avoided. Sans serif typefaces, such as Helvetica (preferred) or Arial should be used. All text that forms part of a figure should be rewritable and removable.

We accept files from the following graphics packages in either PC or Macintosh format:

- For line art, graphs, charts and schematics we prefer Adobe Illustrator (.AI), Encapsulated PostScript (.EPS) or Portable Document Format (.PDF). Files should be saved or exported as such directly from the application in which they were made, to allow us to restyle them according to our journal house style.

- We accept PowerPoint (.PPT) files if they are fully editable. However, please refrain from adding PowerPoint graphical effects to objects, as this results in them outputting poor quality raster art. Text used for PowerPoint figures should be Helvetica (preferred) or Arial.

- We do not recommend using Adobe Photoshop for designing figures, but we can accept Photoshop generated (.PSD or .TIFF) files only if each element included in the figure (text, labels, pictures, graphs, arrows and scale bars) are on separate layers. All text should be editable in ‘type layers’ and line-art

such as graphs and other simple schematics should be preserved and embedded within 'vector smart objects' - not flattened raster/bitmap graphics.

- Some programs can generate Postscript by 'printing to file' (found in the Print dialogue). If using an application not listed above, save the file in PostScript format or email our Art Editor, Allen Beattie for advice (a.beattie@nature.com).

Regardless of format, all figures must be vector graphic compatible files, not supplied in a flattened raster/bitmap graphics format, but should be fully editable, allowing us to highlight/copy/paste all text and move individual parts of the figures (i.e. arrows, lines, x and y axes, graphs, tick marks, scale bars etc.). The only parts of the figure that should be in pixel raster/bitmap format are photographic images or 3D rendered graphics/complex technical illustrations.

All placed images (i.e. a photo incorporated into a figure) should be on a separate layer and independent from any superimposed scale bars or text. Individual photographic images must be a minimum of 300+ DPI (at actual size) or kept constant from the original picture acquisition and not decreased in resolution post image acquisition. All colour artwork should be RGB format.

FIGURE LEGENDS – must not exceed 350 words for each figure to allow fit on a single printed NCB page together with the figure. They must include a brief title for the whole figure, and short descriptions of each panel with definitions of the symbols used, but without detailing methodology.

TABLES – main tables should be provided as individual Word files, together with a brief title and legend. For supplementary tables see below.

SUPPLEMENTARY INFORMATION – Supplementary information is material directly relevant to the conclusion of a paper, but which cannot be included in the printed version in order to keep the manuscript concise and accessible to the general reader. Supplementary information is an integral part of a Nature Cell Biology publication and should be prepared and presented with as much care as the main display item, but it must not include non-essential data or text, which may be removed at the editor's discretion. All supplementary material is fully peer-reviewed and published online as part of the HTML version of the manuscript. Supplementary Figures and Supplementary Notes are appended at the end of the main PDF of the published manuscript.

Supplementary items should relate to a main text figure, wherever possible, and should be mentioned sequentially in the main manuscript, designated as Supplementary Figure, Table, Video, or Note, and

numbered continuously (e.g. Supplementary Figure 1, Supplementary Figure 2, Supplementary Table 1, Supplementary Table 2 etc.).

Unprocessed scans of all key data generated through electrophoretic separation techniques need to be presented in a supplementary figure that should be labelled and numbered as the final supplementary figure, and should be mentioned in every relevant figure legend. This figure does not count towards the total number of figures and is the only figure that can be displayed over multiple pages, but should be provided as a single file, in PDF or TIFF format. Data in this figure can be displayed in a relatively informal style, but size markers and the figures panels corresponding to the presented data must be indicated.

The total number of Supplementary Figures (not including the “unprocessed scans” Supplementary Figure) should not exceed the number of main display items (figures and/or tables (see our Guide to Authors and March 2012 editorial http://www.nature.com/ncb/authors/submit/index.html#suppinfo; http://www.nature.com/ncb/journal/v14/n3/index.html#ed). No restrictions apply to Supplementary Tables or Videos, but we advise authors to be selective in including supplemental data.

Each Supplementary Figure should be provided as a single page and as an individual file in one of our accepted figure formats and should be presented according to our figure guidelines (see above). Supplementary Tables should be provided as individual Excel files. Supplementary Videos should be provided as .avi or .mov files up to 50 MB in size. Supplementary Figures, Tables and Videos much be accompanied by a separate Word document including titles and legends.

GUIDELINES FOR EXPERIMENTAL AND STATISTICAL REPORTING

REPORTING REQUIREMENTS – To improve the quality of methods and statistics reporting in our papers we have recently revised the reporting checklist we introduced in 2013. We are now asking all life sciences authors to complete two items: an Editorial Policy Checklist (found here https://www.nature.com/authors/policies/Policy.pdf) that verifies compliance with all required editorial policies and a reporting summary (found here https://www.nature.com/authors/policies/ReportingSummary.pdf) that collects information on experimental design and reagents. These documents are available to referees to aid the evaluation of the manuscript. Please note that these forms are dynamic ‘smart pdfs’ and must therefore be downloaded and completed in Adobe Reader. We will then flatten them for ease of use by the reviewers. If you would like to reference the guidance text as you complete the template, please access these flattened versions at http://www.nature.com/authors/policies/availability.html.

STATISTICS – Wherever statistics have been derived the legend needs to provide the n number (i.e. the sample size used to derive statistics) as a precise value (not a range), and define what this value

represents. Error bars need to be defined in the legends (e.g. SD, SEM) together with a measure of centre (e.g. mean, median). Box plots need to be defined in terms of minima, maxima, centre, and percentiles. Ranges are more appropriate than standard errors for small data sets. Wherever statistical significance has been derived, precise p values need to be provided and the statistical test used needs to be stated in the legend. Statistics such as error bars must not be derived from n<3. For sample sizes of n<5 please plot the individual data points rather than providing bar graphs. Deriving statistics from technical replicate samples, rather than biological replicates is strongly discouraged. Wherever statistical significance has been derived, precise p values need to be provided and the statistical test stated in the legend.

Information on how many times each experiment was repeated independently with similar results needs to be provided in the legends and/or Methods for all experiments, and in particular wherever representative experiments are shown.

We strongly recommend the presentation of source data for graphical and statistical analyses as a separate Supplementary Table, and request that source data for all independent repeats are provided when representative experiments of multiple independent repeats, or averages of two independent experiments are presented. This supplementary table should be in Excel format, with data for different figures provided as different sheets within a single Excel file. It should be labelled and numbered as one of the supplementary tables, titled “Statistics Source Data”, and mentioned in all relevant figure legends.

--------- Please don't hesitate to contact NCB@nature.com should you have queries about any of the above requirements ---------

Author Rebuttal to Initial comments

Responses to the Reviewers' comments

NCB-A45004A: "A single-cell atlas of non-haematopoietic cells in human lymph nodes and lymphoma reveals a landscape of stromal remodelling"

Responses to comments of Reviewer #1:

1) **A major issue for this paper is the framing of the comparator lymph nodes as 'healthy'. The lymph nodes referred to throughout the paper as 'homeostatic lymph nodes' or HLN are in fact not from healthy subjects. On the contrary, they are lymph nodes from patients with a variety of solid cancers and rather disconcertingly this was not presented upfront. Thus, these comparator lymph nodes should not be considered 'homeostatic' unless the authors can provide evidence that the LNs from solid-tumor bearing patients are in fact equivalent to LNs from healthy subjects or non-reactive. The paper would benefit immensely from having comparison data derived from truly 'homeostatic' LNs. Without these data the manuscript title, text and conclusions will need to be modified to reflect the nature of the LNs being used as comparators with the lymphoma samples.**

Response:

Thank you for pointing out the need for clarity on this important issue; the Reviewer's concerns are quite understandable. This is an inevitable limitation of studies that analyse human lymph nodes (LNs), particularly those that use raw LN samples. We described this point as a study limitation in the Discussion section (page #23, lines #389–390). As the Reviewer suggested, LN samples used in our study might not have been completely in homeostatic status, and we thus changed the description of these LN samples from "homeostatic LN (HLN)" to "metastasis-free LN (MFLN)" throughout the manuscript.

In a recent single-cell analysis of human LN lymphatic endothelial cells (LECs) (Takeda et al., *Immunity*, 2019. PMID: 31402260), the authors used non-sentinel LNs (distant from tumours) collected from patients with cancer and concluded that this approach could minimize the possible influence of tumour-derived factors on LN non-haematopoietic cells. Therefore, in the present study, we similarly used non-sentinel LNs without enlargement (>5mm) or cancer metastasis detected by using flow cytometry (Extended Data Fig. 1b). We added representative macroscopic images of MFLN and FL samples (Extended Data Fig. 1a).

Fortunately, our cohort included one patient who was diagnosed with a benign thyroid tumour (MFLN #8). This sample was putatively more similar to a healthy LN than were the other MFLN samples. We thus analysed stromal cells from MFLN #8 separately and compared the data with that obtained from the other MFLN samples. Thus, we found that stromal cells from MFLN #8 were clustered highly similarly to cells from the other

samples in unsupervised clustering analysis. We also found that most of top differentially expressed genes (DEGs) for each stromal cell subcluster detected in all MFLN data were shared with DEGs calculated from MFLN #8. These data collectively suggest a minimal to negligible influence of malignancy-derived factors on our single-cell atlas. We added these data and comments in Supplementary information (Supplementary Notes page #8, lines #122–127, Extended Data Fig. 5a–c) and the Discussion section (page #21, lines #347–352).

2) **In the abstract the authors refer to 27 human samples however only 19 samples (NHL and FL) were deeply analyzed. The DLBCL and PTCL samples may have been analyzed but were not used as comparators with the FL or characterized as deeply.**

Response:
Thank you for this comment. As the Reviewer recommended, we added data from sub-clustering analyses of PTCL and tDLBCL NHCs and adjusted proportions of each NHC subcluster in comparison with MFLN NHCs. In addition, we performed DEG analysis of tDLBCL subclusters and trajectory analysis in tDLBCL NESCs. We could clearly detect NHC subclusters in both PTCL and tDLBCL NHCs by clustering analysis using the single-cell NHC atlas. Furthermore, we found that the tendency of changes in cell proportions observed in FL NHCs was intensified in tDLBCL NHCs, particularly in NESC subclusters, likely presenting a terminal form of FL stromal remodelling. Consistently, trajectory analysis suggested that some NESC subclusters had differentiated into follicular stromal cells. We also observed that some DEGs detected in FL subclusters were also upregulated in tDLBCL NHCs. We added these findings in the manuscripts and figures (pages #19–21, lines #319–340, Extended Data Fig. 10a–j). These results demonstrate the applicability of our single-cell atlas to other lymphoma types, support the reliability of findings in FL stroma, and provide insights into stromal alterations from FL transforming into tDLBCL.

3) **On line 12 the authors refer to BAFF, IL-15 and HGF as FDC-derived molecules. Can the authors analyze the expression of these genes across the NHC subtypes in their scRNAseq data and comment on the pattern? This will help the readers understand which single NHC type in human LN and FL express the genes encoding these molecules.**

Response:
Thank you for this comment. As the Reviewer suggested, we analysed the expression of key genes mentioned in the manuscript (*BAFF*, *IL15*, *HGF*, *CXCL13*, *POSTN*, *FAP*, and

EGFL6) across NHC subclusters and found that these genes were varyingly upregulated at subcluster levels. We added these results in the figures (Extended Data Fig. 7b).

4) **On page 5 and in Figure 1f and Suppl. Figure 1f the authors reference flow cytometry analysis to validate the proportions of LECs and BECs. Can the authors comment on the flow cytometry data for NESC (PDPN+ and PDPN-)? Are the proportions of NESC concordant with the scRNAseq data?**

Response:
Thank you for this comment. As the Reviewer indicated, we checked the correlation between flow cytometry and scRNAseq analyses for NESC proportions as performed for BECs and LECs, and found an excellent concordance in NESC proportion between the two methods. We added these results to the manuscripts and figures (page #5, lines #63–64, Fig. 1e; Extended Data Fig. 1h).

5) **The data in Figure 5 (and in many figures) lack statistical testing to clarify whether differences in proportions are significant. Where proportions increase or decrease the authors should also comment on the concomitant changes.**

Response:
Thank you for this comment. We added statistical testing for comparisons of cell proportions (Fig. 5a; Extended Data Fig. 10d, 10f) and DEG analyses (Fig. 5c; Extended Data Fig. 6a, 7b). We also added comments on the concomitant changes (page #13, lines #204–208; page #20, lines #327–334).

6) **The font in many figures is too small. For example, the data and labels in Figure 5d are much too small. One should not need to zoom in to see every subfigure. This figure could be improved by generating heatmaps that show each patient sample.**

Response:
Thank you for this comment. We improved Figure 5d (Fig. 5c in the revised manuscript) by making the font bigger. We also made maximum effort to make the font bigger in all other figures. We tried to generate heatmaps according to the Reviewer's recommendation. Unfortunately, DEGs upregulated in FL subclusters were not more clearly visible by heatmaps than current violin plots, presumably because the atlas includes many subclusters and samples.

7) **Can the authors explain why they chose the genes shown in Figure 5d? Figure 5d would be strengthened and more informative by showing the expression of all genes across each of the cell subtypes. All key genes referenced on page 14 should be shown in a main figure (Cxcl13, Postn, Fap, Egfl6 across cell subtypes.**

Response:
Thank you for this comment. We apologize for not explaining clearly regarding Fig. 5d (Fig. 5c in the revised manuscript). In this Figure, we selected the "top 3" DEGs, ranked according to the log fold change. As mentioned in our response to the editor's comment C (C-2), we analysed the expression of key genes mentioned in the manuscript (*CXCL13*, *POSTN*, *FAP*, and *EGFL6*) across NHC subclusters and found that these genes were variously upregulated at subcluster levels. We added figures for showing these results, although they were included in Extended Data owing to the count limit of main figures (Extended Data Fig. 7b).

8) **Can the authors provide data on the total cellularity, mass and size or volume of the lymph nodes used in the study and provide information on the numbers of each NHC subtype?**

Response:
Thank you for this comment. We added data on the number of total cells analysed using scRNAseq analysis (Extended Data Fig. 1c). We also added information on the proportions of each NHC subtype (Extended Data Fig. 5a).

9) **On page 14, the authors discuss gene expression related to lymphocyte migration and draw conclusions about the cell types providing the relevant cues. The data in Suppl. Figure 5 is potentially interesting. What are the gene differences between the GO categories: 'positive regulation of leukocyte chemotaxis', 'mononuclear cell migration', and 'regulation of lymphocyte migration' to help explain the differences between TRC and MRC?**

Response:
Thank you for this comment. Each GO term for a given subcluster includes different genes upregulated in the subcluster. We thus added data from the results of GO analysis that include information of these genes (Supplemental Table 2e, 2j, 3b, 4d). For example, the GO terms "mononuclear cell migration" and "positive regulation of leukocyte chemotaxis" for FL TRCs included *CCL26*, *CXCL10*, and *IL34* and *CXCL10*, *PGF*, and *IL34*,

respectively, whereas “regulation of lymphocyte migration” for FL MRCs included *CXCL13*, *CXCL14*, and *IL27RA*. Newly added supplemental data will help the readers to understand that these different genes underlie different GO terms.

10) On lines 6-7, page 15 the authors refer to widespread lymphatic damage to account for the significant decrease in LECs in FL. This statement needs to be substantiated by imaging data.

Response:
Thank you for this comment. As the Reviewer suggested, we performed IF staining of LECs and found that, consistent with the results of single-cell analysis, LECs were decreased in FL samples compared with than in MFLN samples (page #15, lines #241–242, Extended Data Fig. 7d, 7e).

11) The authors may want to further clarify the significance of the analysis in Supplementary Figure 8.

Response:
Thank you for this comment. As mentioned earlier, we added data from sub-clustering analyses of PTCL and tDLBCL NHCs and alterations of proportions of each NHC subcluster in comparison with MFLN NHCs. In addition, we performed DEG analysis of tDLBCL subclusters and trajectory analysis in tDLBCL NESCs (please see our response to your comment 2).

12) On page 17 and in other places in the manuscript the authors refer to FDCs as key sources of Cxcl13 and yet the data in this manuscript do not support these conclusions. In fact, their data showed that Cxcl13 expression is extremely low in FDCs whereas MRCs express relatively high levels.

Response:
Thank you for this comment. We apologize for misleading the Reviewer by not explaining this point clearly. In both MFLN and FL samples, *CXCL13* expression was higher in FDCs than in MRCs (Fig. 4d, Extended Data Fig. 7b). We added violin plots showing *CXCL13* expressions of FL subclusters in Extended Data Fig. 7b. *TNFSF13B* was found to be highly expressed by MRCs. This point is described in the revised manuscript (pages #10–11, lines #160–162, Fig. 4e).

13) **The data in Figure 7c is potentially interesting. The authors need to provide quantitation for each sample and across patients including statistical analysis. LOX and LY6H expression overlaps with PLVAP and MECA79 in some cells but numerous single positive (red) cells are evident in the FL samples but not the NHL. What are these cells?**

Response:

Thank you for this comment. We performed IF staining of markers proposed in Fig. 7c in multiple MFLN and FL samples and validated their upregulation in FL samples via quantification with statistical testing (Fig. 7c). LY6H- and LOX-positive cells were mostly positive for PLVAP, though there is a possibility that LY6H and LOX were expressed by other NHC types. Our single-cell data showed that expression of *PLVAP* was lower in tip cells (tBECs) compared with cBECs (shown below). Indeed, PLVAP-expression was frequently weaker in tBECs than in cBECs in the IF staining results, and this is one of the reasons why tip cells appear as a single positive. We therefore changed the colour of PLVAP from white to green to make it easy to recognize double positive cells. In addition, we changed LY6H and LOX images to more typical ones (Fig. 7c).

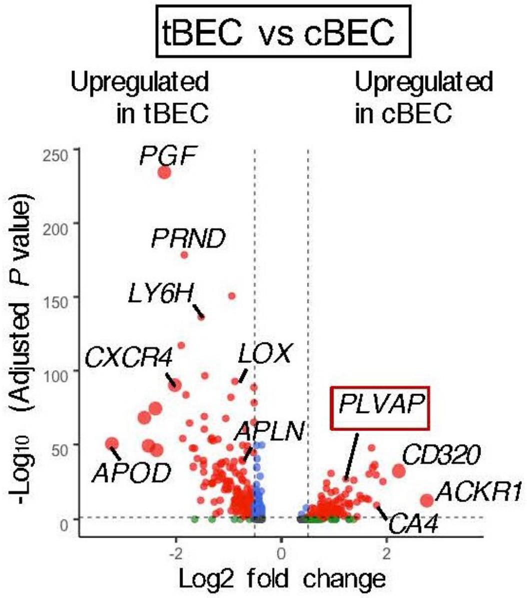

Figure legend: Volcano plot of up- or down-regulated genes between tBECs and cBECs

14) **In Figure 7c, the TDO2 and REM1 staining is clearly not overlapping with CD31 but no marker for TRC was used to confirm the identity of these cells. This should be addressed with staining for a TRC marker.**

Response:

Thank you for this comment. As suggested, we performed IF staining of TDO2 and REM1 in combination with TRC marker CCL19 and found that many CCL19-positive TRCs were positive for TDO2 and REM1 in FL samples (Fig. 7c). Since REM1 was abundantly expressed by PCs and SMCs, REM1 positivity was quantified by calculating the REM1-positive area (Fig. 7c)

15) **The survival analysis could be strengthened by examining gene signatures for each cell subset. For example, would a signature for tip cells be associated with reduced survival similar to LY6H and LOX? Likewise for TRC?**

Response:

Thank you for this comment. As the Reviewer indicated, we tested the prognostic impact of TRC signatures by detecting genes that were found to be specifically upregulated in FL TRCs (*EDNRA*, *RASL12*, *ENPEP*, *HIGD1B*, *EGFL6*, *CBLN1*, *TDO2*, *WISP1*, *HTR1F*, *PCDH11X*, and *DLX5*), and found that the prognostic impact of upregulation of TRC signature was not statistically significant (shown below). This indicates that the high proportion of TRCs in lymphoma stroma may not necessarily be related to patient prognosis, but that particular changes in TRC gene expression profiles may have a more direct impact on patient prognosis. Unfortunately, the number of genes specifically expressed by tip cells and upregulated in FL tip cells was too small to create a definite tip cell signature in FL samples.

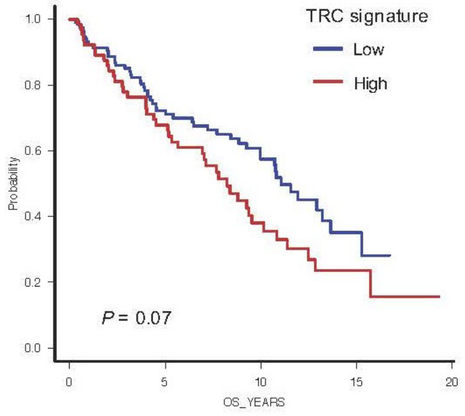

Figure legend: Overall survival of FL patients according to the TRC signature

16) **Are LY6H, LOX, REM1 and TDO2 poor prognostic factors in DLBCL and PTCL?**

Response:

Thank you for this comment. We used a microarray dataset of DLBCL (Lenz et al., *N Engl J Med*, 2008. PMID: 19038878) for prognostic analysis. DLBCL is a relatively heterogeneous lymphoma subtype with varying cell origins and clinical and prognostic features. We found that upregulation of these genes affected prognostic impact according to different DLBCL origin cells (shown below). As we mentioned earlier, we added analysis of PTCL and tDLBCL stroma. However, sample sizes of these lymphoma cohorts were too small to expand to further analyses, including prognostic analysis. We would like to accumulate data of DLBCL stroma and analyse prognostic relevance of alterations to stroma-derived gene expression profiles in future studies.

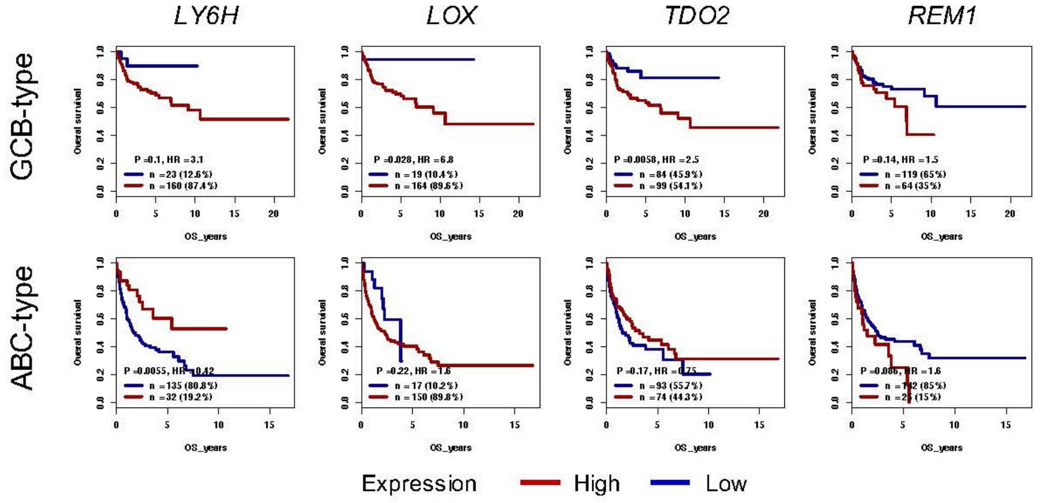

Figure legend: Overall survival of DLBCL patients stratified by *LY6H*, *LOX*, *TDO2*, and *REM1* expression according to DLBCL cell of origins (GCB- or ABC-type)

17) **Statistical tests and appropriate values should be included in all relevant figures and clarified in the text or legends.**

Response:

Thank you for this comment. As suggested, we added statistical tests to comparisons of cell proportions (Fig. 5a; Extended Data Fig. 10d, 10f) and differentially expressed gene analyses (Fig. 5c; Extended Data Fig. 6a, 7b).

18) **The number of single cells analyzed in each UMAP should be provided in the figures or in the figure legends.**

Response:

Thank you for this comment. As suggested, we described the number of cells analysed in each UMAP in each figure.

Responses to comments of Reviewer #2:

1) **The homeostatic LNs were mainly from mesenteric LN draining the gut, while the lymphoma samples were from peripheral (mainly cervical) LN draining the oral cavity/skin. Thus, it is very challenging to separate the lymphoma-specific alterations in the stroma from anatomic origin-specific alterations (Fig. 5, Suppl. Table 2). The authors have tried to exclude the anatomic variation by additional comparisons of lymphoma LN to only those 3 "homeostatic" LN which were of non-mesenteric origins. These data seem to be largely hidden in Suppl. Table 3, and reveal a high proportion of non-validated hits. This issue should be elaborated more. The authors largely miss here a unique cell biological opportunity to unravel the differences in stromal cell compartments of peripheral and mesenteric LN in humans under "homeostatic" conditions (only DEGs listed in Suppl. Table 2). For instance, the stromal cells of these two LN systems are known to represent different leukocyte trafficking routes, they are differentially exposed to intestinal lipids and microbial products etc.**

   Response:
   Thank you for this insightful comment. As suggested, we analysed anatomic origin-specific alterations using metastasis-free LN samples. We found that high endothelial venule subclusters in mesenteric LNs highly expressed *MADCAM1* compared with that in peripheral LNs, consistent with previous reports showing different leukocyte trafficking manners (von Andrian et al., *Nat Rev Immunol*, 2003. PMID: 14668803). We also found that genes associated with inflammatory response or response to bacterial molecules were highly enriched in NESC subclusters of mesenteric LN samples compared with those of peripheral LN, particularly in TNF-SCs. We described these findings in Supplementary information (Supplementary Notes pages #8–9, lines #128–142, Extended Data Fig. 6a, 6b).

2) **Identification of "transdifferentiating" BEC, LEC and non-EC stromal cell types (p.6, l8; p9, l13; p.10, l5; Fig. 2a, Fig.3a, Fig. 4a, Suppl. Fig. 4a) would be conceptually important. What is the evidence that they are not duplets formed after the final filtering steps during the sample preparation? Super-resolution microscopy showing the in situ existence of the claimed hybrid cell types using the best cell-type specific markers should be performed.**

   Response:
   Thank you for pointing out this issue. As suggested, we re-analysed these subclusters and found that the average amount of mRNA in these cells were higher than in other cell types.

Although previously suggested transdifferentiating cells may have been included in our data, our current analytic technology could not precisely discriminate them. Also, IF staining could not clearly confirm the existence of these hybrid cell types. Therefore, in this study, we excluded these cells as potentially being doublets (page #29, lines #483–485). We believe that this modification strengthens the revised single-cell NHC atlas, and we would like to investigate the existence and characteristics of these hybrid cells in the future studies.

3) **The identified specific markers should be exploited for prospective purification (FACS sort) of a given LN cell population for cell biological experiments. E.g. the transdifferentiating cells, tip-like (and Ly6H+) blood EC, ATF3 hi and low smooth muscle cells could be purified to validate their unique characteristics (now only speculated in the text) using selected proof-of-concept wet-lab experiments.**

Response:
Thank you for this comment. Unfortunately, we could not successfully purify the indicated NHC subclusters. We found no specific surface markers for SMC subclusters that could be used to discriminate them from each other. Also, the number of tip cells was too small to precisely purify from the limited amount of human LN samples. Therefore, we added IF staining of PGF to confirm that rare PGF-positive BECs (tBECs) were consistently located at the tips of capillary BECs (Extended Data Fig. 2g). We also added IF staining of HSP70 in SMCs to support the results of DEG analysis between SMC subclusters (Fig. 4f). We found that ATF3-positive SMCs concomitantly express HSP70, suggesting that $ATF3^{hi}$ SMCs may be damaged by mechanical stimuli such as blood flow and/or immune cell trafficking (Xu et al., Circ Res, 2000. PMID: 10850962) (page #12, lines #193–195, Extended Data Fig. 4m).

4) **The proposed widespread lymphatic EC damage in follicular lymphoma (p. 13, l10; p15, l6) is interesting. Very minimally, it should be verified by immunostainings. In addition, Fig. 5d indicates induction of molecules involved in antigen presentation (HLA, CD74) in these lymphatic EC, which could be functionally validated (see 3.) .**

Response:
Thank you for this comment. As the Reviewer indicated, we performed IF staining to detect LECs using FL samples. We found that LEC density was significantly decreased in FL compared with that in MFLN samples (page #15, lines #241–242, Extended Data Fig. 7d, 7e). We also found that the proportion of CD74-positive LECs among all LECs was significantly higher in FL samples (page #15, lines #244–245, Extended Data Fig. 7f, 7g).

Unfortunately, we could not collect sufficient raw FL samples to extract LECs for functional experiments.

5) **To validate the robustness of the in silico interactome analyses (Fig. 6), at least a representative novel interaction should be verified experimentally. For instance, ex vivo Stamper-Woodruff-type binding assays using frozen sections and lymphoma cells in conjunction with blocking antibodies could give support to relevance of the predicted interactions.**

Response:
Thank you for this constructive comment. As suggested, based on the results of *CellPhoneDB* analysis, we further sought to explore an interaction that could potentially be targeted in lymphoma research. We carefully surveyed all candidate interactions proposed by *CellPhoneDB* interactome analysis from the viewpoint of novelty in the lymphoma field, as well as availability of validated reagents for functional assays. We noted that among candidate interactions, the CD70–CD27 interaction has recently been attracting attention in cancer research in the context of interplays across immune cells and cancer cells, whereas the roles of stroma-derived CD70 have scarcely been analysed. We also found a well-validated function-blocking antibody against the ligand of CD70 (CD27) (R & D systems), although no blocking antibody against CD70 was available. We thus performed binding assay experiments to validate the CD70–CD27 interaction across FL stroma and malignant B cells. We found that CD70 and CD27 were upregulated in FL medullary/adventitia stromal cells and malignant B cells, respectively. Then, we confirmed binding of malignant B cells to CD70 using *in vitro* binding assays. The binding was inhibited by a function-blocking antibody against CD27. As the Reviewer indicated, we also performed *ex vivo* adhesion assays using frozen FL sections and cryopreserved FL cells in conjunction with anti-CD27 antibody and found that FL cells binding to medullary lesions were inhibited by anti-CD27 antibody. These results are described in the Results section (pages #17–18, lines #279–301, Fig. 6a–g; Extended Data Fig. 8e–g). We also added comments regarding these results in the Discussion section (pages #22–23, lines #371–379). These findings collectively support the resource value of our single-cell analysis and provide novel insight into lymphoma stroma, suggesting a potential therapeutic target of FL.

6) **The prognostic implications of the identified stromal markers should be analyzed further. The selected markers should also be used as continuous variables and their value in stratifying the clinically challenging intermediate prognosis group should be tested. In addition, it has already been reported that angiogenesis is a poor prognostic**

marker in follicular lymphoma (e.g. PMID: 20713461, PMID: 20630741). Do the proposed novel markers bring any additional value in comparison to pan-endothelial markers (CD31, VE-cadherin, Plvap etc) in the current data set? Fig. 7c should include more patients and quantification.

Response:
Thank you for this comment. As suggested, we performed additional prognostic analysis using an intermediate prognosis group and found that patients with higher expressions of *LY6H*, *LOX*, and *TDO2* had a tendency to experience shorter overall survival (shown below). In the dataset used in the present study, high expressions of *PECAM1* (encoding CD31) and *CDH5* (encoding VE-cadherin) did not show unfavourable prognostic impact (shown below). We performed immunofluorescence staining of markers shown in Fig. 7c in other samples, and performed quantitative analysis of these markers (page #19, lines #315–317, Fig. 7c).

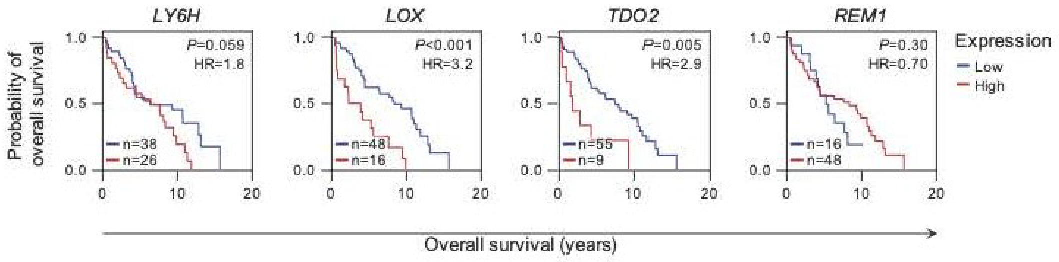

Figure legend: Overall survival of intermediate-risk FL patients (n = 64) defined by IPI based on expression of *LY6H*, *LOX*, *TDO2*, and *REM1*. HR, hazard ratio.

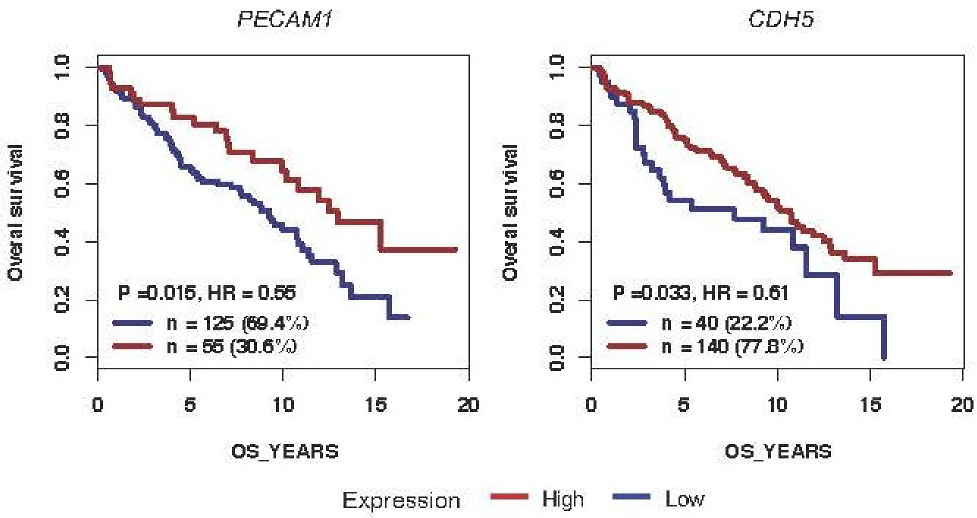

Figure legend: Overall survival of FL patients according to *PECAM1* and *CDH5* expressions

7) **A user-friendly web-interface for analyzing each stromal cell type in homeostatic and lymphoma LN (including a possibility to focus on peripheral and mesenteric LN separately) should be included for full exploitation of this resource data.**

Response:

Thank you for this suggestion. We have provided detailed results of differentially expressed genes for all NHC subclusters (Supplementary Table 2). We also submit our single-cell data to the European Genome-Phenome Archive (https://ega-archive.org). This will allow data-users to easily analyse the characteristics of NHCs or integrate the same with the users' single-cell data. Unfortunately, we were unable to develop the indicated web-interface.

8) **The "homeostatic" LN were obtained from cancer patients. Although they did not contain malignant cells detectable by FACS, they likely were draining the tumor area, and thus have been exposed for years to the tumor microenvironment. They thus may be activated rather than homeostatic LN (possibly explaining e.g. the activated blood EC phenotypes). Were the whole LN digested for sc-RNAseq experiments, and what were the absolute numbers of blood, lymphatic and non-EC per LN (or per gram of LN)?**

Response:

Thank you for pointing out the need for clarity on this important issue; the Reviewer's concerns are quite understandable. This is an inevitable limitation of studies that analyse human lymph nodes (LNs), particularly those that use raw LN samples. We described this point as a study limitation in the Discussion section (page #23, lines #389–390). As the Reviewer suggested, LN samples used in our study might not have been completely in homeostatic status, and we thus changed the description of these LN samples from "homeostatic LN (HLN)" to "metastasis-free LN (MFLN)" throughout the manuscript.

In a recent single-cell analysis of human LN lymphatic endothelial cells (LECs) (Takeda et al., *Immunity*, 2019. PMID: 31402260), the authors used non-sentinel LNs (distant from tumours) collected from patients with cancer and concluded that this approach could minimize the possible influence of tumour-derived factors on LN non-haematopoietic cells. Therefore, in the present study, we similarly used non-sentinel LNs without enlargement (>5mm) or cancer metastasis detected by flow cytometry (Extended Data Fig. 1b). We added representative macroscopic images of MFLN and FL samples (Extended Data Fig. 1a).

Fortunately, our cohort included one patient who was diagnosed with a benign thyroid tumour (MFLN #8). This sample was putatively more similar to a healthy sample than were the other MFLN samples. We thus analysed stromal cells from MFLN #8

separately and compared the data with that obtained from the other MFLN samples. Thus, we found that stromal cells from MFLN #8 were clustered highly similarly to cells from the other samples in unsupervised clustering analysis. We also found that most of top DEGs for each stromal cell subcluster detected in all MFLN data were shared with DEGs calculated from MFLN #8. These data collectively suggest a minimal influence of malignancy-derived factors on our single-cell atlas. We added these data and comments in Supplementary information (Supplementary Notes page #8, lines #122–127, Extended Data Fig. 5a–c) and the Discussion section (page #21, lines #347–352).

In sample collection for the present study, LN and lymphoma samples were divided into two parts: one for clinical examination for the diagnosis of cancer metastasis or lymphoma, and the other for the present study. After preparing the cell suspension, we performed mechanistic and enzymatic digestion. We subsequently extracted the required amount of NHCs for use with the Chromium system (10X Genomics). Therefore, we do not have the data for the total absolute number of cells (or per gram of LN). Instead, we have data for number of cells analysed per samples. We added these data in Supplemental Fig. 1c.

9) **p.3, l3: plasma cells and dendritic cells are also leukocytes.**

Response:
Thank you for this attentive indication. We revised the description from "leukocyte" to "lymphocyte" (page #5, line #56, Fig. 1b).

10) **Fig. 3e is highly useful, but should include also data from PMID: 32251437 and PMID: 33333021. Similar comparative analyses between mouse and human blood EC and non-EC stromal cell types would be helpful.**

Response:
Thank you for this comment. We added data from PMID: 32251437, which also investigated the heterogeneity of mouse LN LECs (Figure 3e). Unfortunately, PMID: 33333021 included low number of LECs and two LEC clusters; therefore, we could not perform detailed comparison with our data. As the Reviewer suggested, we compared single-cell analysis results between the present study and recent studies that investigated the heterogeneity of mouse LN BECs (Brulois et al., *Nat Commun*, 2020. PMID: 32732867) and NESCs (Rodda et al., *Immunity*, 2018. PMID: 29752062) (Extended Data Fig. 2k, 2l, 4q, 4r), highlighting unique heterogeneity in human LNs.

11) Bridge-LECs are shown to constitute about 20% of LN total lymphatic ECs (Fig 3b). Immunostainings are needed to substantiate that these EC lining the minute cord-like transsinusoidal bridges are indeed as prevalent as floor and medullary LECs.

Response:

Thank you for this comment. As the Reviewer indicated, we performed IF staining to confirm the existence and topological localization of bLEC by using an antibody against PAI1, which is encoded by *SERPINE1*, a top DEG for bLECs. We found that PAI1-positive LECs were localized exclusively at the middle zone of the subcapsular sinus, consistent with the expected localization of bLECs (Supplementary Notes page #6, lines #83–84, Extended Data Fig. 3i).

12) What is the numbering used for the citations in Suppl. text?

Response:

Thank you for this comment. The numbering used for the citations in the Suppl. text was the same used in the main manuscript, but this was a little confusing, as the Reviewer pointed out. Therefore, we made an independent citation list for Supplemental Information and changed the numbering in the Supplementary Notes accordingly.

Responses to comments of Reviewer #3:

1) **The authors state in several places that their atlas is "comprehensive". This seems to me to be extremely difficult to prove. How do they know that they have not missed subtle or rare cell types? How do they know that they have not missed cell types which are not distinguishable based solely on transcriptome? They essentially walk back the claim of being comprehensive when discussing limitations towards the end of the paper and therefore they should not make it in the first place.**

   Response:
   Thank you for this insightful comment. We used the word "comprehensive" in the original manuscript to represent the study including the analysis of not only a particular NHC component (e.g. BECs) but all major NHC components over mesenchymal stromal and endothelial cells. However, as the Reviewer pointed out, this expression may be confusing. We therefore removed the word "comprehensive" from the revised manuscript.

2) **The authors state that they have developed and approach to handle "clinical samples often difficult to assay". They do not appear to me to have advanced the state of the art in this manner - there are many examples of this in the literature by now.**

   Response:
   Thank you for this comment. We agree with the Reviewer's indication and have removed this statement from the revised manuscript.

3) **The reading is somewhat dry and the paper appears to be a laundry list of putative cell types- as the authors themselves point out, they have not done any work to functionally validate their claims. This seems to be an especially strong defect in the cell-communication analysis using cellphoneDB, which is entirely informatic and speculative.**

   Response:
   Thank you for this constructive comment. As suggested, based on the results of *CellPhoneDB* analysis, we sought to identify an interaction that could potentially be targeted in lymphoma research. We carefully surveyed all candidate interactions proposed by *CellPhoneDB* interactome analysis from the viewpoint of novelty in the lymphoma field, as well as availability of validated reagents for functional assays. We noted that among candidate interactions, the CD70–CD27 interaction has recently been attracting attention in cancer research in the context of interplays across immune cells and cancer

cells, whereas the roles of stroma-derived CD70 have scarcely been analysed. We also found a well-validated function-blocking antibody against the ligand of CD70 (CD27) (R & D systems), although no blocking antibody against CD70 was available. We thus performed binding assay experiments to validate the CD70–CD27 interaction across FL stroma and malignant B cells. We found that CD70 and CD27 were upregulated in FL medullary/adventitia stromal cells and malignant B cells, respectively. Thereafter, we confirmed binding of malignant B cells to CD70 using *in vitro* binding assays. Furthermore, the binding was inhibited by a function-blocking antibody against CD27. We also performed *ex vivo* adhesion assays using frozen FL sections and cryopreserved FL cells in conjunction with anti-CD27 antibody, and found that FL cells binding to medullary lesions were inhibited by anti-CD27 antibody. These results are described in the Results section (pages #17–18, lines #279–301, Fig. 6a–g; Extended Data Fig. 8e–g). We also added comments regarding these results in the Discussion section (pages #22–23, lines #371–379). These findings collectively support the resource value of our single-cell analysis and provide novel insights into lymphoma stroma, suggesting a potential therapeutic target of FL.

4) **It is by now well known that when using the 10x system there is RNA from lysed cells which cross-contaminates the droplets. There have been a few published examples of how to correct for this. The authors should either perform such a correction or show that it is not necessary.**

Response:

Thank you for this comment. We purified cells by FACS sorting immediately before inputting them into the 10X system. Therefore, the potential risk of including ambient RNA from lysed cells in the present study is comparable to that of many recent single-cell RNA sequencing studies that used the 10X system without particular corrections (Takeda et al., *Immunity*, 2019. PMID: 31402260; Rodda et al., *Immunity*, 2018. PMID: 29752062; Brulois et al., *Nat Commun*, 2020. PMID: 32732867).

We checked this point in all MFLN and FL samples and in their integrated data by using the *SoupX* package (Young et al., *Gigascience*, 2020. PMID: 33367645) and found that only 0–3 negligible genes (<0.01% of all genes) that were not associated with clustering results were removed from Seurat objects by *SoupX*. Furthermore, we found that clustering and DEG analysis results with or without *SoupX* correction were quite similar (data not shown). Furthermore, we used the *DecontX* pipeline (Yang et al., *Genome Biol*, 2020. PMID: 32138770) to detect potentially contaminated ambient RNA in all samples. We confirmed that NHC data had been negligibly contaminated by ambient RNA (shown below). We added a succinct description regarding this point in the Methods section (page #28, lines #463–466).

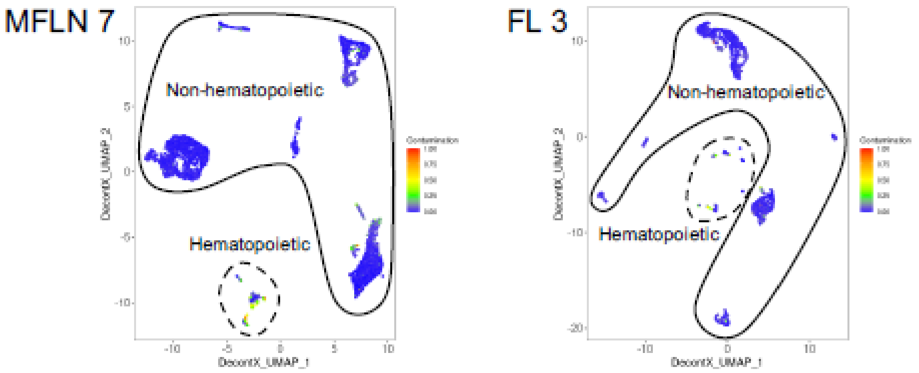

Figure legend: UMAP plot of representative cases (MFLN 7 and FL 3) showing ambient RNA contamination calculated by *DecontX* pipeline.

5) The analysis of the pathological samples seems incomplete. What are the gene expression signatures that are clearly neoplastic, especially in the B cells? Can the authors identify genomic alterations related to cancer in their transcriptome data from those samples?

Response:

Thank you for this comment. As suggested, we checked the expressions of historically known markers which are pathologically specific to FL malignant B cells, including *MME* (which encodes CD10) and *BCL2*. As expected, *MME* and *BCL2* were upregulated in malignant B cells compared with non-malignant B cells. Additionally, we performed signature analysis using the *GSVA* pipeline to further characterize malignant B-cells in FL samples (pages #32–33, lines #535–542). We developed a malignant B-cell signature (Supplementary Table 5), which included *MME* and *BCL2*, based on the recent single-cell analysis of FL haematopoietic cells (Andor et al., *Blood*, 2019. PMID: 30591526). As expected, malignant B cells detected by the discrepancy of the proportion of cells expressing *IGKC* or *IGLC2* were significantly enriched with malignant B-cell signatures (Extended Data Fig. 8c, 8d).

According to the Reviewer's suggestion, we also performed whole exome sequencing of 9 FL samples (FL 2–10) (page #39, lines #651–663). We added the somatic mutation profiles of FL samples used in the present study (Extended Data Fig. 1c; Supplementary Table 1). Unfortunately, we could not find remarkable relationships between the genomic alterations and NHC heterogeneities observed in the transcriptome data, probably because of the small cohort size.

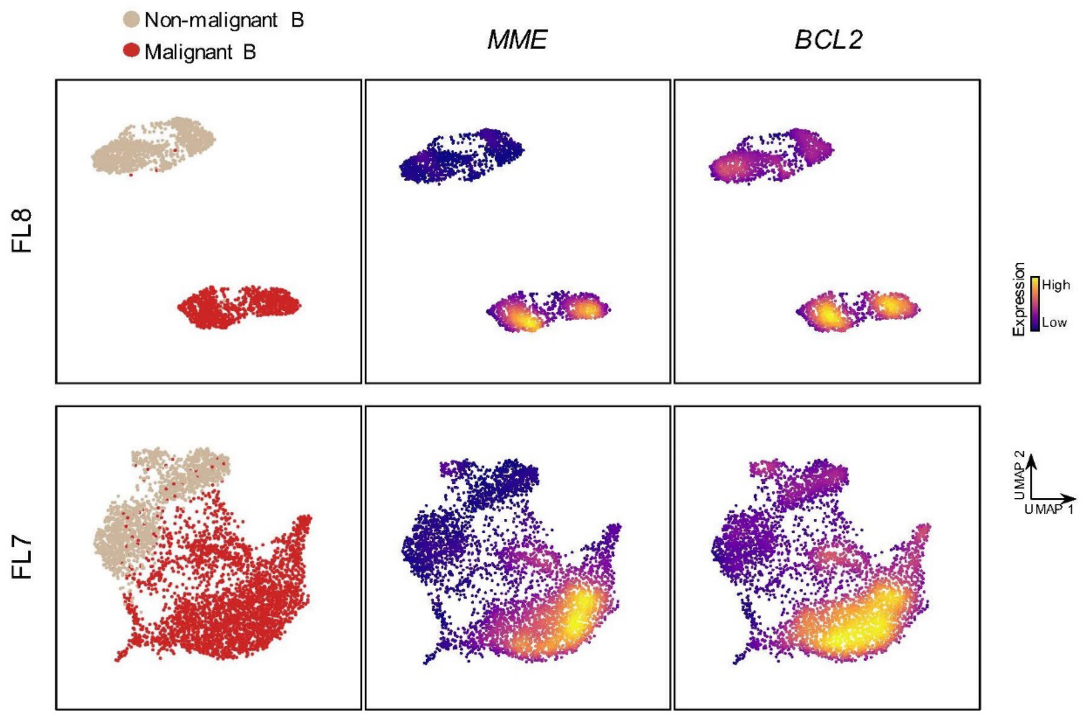

Figure legend: UMAP plots of B cells from representative FL cases (FL 8 and 7) showing B-cell type (left) and expressions of *MME* (middle) and *BCL2* (right)

Decision Letter, first revision:

Subject: Your manuscript, NCB-A45004A
Message: Our ref: NCB-A45004A

15th December 2021

Dear Dr. Sakata-Yanagimoto,

Please first accept our apology for the delay getting back to you with a decision because of the difficulty in retrieving reviewer comments.

Thank you for submitting your revised manuscript "A single-cell atlas of non-haematopoietic cells in human lymph nodes and lymphoma reveals a landscape of stromal remodelling" (NCB-A45004A). It has now been seen by the original Reviewer 2 and their comments are below. Please note that the original

Reviewer 1 and 3 were not responsive to our request to re-review, and as such we had asked the original Reviewer 2 to cross-comment on you rebuttal.

Overall the reviewer finds that the paper has improved in revision, and therefore we'll be happy in principle to publish it in Nature Cell Biology, pending minor revisions to satisfy the referees' final requests and to comply with our editorial and formatting guidelines.

If the current version of your manuscript is in a PDF format, please email us a copy of the file in an editable format (Microsoft Word or LaTex)-- we can not proceed with PDFs at this stage.

We are now performing detailed checks on your paper and will send you a checklist detailing our editorial and formatting requirements in about a week. Please do not upload the final materials and make any revisions until you receive this additional information from us.

Thank you again for your interest in Nature Cell Biology Please do not hesitate to contact me if you have any questions.

Sincerely,

Zhe Wang, PhD
Senior Editor
Nature Cell Biology

Tel: +44 (0) 207 843 4924
email: zhe.wang@nature.com

Reviewer #1 (Remarks to the Author):
Reviewer #3 (Remarks to the Author):

[Please note that Reviewer #1 and #3 was not responsive to our request to re-review, and as such Reviewer #2 was asked to cross-comment on your response to Reviewer #1 and #3.]

In my opinion, the authors have done a very good job in addressing the reviewers' requests by performing extensive new experimentation, re-analyzing their old data, rewording the text and modifying the figures. Now when the authors have successfully addressed all issues raised by the three reviewers I would be happy to see this article published.

The minor issues listed below could possibly still be modified (but I don't find them as any kind of hindrance to publication). In all these cases the authors have performed the requested experiments and

have given good responses in the rebuttal letter, but I didn't find the same responses in the manuscript itself. However, I think this information would be useful to the readers, and therefore it would be good to have the main conclusions shortly stated also in the article.

Reviewer 1:
Comment 3): The expression of BAFF, IL-15 and HGF has been now analyzed across the NHC subtypes, but it would be good to include the requested comment on their expression pattern in the actual manuscript.
Comment 7): All requested expression analyses have been done, but the explanation for the selection of the genes shown in Fig. 5c (i.e. top3 DEGs chosen for further analyses) could be included in the actual manuscript for clarity.
Comment 15): It could be shortly stated in the actual manuscript that "the prognostic impact of upregulation of TCR signature was not statistically significant".

Reviewer 3:
Comment 5): The whole exome sequencing of 9 FL samples has been performed and the data are shown. The final conclusion "We did not find relationships between the genomic alterations and NHC heterogeneities in the transcriptome data, possibly because of the small cohort size" would be good to include in the Discussion as a response to the Reviewer's question."

Reviewer #2 (Remarks to the Author):

The authors have made extensive revisions and successfully addressed most of my original concerns/questions.

The only remaining issue is that the two figures now provided only in the rebuttal letter in relation to point 6 (showing overall survival analyses), should be included in Extended data figure 9 of the manuscript itself. They are highly relevant to the readers when interpreting the current findings and older literature.

Decision letter, final requests:

Subject:	NCB: Your manuscript, NCB-A45004A
Message:	Our ref: NCB-A45004A

4th January 2022

Dear Dr. Sakata-Yanagimoto,

Thank you for your patience as we've prepared the guidelines for final submission of your Nature Cell Biology manuscript, "A single-cell atlas of non-haematopoietic cells in human lymph nodes and lymphoma reveals a landscape of stromal remodelling" (NCB-A45004A). Please carefully follow the step-by-step instructions provided in the attached file, and add a response in each row of the table to indicate the changes that you have made. Ensuring that each point is addressed will help to ensure that your revised manuscript can be swiftly handed over to our production team.

We would like to start working on your revised paper, with all of the requested files and forms, as soon as possible (preferably within one week). Please get in contact with us if you anticipate delays.

When you upload your final materials, please include a point-by-point response to any remaining reviewer comments.

If you have not done so already, please alert us to any related manuscripts from your group that are under consideration or in press at other journals, or are being written up for submission to other journals (see: https://www.nature.com/nature-research/editorial-policies/plagiarism#policy-on-duplicate-publication for details).

In recognition of the time and expertise our reviewers provide to Nature Cell Biology's editorial process, we would like to formally acknowledge their contribution to the external peer review of your manuscript entitled "A single-cell atlas of non-haematopoietic cells in human lymph nodes and lymphoma reveals a landscape of stromal remodelling". For those reviewers who give their assent, we will be publishing their names alongside the published article.

Nature Cell Biology offers a Transparent Peer Review option for new original research manuscripts submitted after December 1st, 2019. As part of this initiative, we encourage our authors to support increased transparency into the peer review process by agreeing to have the reviewer comments, author rebuttal letters, and editorial decision letters published as a Supplementary item. When you submit your final files please clearly state in your cover letter whether or not you would like to participate in this initiative. Please note that failure to state your preference will result in delays in accepting your manuscript for publication.

Cover suggestions

As you prepare your final files we encourage you to consider whether you have any images or illustrations that may be appropriate for use on the cover of Nature Cell Biology.

Covers should be both aesthetically appealing and scientifically relevant, and should be supplied at the best quality available. Due to the prominence of these images, we do not generally select images featuring faces, children, text, graphs, schematic drawings, or collages on our covers.

We accept TIFF, JPEG, PNG or PSD file formats (a layered PSD file would be ideal), and the image should be at least 300ppi resolution (preferably 600-1200 ppi), in CMYK colour mode.

If your image is selected, we may also use it on the journal website as a banner image, and may need to make artistic alterations to fit our journal style.

Please submit your suggestions, clearly labeled, along with your final files. We'll be in touch if more information is needed.

Nature Cell Biology has now transitioned to a unified Rights Collection system which will allow our Author Services team to quickly and easily collect the rights and permissions required to publish your work. Approximately 10 days after your paper is formally accepted, you will receive an email in providing you with a link to complete the grant of rights. If your paper is eligible for Open Access, our Author Services team will also be in touch regarding any additional information that may be required to arrange payment for your article.

Please note that Nature Cell Biology is a Transformative Journal (TJ). Authors may publish their research with us through the traditional subscription access route or make their paper immediately open access through payment of an article-processing charge (APC). Authors will not be required to make a final decision about access to their article until it has been accepted. Find out more about Transformative Journals

Authors may need to take specific actions to achieve compliance with funder and institutional open access mandates. For submissions from January 2021, if your research is supported by a funder that requires immediate open access (e.g. according to Plan S principles) then you should select the gold OA route, and we will direct you to the compliant route where possible. For authors selecting the subscription publication route our standard licensing terms will need to be accepted, including our self-archiving policies. Those standard licensing terms will supersede any other terms that the author or any third party may assert apply to any version of the manuscript.

Please note that you will not receive your proofs until the publishing agreement has been received through our system.

For information regarding our different publishing models please see our Transformative Journals page. If you have any questions about costs, Open Access requirements, or our legal forms, please contact ASJournals@springernature.com.

Please use the following link for uploading these materials:
[REDACTED]

If you have any further questions, please feel free to contact us. Many thanks!

Best regards,

Ziqian Li
Editorial Assistant
Nature Cell Biology

On behalf of

Zhe Wang, PhD
Senior Editor
Nature Cell Biology

Tel: +44 (0) 207 843 4924
email: zhe.wang@nature.com

Reviewer #1:
None

Reviewer #2:
Remarks to the Author:
The authors have made extensive revisions and successfully addressed most of my original concerns/questions.

The only remaining issue is that the two figures now provided only in the rebuttal letter in relation to point 6 (showing overall survival analyses), should be included in Extended data figure 9 of the manuscript itself. They are highly relevant to the readers when interpreting the current findings and older literature.

Reviewer #3:
None

Author Rebuttal, first revision:

Responses to the Reviewers' comments

NCB-A45004B: "A single-cell atlas of non-haematopoietic cells in humanlymph nodes and lymphoma reveals a landscape of stromal remodelling"

We would like to thank the reviewers for their careful reading, helpful comments, and constructive criticisms, which have significantly improved our manuscript. We have carefullyconsidered all the comments from the reviewers and revised our manuscript accordingly. In the following section, we summarize our responses to the comments from the reviewers. We believe that our responses have appropriately addressed all the concerns of the reviewers. Wesincerely hope that the revised manuscript can now be accepted for publication.

Responses to cross-comments for Reviewer #1:

1) **The expression of BAFF, IL-15 and HGF has been now analyzed across the NHC subtypes, but it would be good to include the requested comment on their expressionpattern in the actual manuscript.**

Response:
Thank you for this excellent suggestion. We have added a sentence regarding the expression patterns of *TNFSF13B* (for BAFF), *IL15* (for IL-15) and *HGF* (for HGF) in theResults section (page #14, lines #242–245) of the revised manuscript.

2) All requested expression analyses have been done, but the explanation for the selection of the genes shown in Fig. 5c (i.e. top3 DEGs chosen for further analyses)could be included in the actual manuscript for clarity.

Response:

Thank you for the comment. We completely agree with the suggestion and have now included the explanation justifying the selection of genes enlisted in Fig. 5c in the Resultssection (page #13, lines #238–239) of the revised manuscript.

3) It could be shortly stated in the actual manuscript that "the prognostic impact ofupregulation of TRC signature was not statistically significant".

Response:

Thank you for this suggestion. As indicated, we have added the relevant information regarding the prognostic analysis of TRC signature in the Discussion section (page #21, lines #380–382) of the revised manuscript and Supplementary Notes (SupplementaryNotes page #10, lines #145–149).

Responses to comments of Reviewer #2:

The authors have made extensive revisions and successfully addressed most of my original concerns/questions. The only remaining issue is that the two figures now provided only in the rebuttal letter in relation to point 6 (showing overall survival analyses), should be included in Extended data figure 9 of the manuscript itself. Theyare highly relevant to the readers when interpreting the current findings and older literature.

Response:

Thank you for the kind suggestion. As recommended, we have now included two figures

demonstrating the survival outcomes in the Extended Data Figure 9 (Extended Data Fig. 9f,g)and accordingly added the relevant information pertaining to this in Supplementary Notes (Supplementary Notes page #9, lines #140–144; page #10, lines #150–154) .

Responses to cross-comments for Reviewer #3:

1) The whole exome sequencing of 9 FL samples has been performed and the data are shown. The final conclusion "We did not find relationships between the genomic alterations and NHC heterogeneities in the transcriptome data, possibly because of the small cohort size" would be good to include in the Discussion as a response to theReviewer's question."

Response:
Thank you for your thoughtful suggestion. We have now added the relevant information about the interpretation of the results of whole exome sequencing in the paragraph about the limitations of the study in the Discussion section (page #23, lines #402–404) of the revised manuscript.

Final Decision Letter:

Subject: Decision on Nature Cell Biology submission NCB-A45004B
Message: Dear Dr Sakata-Yanagimoto,

I am pleased to inform you that your manuscript, "A single-cell atlas of non-haematopoietic cells in human lymph nodes and lymphoma reveals a landscape of stromal remodelling", has now been accepted for publication in Nature Cell Biology.

Thank you for sending us the final manuscript files to be processed for print and online production, and for returning the manuscript checklists and other forms. Your manuscript will now be passed to our production team who will be in contact with you if there are any questions with the production quality of supplied figures and text.

Over the next few weeks, your paper will be copyedited to ensure that it conforms to Nature Cell Biology style. Once your paper is typeset, you will receive an email with a link to choose the appropriate publishing options for your paper and our Author Services team will be in touch regarding any additional information that may be required.

After the grant of rights is completed, you will receive a link to your electronic proof via email with a request to make any corrections within 48 hours. If, when you receive your proof, you cannot meet this deadline, please inform us at rjsproduction@springernature.com immediately.

You will not receive your proofs until the publishing agreement has been received through our system.

Due to the importance of these deadlines, we ask that you please let us know now whether you will be difficult to contact over the next month. If this is the case, we ask you provide us with the contact information (email, phone and fax) of someone who will be able to check the proofs on your behalf, and who will be available to address any last-minute problems.

If you have any questions about our publishing options, costs, Open Access requirements, or our legal forms, please contact ASJournals@springernature.com

Once your paper has been scheduled for online publication, the Nature press office will be in touch to confirm the details. An online order form for reprints of your paper is available at https://www.nature.com/reprints/author-reprints.html. All co-authors, authors' institutions and authors' funding agencies can order reprints using the form appropriate to their geographical region.

Publication is conditional on the manuscript not being published elsewhere and on there being no announcement of this work to any media outlet until the online publication date in Nature Cell Biology.

Please note that Nature Cell Biology is a Transformative Journal (TJ). Authors may publish their research with us through the traditional subscription access route or make their paper immediately open access through payment of an article-processing charge (APC). Authors will not be required to make a final decision about access to their article until it has been accepted. Find out more about Transformative Journals

Authors may need to take specific actions to achieve compliance with funder and institutional open access mandates. For submissions from January 2021, if your research is supported by a funder that requires immediate open access (e.g. according to Plan S principles) then you should select the gold OA route, and we will direct you to the compliant route where possible. For authors selecting the subscription publication route our standard licensing terms will need to be accepted, including our self-

archiving policies. Those standard licensing terms will supersede any other terms that the author or any third party may assert apply to any version of the manuscript.

To assist our authors in disseminating their research to the broader community, our SharedIt initiative provides you with a unique shareable link that will allow anyone (with or without a subscription) to read the published article. Recipients of the link with a subscription will also be able to download and print the PDF.

If your paper includes color figures, please be aware that in order to help cover some of the additional cost of four-color reproduction, Nature Research charges our authors a fee for the printing of their color figures. Please contact our offices for exact pricing and details.

As soon as your article is published, you will receive an automated email with your shareable link.

If you have not already done so, we strongly recommend that you upload the step-by-step protocols used in this manuscript to the Protocol Exchange (www.nature.com/protocolexchange), an open online resource established by Nature Protocols that allows researchers to share their detailed experimental know-how. All uploaded protocols are made freely available, assigned DOIs for ease of citation and are fully searchable through nature.com. Protocols and the Nature and Nature research journal papers in which they are used can be linked to one another, and this link is clearly and prominently visible in the online versions of both papers. Authors who performed the specific experiments can act as primary authors for the Protocol as they will be best placed to share the methodology details, but the Corresponding Author of the present research paper should be included as one of the authors. By uploading your Protocols to Protocol Exchange, you are enabling researchers to more readily reproduce or adapt the methodology you use, as well as increasing the visibility of your protocols and papers. You can also establish a dedicated page to collect your lab Protocols. Further information can be found at www.nature.com/protocolexchange/about

You can use a single sign-on for all your accounts, view the status of all your manuscript submissions and reviews, access usage statistics for your published articles and download a record of your refereeing activity for the Nature journals.

Please feel free to contact us if you have any questions.

With kind regards,

Zhe Wang, PhD
Senior Editor
Nature Cell Biology

Tel: +44 (0) 207 843 4924
email: zhe.wang@nature.com
